# Multilocus analysis uncovers the evolution of the Rhodniini tribe, vectors of *Trypanosoma cruzi*

**DOI:** 10.1038/s41598-025-03789-9

**Published:** 2025-07-01

**Authors:** Carolina Hernández, Fabian C. Salgado-Roa, Carolina Pardo-Diaz, João Aristeu da Rosa, Jader Oliveira, Cleber Galvão, Simone Patrícia Carneiro Freitas, Jose E. Calzada, Lineth Garcia, Mario J. Grijalva, Anita G. Villacís, Hernan Carrasco, Maikell Segovia, Cesar Gomez Hernandez, Plutarco Urbano, Omar Cantillo-Barraza, Felipe Guhl, Julio Cesar Carranza, Kaio Cesar Chaboli Alevi, Claudia Sandoval, Alberto Paniz-Mondolfi, Gustavo Vallejo, Camilo Salazar, Juan David Ramírez

**Affiliations:** 1 Centro de Investigaciones en Microbiología y Biotecnología-UR (CIMIBIUR), School of Sciences and Engineering, Universidad del Rosario, Bogotá, Colombia; 2 Molecular Microbiology Laboratory, Department of Pathology, Molecular and Cell-Based Medicine, Icahn School of Medicine at Mount Sinai, New York, NY 10029 USA; 3 Biology Program, School of Sciences and Engineering, Universidad del Rosario, 111221 Bogotá, Colombia; 4 School of BioSciences, The University of Melbourne, Parkville, VIC 3052 Australia; 5 Faculdade de Ciências Farmacêuticas, Universidade Estadual Paulista (UNESP), Araraquara, Sao Paulo 01000 Brazil; 6 Faculdade de Saúde Pública, Universidade de São Paulo (USP), São Paulo, SP Brazil; 7 Instituto Oswaldo Cruz, Rio de Janeiro, Brazil; 8 Fundação Oswaldo Cruz, Escritório Regional Piauí, Piauí, Brazil; 9 Departamento de Investigación en Parasitología, Instituto Conmemorativo Gorgas de Estudios de La Salud, Panama City, Panamá; 10 Universidad Mayor de San Simón, Cochabamba, Bolivia; 11 Centro de Investigación Para La Salud en América Latina, Facultad de Ciencias Exactas y Naturales, Pontificia Universidad Católica del Ecuador, Quito, Ecuador; 12 Sección de Epidemiología Molecular, Instituto de Medicina Tropical, Facultad de Medicina, Universidad Central de Venezuela, Caracas, Venezuela; 13 Universidade Federal Do Triangulo Mineiro (UFTM), Uberaba, Brazil; 14 Grupo de Investigaciones Biológicas de La Orinoquia, Fundación, Universitaria Internacional del Trópico Americano (Unitrópico), Yopal, Colombia; 15 Grupo BCEI, Universidad de Antioquia, Medellín, Colombia; 16 Centro de Investigaciones en Microbiología y Parasitología Tropical (CIMPAT), Departamento de Ciencias Biológicas, Facultad de Ciencias, Universidad de los Andes, 111711 Bogotá, Colombia; 17 Laboratorio de Investigaciones en Parasitología Tropical (LIPT), Universidad del Tolima, 730001 Ibagué, Colombia; 18 Grupo de Investigaciones en Ciencias Básicas y Aplicadas Para La Sostenibilidad (CIBAS), Facultad de Ciencias Exactas, Naturales y Agropecuarias, Universidad de Santander, Bucaramanga, Colombia

**Keywords:** Rhodniini tribe, Chagas disease, Phylogenetic analysis, Multilocus analysis, Evolutionary history, Genetic structure, Vector-borne diseases, Pleistocene arc hypothesis, Insect vectors, *Psammolestes*, *Rhodnius*, Phylogenetic discordances, Population genetics, Speciation patterns, Molecular biology, Zoology

## Abstract

In this study, we investigate the origin and diversification of *Trypanosoma cruzi* vectors within the Rhodniini tribe (Triatominae subfamily) through phylogenetic analyses based on eight genes from 17 species and 497 specimens—the largest sampling of this tribe to date. Our results predominantly support the paraphyly of the genus *Rhodnius*, with the three *Psammolestes* species forming a well-supported monophyletic clade nested within it. In two reconstructions, however, *Psammolestes* and *Rhodnius* are recovered as reciprocally monophyletic, each with strong support. In *Rhodnius*, we find monophyletic *pallescens* and *pictipes* groups, but a paraphyletic *prolixus* group, with persistent phylogenetic discordances underscoring uncertainties in species placements. Divergence estimates suggest Rhodniini originated around 5.26 million years ago, notably more recent than previously thought. Evolution within the tribe appears shaped by geography, gene flow, and incomplete lineage sorting rather than traditional taxonomy. Only four species—*P. arthuri*, *R. ecuadoriensis*, *R. neivai*, and *R. neglectus*—are consistently supported across analyses, likely diversifying during Pleistocene climate changes. Other Rhodniini species may represent a panmictic population with minor structuring influenced by the Andes uplift. This study underscores the need for integrative research combining genetic, ecological, and biogeographical data to fully understand Rhodniini speciation and diversification.

## Introduction

Insect vectors play a crucial role in the dynamics of disease transmission owing to their complex interactions with both pathogens and hosts^1^. Therefore, understanding their taxonomy and evolutionary history is essential for evaluating their adaptability, dispersal capacity, and insecticide resistance, as well as for designing effective control strategies^2,3^. This is better achieved by implementing integrative approaches that combine genetic, ecological, and morphological data^2,4^. This has been done in mosquitoes, where even the history of host use has been studied through phylogenomics to understand their co-evolution^5^. Also, control strategies for Malaria and Dengue have been improved by genomic analyses of *Anopheles* Meigen, 1818 (Diptera: Culicidae)^6^ and *Aedes* (Linnaeus, 1762) (Diptera: Culicidae)^7^ mosquitoes, respectively.

Evolutionary processes such as introgression, ecological selection, symbiotic relationships, sexual selection, chromosome rearrangements, and genetic incompatibilities are known to significantly contribute to the diversification of insect lineages^8,9^. Demographic and climatic changes also play their part in this process^10^. However, we still know little about the adaptation and speciation patterns of insects in the Neotropics^11^, not even for vector insects, which affects our understanding of the dynamics of vector-borne tropical diseases.

*Trypanosoma cruzi* (Chagas, 1909) (Kinetoplastida: Trypanosomatidae) is an insect-borne parasite that causes Chagas disease, a disease that predominantly affects Latin American countries leading to 6 to 8 million infections and ~ 50,000 deaths each year^12^. The primary mode of transmission implies humans coming into contact with feces of infected Triatominae (Hemiptera: Reduviidae: Triatominae), a subfamily of insects composed by five tribes where Triatomini and Rhodniini are the most relevant in terms of transmission of the parasite^13^. In particular, species in the Rhodniini tribe are major transmitters of *T. cruzi*^13^ with an extensive geographic distribution. The tribe is highly diverse and comprises 22 species, three in the genus *Psammolestes* Bergroth, 1911 (*P. arthuri*, *P. coreodes* and *P. tertius*) and 19 in the genus *Rhodnius* Stål, 1859^14^. Within *Rhodnius,* species have been classified into three groups (*prolixus*, *pictipes*, and *pallescens*) based on morphology, distribution, biology, and ecology^13^. Four species in the genus are known primary vectors of *T. cruzi: R*. *prolixus* Stål, 1859*, R*. *stali* Lent, Jurberg & Galvão, 1993*, R*. *ecuadoriensis* Lent & León, 1958*,* and* R*. *pallescens* Barber, 1932^15^, while others such as *R. prolixus* Stål, 1859, *R. robustus* Larrousse, 1927, *R. montenegrensis* Rosa et al., 2012*, R. neglectus* Lent, 1954, *R. neivai* Lent, 1953, *R. nasutus* Stål, 1859 , *R. brethesi* Matta, 1919, *R. pictipes* Stål, 1872, *R. colombiensis* Mejía, Galvão & Jurberg, 1999, and* P*. *arthuri* Pinto, 1926 have been observed in domestic environments, suggesting they might play a role in the transmission of the parasite^14^.

Various studies have investigated the evolution and classification of the Rhodniini tribe using both classical and modern techniques including taxonomy, cytogenetics, isoenzymes, single loci genetics, transcriptomics, and genomics. Although these investigations have resolved multiple taxonomic conflicts^14,16–19^, there are still inconsistencies in species’ relationships. These include: (i) the paraphyly of *Rhodnius* with respect to *Psammolestes*; (ii) discrepancies in the species grouping within *Rhodnius*; (iii) unresolved relationships between some species; and (iv) uncertainty on the species status of some lineages within *Rhodnius*^14,18–20^. A recent phylogenomic analysis of a large dataset comprising 36 specimens from 17 species sought to clarify taxonomic relationships within the tribe^18^, but despite the extensive taxonomic sampling, taxonomic conflicts and discrepancies between mitochondrial and nuclear loci persisted. Also, not all species and a few individuals were included. Therefore, there still exist conflicting hypotheses on the origin, times of diversification, biogeography, population structure and genetic connectivity within Rhodniini^14,16–19^.

Due to variations in vectorial capacity among triatomine species, accurate identification is a crucial step for the success of vector surveillance and control programs ^2^.To address these questions, here we investigate the phylogenetic and evolutionary history of the Rhodniini tribe based on eight loci (including cytochrome b and 28S rRNA^17,21^) as well as six nuclear single-copy genes. These were amplified in 497 specimens from 17 species collected in seven countries, thus being the largest genetic, taxonomic and geographic sampling ever done in the Rhodniini tribe.

## Results

**Fig. 1:**
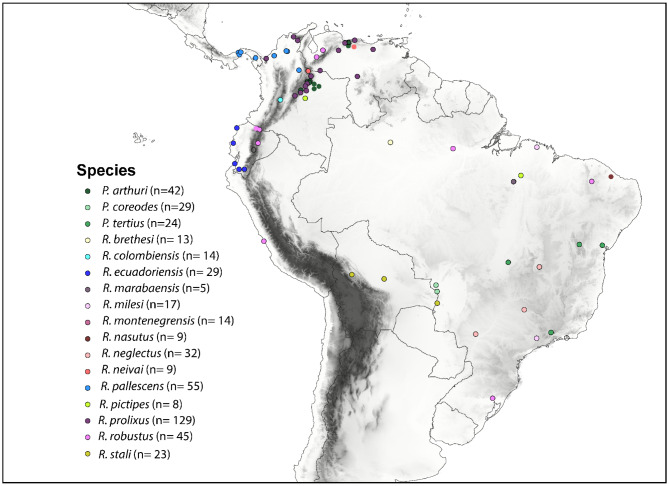
Geographic distribution of the species in the Rhodniini tribe. The map shows the distribution of the 17 species included in this study, with dots indicating sampling locations. Each species is color coded. The map was generated using the free and open-source QGIS software, with a base shapefile obtained from Natural Earth (https://www.naturalearthdata.com/downloads/10m-cultural-vectors/) and an elevation raster from SRTM (https://csidotinfo.wordpress.com/data/srtm-90m-digital-elevation-database-v4-1/).

A total of 497 individuals from 17 Rhodniini species were collected across seven countries in Central and South America, covering the tribe’s regional distribution (Fig. 1, Figures S1-S4). This included key species like *R. pallescens*, *R. robustus*, and *R. prolixus*, among others (Table S1). Four *Panstrongylus geniculatus* and two *Triatoma dimidiata* specimens were included as outgroups. All samples were preserved in ethanol at 2–8 °C.

### Molecular phylogenetics of Rhodniini tribe

**Fig. 2:**
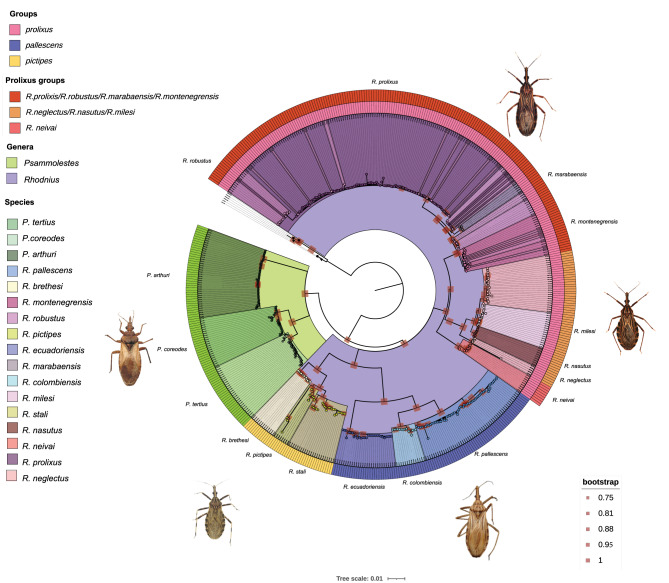
Maximum credibility phylogenetic reconstruction of the Rhodniini tribe. This figure shows the maximum credibility phylogenetic reconstruction of the Rhodniini tribe based on a concatenated alignment (size = 4368 bp) of the eight loci used in this study (nuclear, ribosomal, and mitochondrial). The reconstruction was performed using the Maximum Likelihood algorithm with PHYML and 1000 bootstrap repetitions. Bootstrap values are indicated by red squares, with only nodes having bootstrap values greater than 95% shown. Different genera, species, groups, and clades are represented by distinct colors The rings surrounding the phylogeny represent the groups of the genus *Rhodnius* and the clades of the *prolixus* group.

The PhyML topology from the full alignment revealed two monophyletic clades (Fig. 2). One includes all *Psammolestes* species, with *P. arthuri* as sister to *P. tertius* and *P. coreodes* (Bootstrap value > 95.0%). The second clade comprises all *Rhodnius* species, divided into three well-supported groups: *pictipes*, *pallescens*, and *prolixus* (Bootstrap value > 95.0%). Within *pictipes*, *R. stali* and *R. pictipes* form a clade sister to *R. brethesi*, while in *pallescens*, *R. pallescens* and *R. colombiensis* cluster together as sister to *R. ecuadoriensis* (Fig. 2). The *prolixus* group is sister to the *pictipes* + *pallescens* clade (Bootstrap value > 95%) and includes two main subclades: one where *R. neivai* is monophyletic, and another where *R. neglectus* is paraphyletic with *R. nasutus* and *R. milesi*, while *R. marabaensis* is monophyletic. In contrast, *R. prolixus*, *R. robustus*, and *R. montenegrensis* do not form monophyletic lineages.

**Fig. 3:**
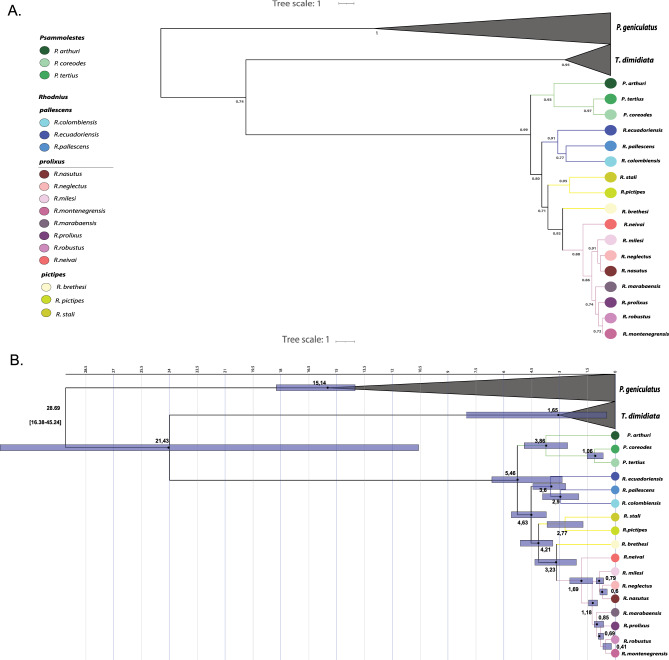
Species tree and divergence time estimation based on multilocus data. (**A**) Species tree inferred from Bayesian analysis; posterior probabilities are shown as red squares with varying sizes. (**B**) Divergence time estimation; purple bars represent the 95% highest posterior density (HPD) intervals for node divergence times. Species and genera are color coded.

Phylogenies inferred using IQ-Tree and FastTree (Figures S5–S6) showed some discrepancies with the PhyML topology. Bayesian and coalescent analyses (Figures S7–S8) failed to fully resolve relationships among *pictipes*, *pallescens*, and *prolixus*, or between *Psammolestes* and *Rhodnius*. However, the StarBEAST2 multispecies coalescent tree (Fig. 3A) supported *Psammolestes* as sister to *Rhodnius*.

Nuclear-gene-based analyses (IQ-Tree, FastTree, PhyML, and MrBayes; Figures S9–S12) recovered a topology consistent with the full alignment IQ-Tree analysis (Figure S5). ASTRAL (Figure S13) produced a topology similar to FastTree (Figure S6), while the StarBEAST2 tree with nuclear loci (Figure S14) placed *Psammolestes* as sister to *prolixus*, which was sister to *pallescens*, and all of them were sister to *pictipes*.

Single-locus analyses consistently supported *pictipes* and *pallescens* as distinct from *prolixus* (Figures S15–S21), while *Psammolestes* clustered with *prolixus*. Mitochondrial CYTB topologies were highly consistent across methods and agreed with previous studies^14,22^ . However, in this CYTB reconstructions *R. pictipes* clusters with the *pallescens* group (albeit poorly supported; Figures S22-S26). Also, species in the *prolixus* group were paraphyletic to species of *Psammolestes* (Figures S22–S26).

Because of the topological discordances we found, we applied multiple topology tests that revealed the PhyML topology as the most strongly supported (Fig. 2), followed by the FastTree phylogeny (Figure S6, Table S3). The main difference between these is the position of the genus *Psammolestes:* while in the PhyML tree species of *Psammolestes* are sister to *Rhodnius*, in the FastTree phylogeny the *prolixus* group is paraphyletic to *Psammolestes*.

### Species delimitation and divergence times estimation in the Rhodniini tribe

**Fig. 4:**
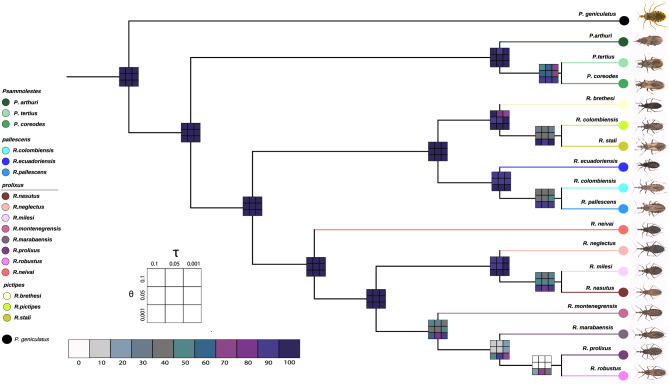
Bayesian species delimitation. Bayesian species delimitations inferred under nine different theta (θ) and tau (τ) prior combinations. The posterior probability of each of these combinations is color-coded and indicated in 3 × 3 boxes on each node of the guide tree. The large 3 × 3 inset indicates the position of each prior combination in these boxes.

Our analyses support the existence of significantly fewer species than the currently described morphospecies in the Rhodniini tribe. Only four out of the 17 species analyzed were consistently identified as distinct species across all scenarios tested (posterior probability > 90%): *P. arthuri*, *R. ecuadoriensis*, *R. neivai*, and *R. neglectus* (Fig. 4). We estimated the origin of the Rhodniini tribe ~ 5.26 million years ago (95% HPD: 2.49–6.86; Fig. 3B, Figures S27-S28) and that of the genus *Psammolestes* ~ 3.86 million years ago (95% HPD: 2.49–6.86; Fig. 3B, Figures S27-S28). *Rhodnius* began diversifying ~ 4.59 million years ago (95% HPD: 3.59–5.77; Fig. 3B, Figure S27-S28).

### Population genetics analyses

Estimates of nucleotide diversity were low in most species and loci (π < 0.01) except for the CYTB locus where *P. tertius*, *R. pallescens*, *R. robustus* and *R. neglectus* exhibited high genetic diversity (Table S2). This mitochondrial locus also revealed high nucleotide diversity in the genus *Psammolestes* and in the groups of *Rhodnius*. In contrast, estimates of nucleotide diversity obtained from nuclear loci were overall low, with some exceptions. For example, *pallescens* was the more diverse group showing high nucleotide diversity in five nuclear loci (CISP, PJH, TRNA, UPCA, and UPMETAL), while *pictipes* had high π only in two loci (CISP and TRNA) and *prolixus* only in one locus (TRNA). Also, the genus *Psammolestes* had high nucleotide diversity in three nuclear loci (LSM, TRNA and UPCA). Interestingly, six out of eight loci showed evidence of population expansion in *R. prolixus* (Table S2), although this signal does not persist when Tajima’s D was estimated for the clade containing both *R. robustus* and *R. prolixus* (Table S2).

Relative indices of genetic differentiation (F_ST_) were high both between *Psammolestes* and *Rhodnius,* and within the *Rhodnius* groups (*pictipes*, *prolixus*, and *pallescens*) (Figure S29 and S32). Although species in the *prolixus* group were less structured, the extent of structuring varied across different genes (Figures S29 and S32). Also, absolute measures of differentiation (Dxy, Da) revealed patterns largely consistent with the phylogenetic discordances described above (Fig. 2 and Figures S5–S8), where some loci show *Psammolestes* clustering with the *prolixus* group and others clustering with the *pictipes*/*pallescens* groups (Figures S30 and S31). Overall, we observed higher genetic structure between species of the *pictipes* and *pallescens* groups, while species in the *prolixus* group showed low genetic divergence (Figures S30 and S31). *Psammolestes arthuri* exhibited greater genetic differentiation compared to other species of *Psammolestes* and *Rhodnius*. In contrast, *P*. *coreodes* and *P. tertius* showed less divergence from other *Rhodnius* species in some loci (Figures S30 and S31).

**Fig. 5:**
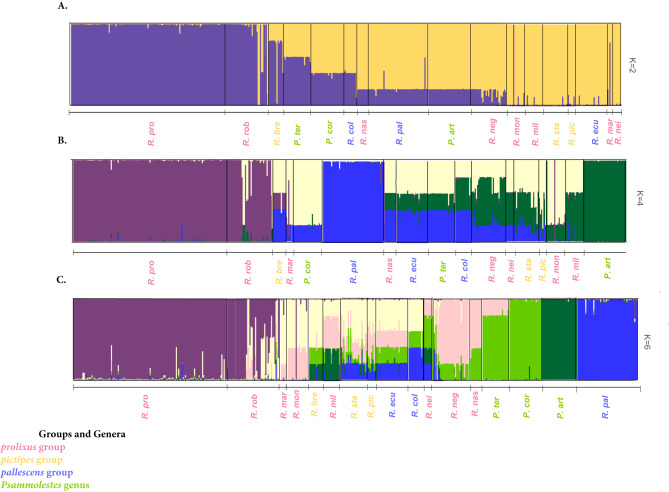
Population clustering in the Rhodniini tribe as revealed by STRUCTURE. The numbers of ancestry groups (K) are between 2 and 6. (**A**). K = 2 (**B**). K = 4 and **C.** K = 6 and the matrix of aligned Q values from individuals included in this study obtained from CLUMPP. Each bar represents an individual, and the color of the bar represents the likelihood of that individual belonging to a population.

The STRUCTURE analysis revealed K = 2 as the optimal number of clusters (K), although K = 4 and K = 6 also had good scores (Fig. 4, Table S2, and Figure S35). In K = 2, one cluster mostly includes *R. prolixus*, *R. robustus, P. tertius* and *R. brethesi*, the second includes *R. montenegrensis, R. milesi, R. stali, R. pictipes, R. ecuadoriensis, R. marabaensis* and *R. neivai*; the remaining seven species show mixed ancestry (Fig. 5A). In K = 4, *R. prolixus, R. pallescens* and *P. arthuri* were each identified as distinct groups, with the remaining species having mixed ancestry (Fig. 5B). In K = 6, *R. prolixus, R. pallescens*, *P. arthuri, P. coreodes, R. pallescens* and *R. neglectus* form independent clusters, and the remaining species showed mixed patterns (Fig. 5C).

These clusterings do not recover species groups or genera, but seem rather consistent with geography. For example, the first cluster (purple in Fig. 5) includes *R. prolixus* and *R. robustus* sampled from the foothills of the eastern cordillera in Colombia, the Eastern Andes in Ecuador, Mérida in Venezuela, and some individuals of *R. robustus* from the Peruvian West Andes, Caipiru, and Catinga in Brazil. The second cluster (blue in Fig. 5B) comprises *R. pallescens* sampled in Panama and northern Colombia, as well as an individual from the eastern Andes population of Meta. The third cluster (dark green in Fig. 5B) is formed by *P. arthuri* from the foothills of the eastern cordillera in Colombia and Mérida, Venezuela. The fourth cluster includes *P. coreodes* and *P. tertius* from Gran Chaco and the Atlantic Forest in Brazil, respectively. The fifth cluster (red in Fig. 5C) consists of *R. neglectus* and *R. nasutus* from the Cerrado and Catinga, respectively (Fig. 5C). Finally, the sixth cluster (yellow in Fig. 5C) encompasses Amazonian species that share ancestry with the other clusters. Notably, *R. neivai* and *R. ecuadoriensis* exhibit genetic variation from several clusters (Fig. 5C).

## Discussion

By conducting the most extensive molecular and taxonomic sampling of the Rhodniini tribe to date, we provide the first study to include and recover all three *Psammolestes* species. Four phylogenetic reconstructions recovered *Rhodnius* as paraphyletic with respect to *Psammolestes*, which consistently formed a monophyletic clade, a pattern consistent with previous studies^14,19,23^ . In contrast, two reconstructions supported *Psammolestes* and *Rhodnius* as phylogenetically distinct, reciprocally monophyletic genera. This last result agrees with the biological, ecological, and morphological differences that distinguish these genera^24–28^. The distinction is epidemiologically relevant, as misclassification can lead to inaccurate assumptions regarding their vectorial role, potentially affecting surveillance efforts and control strategies^2^. The eco-epidemiological characteristics of *Psammolestes* including ecological niche, *T. cruzi* infection rates and burdens, blood-feeding behavior, and microbiota composition differ markedly from those of *Rhodnius*, necessitating species-specific approaches to vector surveillance and control. Notably, *Psammolestes* exhibit distinct ecological and feeding behaviors. Unlike *Rhodnius*, which maintains close associations with mammalian hosts and has a high potential for household infestation, *Psammolestes* is primarily associated with bird nests. This specialization may reduce its direct contact with humans and limit its role in *T. cruzi* transmission^28–32^. Recognizing these differences will contribute to more accurate risk assessments and facilitate the design of tailored interventions for Chagas disease vector control^2^. Also, based on our estimation of divergence times, the Rhodniini tribe likely originated in the late Miocene to early Pliocene, an estimate that is more recent than previous reports that estimated divergence at 107 Ma^33^, 22.91^34^ and 17. 91^19^. Furthermore, we recovered a divergence time of 5.46 Ma between *Psammolestes* and *Rhodnius*, which is consistent with our previous findings for the *Psammolestes* genus^17^. Genetic structuring in the tribe is not associated with species or species groups, but rather with geography, and especially, with the bioregions delimited by phylogenetic beta diversity for Triatominae in the New World^35^.

We found the genus *Rhodnius* diversified around 4.63 Ma in a period when the Andes was largely uplifted (~ 7–5 Ma). However, its evolution and that of its groups is rather complex and seems to be shaped by geography. Different genes and phylogenetic methods revealed contrasting relationships and patterns within *Rhodnius.* For example, in some topologies the *pallescens*/*pictipes* groups appear as closely related to *R. neivai*, *Psammolestes* spp., and a group composed by *R. milesi*, *R. neglectus*, and *R. nasutus.* In other cases, these very same groups cluster with *R. prolixus*, *R. robustus*, *R. montenegrensis*, and *R. marabaensis*. Additionally, the phylogenetic position of *R*. *brethesi* was inconsistent, clustering with the *prolixus* group in the species tree (Fig. 3A), or with *R. stali* or *R. pictipes* in other analyses (Fig. 2). These discrepancies may be attributed to factors such as incomplete lineage sorting, introgression, or varying evolutionary rates among mitochondrial and nuclear loci^36^, and in fact, previous studies have reported similar mito-nuclear discordances in species of the *pictipes* group, which have been attributed to introgression^18^. The fact that most of the supported topologies find the *pictipes* group as sister to the *pallescens* group (Fig. 2), but the species tree reveals the *pictipes* group as sister to the *prolixus* group (Fig. 3A) may be the result of shared genetic variation between species occurring west of the Eastern Cordillera of Colombia (trans-Andean) and those from the east of the same mountain range (cis-Andean)^37^. These results highlight the complex evolution of these vectors, where a handful of species diverged within a short time frame resulting in shared ancestry between populations at both sides of the Andes. Similar patterns have been observed in other arthropods, birds, and parasites ^9^and have been attributed to gene flow, incomplete lineage sorting, or both. Further studies are necessary to clarify these possibilities. In agreement with the phylogenetic hypotheses and conflicts we recovered, we observed low nucleotide diversity across groups and species of *Rhodnius*. This was true for F_ST_ estimates (except for TRNA and CYTB; Figure S31) even when they are known to overestimate genetic differentiation in cases of low intra-species diversity^38^, as well as for the Da statistic that was high in CYTB but low in nuclear loci (Figs. 28 and 31). Furthermore, although the network analysis for each nuclear locus consistently recovered the groups of *Rhodnius*^13,39^, the branch lengths are short in all cases (less than 2 mutations per 100 bp), thus challenging their existence. Interestingly, the groups in *Rhodnius* (i.e. *pallescens* and *pictipes*) are supported as independent lineages (species) only in scenarios with small population sizes (θ).

The species delimitation analysis consistently supported four species within the tribe across all scenarios tested, namely: *P. arthuri*, *R. ecuadoriensis*, *R. neivai*, and *R. neglectus* (Fig. 4). This is further supported by the fact that they do not produce hybrids when crossed with closely related species (i.e., in the same group^25,40^). These four species are confined to the fragmented seasonally dry tropical forests (STDF) surrounding the Amazon basin, and its divergence time coincides with the Pleistocene Arc hypothesis (~ 2.58–0.77 Ma) which posits that climate fluctuations significantly influenced South American biodiversity ^41^ and explain the diversification of various plants^42^, birds^41,43^ and reptiles^44^. Also, the climatic and environmental conditions of the STDF likely facilitated geographic isolation and subsequent speciation of the Rhodniini within, while lineages occurring in the Amazon basin and the Andes are better connected. The influence of climatic events in the diversification of Rhodniini suggest that current climate changes may affect the distribution and evolution of its species, potentially increasing the risk of disease transmission and adaptation to domestic environments^45^. Notably, both *R. ecuadoriensis* and *R. neivai* showed high levels of mixed ancestry patterns in K = 4 and K = 6 (Fig. 4), which coupled with their limited geographical distribution and unique morphological traits, suggest their evolution as independent lineages is different from other species in the tribe (Fig. 5).All of the above support that the Rhodniini tribe comprises fewer species than previously described, and some currently accepted lineages are likely panmictic populations with substantial phenotypic variation and with geography influencing their genetic variation (Table S2). In fact, previous studies have recognized and tried to solve taxonomic inconsistencies in the tribe, and to date six synonymization events occurred: (i) *R. brumpti* Pinto, 1925 with *R. nasutus*, (ii) *R. dunni* Pinto, 1932 with *R. pallescens*, (iii) *Conorhinus limosus* Walker, 1873 with *R. pictipes* and *R. prolixus*, (iv) *R. taquarussuensis* Rosa et al., 2017 with *R. neglectus,* (v) *R. zeledoni* Jurberg, Rocha & Galvão, 2009 with *R. domesticus* Neiva & Pinto, 1923, and (vi) *R. milesi* with *R. neglectus*^21,48,49^. Interestingly, in this study we recover *R. neglectus* as a supported species. This is a taxon with a wide geographic distribution and phenotypic plasticity^49^ that recently synonymized *R. taquarussuensis* and *R. milesi* based on phylogenetic evidence and experimental crosses^48^. In this study we included only three populations of *R. neglectus* from Brazil (Goias, Minas Gerais and Tocantins – representing the polymorphism previously characterized as *R. taquarussuensis*), and with this sampling, we recovered *R. neglectus* as paraphyletic in some loci (Figures S5, S6, S7, S11, S24, S25) but monophyletic in the mitochondrial locus; the latter also recovered *R. milesi* as a single taxon (Figures S8, S9, S13, S23, S24). This has also been observed by Filée et al. ^23^ using the same mitochondrial marker. Therefore, our study highlights the need of understand the complex taxonomy and evolution of the Rhodniini tribe by investigating its speciation patterns not only with morphology or phylogenetics, but also exploring the drivers of reproductive isolation and morphometrics.

Given the tribe’s role in the transmission of *T. cruzi*, such an improved species delimitation and population genetics should be coupled with analyses of host, reservoir and parasite interactions, vector competence, adaptability to new habitats/environments to facilitate a more precise stratification of the risks associated with parasite transmission and design more targeted and effective control strategies. Thus, it is essential for strategies to account for the potential effects of climate change on the distribution of populations and species within the tribe, which may facilitate genetic exchange and consequently enhance vectorial capacity, as observed in *Triatoma* hybrids^36,50,51^.

We acknowledge several limitations in our study. First, the absence of morphometric data prevented us from correlating phenotypic variation with genetic structure. Second, the impossibility of including species such as *R. amazonicus*, *R. barreti* and *R. domesticus* limits our understanding of the evolutionary history of the tribe. Third, a deeper sampling of individuals in the Amazon would help understand if species in this region are in fact a panmictic population. Fourth, although this is the most extensive molecular study in the tribe to date, genomic data from all species and more individuals in this tribe will be crucial for identifying species and test for gene flow versus incomplete lineage sorting across the evolution of the Rhodniini^52–55^. The assembly and annotation of more genomes from the Rhodniini tribe will also allow a deeper understanding of their genetic variability, and mapping loci underlying vectorial capacity, adaptability to habitats, and host-vector and parasite-vector interactions.

Overall, our results show a new perspective on the evolution of the Rhodniini tribe. While inconsistencies previously described are still maintained, here we: (i) support the monophyly of *Psammolestes*, (ii) suggest the existence of fewer species in Rhodniini, and (iii) suggest the existence of a widespread panmictic population in the Amazon basin rather than multiple species occurring in the region. The evolution of the Rhodniini is marked by a recent diversification during the Pleistocene with climatic changes likely shaping the current distribution of the four species we validated. We emphasize the need for integrative taxonomy and genomic data in the Rhodniini tribe to resolve the conflicts detected in our study and strength vector control strategies.

## Methods

### Sampling

We collected 497 individuals from 17 species in the Rhodniini tribe (Table S1): *P. arthuri* (n = 42), *P. coreodes* Bergroth, 1911 (n = 29), *P. tertius* Lent & Jurberg (n = 24), *Rhodnius ecuadoriensis* (n = 29), *R. pallescens* (n = 55)*, R. colombiensis* (n = 14)*, R. pictipes* (n = 8)*, R. brethesi* (n = 13)*, R. stali* (n = 23)*, R. neivai* (n = 9)*, R. montenegrensis* (n = 14)*, R. milesi* (n = 17) Carcavallo, Rocha, Galv.o & Jurberg, 2001*, R. neglectus* (n = 32), *R. nasutus* (n = 9), *R. marabaensis* Souza et al., 2016 (n = 5), *R. robustus* (n = 45) and *R. prolixus* (n = 129). This sampling was conducted in seven countries (Bolivia, Brazil, Colombia, Ecuador, Panama, Peru and Venezuela) which covered the distribution of the Rhodniini tribe across Central and South America (Fig. 1, Figures S1-S4). We also collected four specimens of *Panstrongylus geniculatus* (Latreille, 1811) and two of *Triatoma dimidiata* (Latreille, 1811)*,* that were used as outgroups. All samples were collected in absolute ethanol and stored at 2–8 °C. All collected insects were identified using the taxonomic key described by Lent and Wygodzinsky^56^ and verified by expert entomologists.

### Ethics statement

This study was approved by the ethics committee of Universidad del Rosario under the permit number 007/2016 granted to the project “Genómica, evolución y biogeografía de especies del género *Rhodnius*: vectores de la enfermedad de Chagas”.

### DNA isolation, amplification, and alignment

DNA extraction was performed using the DNeasy Blood & Tissue kit (QIAGEN) with minor modifications (Appendix S1). We then amplified eight loci, seven nuclear (TRNA, PJH, CISP, LSM, UPCA, 28S, and UPMETAL) and one mitochondrial (CYTB). Most of them had been previously used by us for phylogenetic reconstructions of *Rhodnius*^21^ and *Psammolestes*^17^, except for UPMETAL (Uncharacterized Protein—Metal Ion Binding; Forward: TAGGCGGCGATGTA, Reverse: GGGCAATTCTTGTCC; 725 bp) which is included for the first time in this study. We complemented this database with sequences available for CYTB and 28S loci that were downloaded from GenBank.

PCR reactions had a final volume of 25 μL, consisting of 12.5 μL of GoTaq Green Master Mix (Promega, Madison, WI, USA), 1.25 μL (10 μM) of each primer, 5.0 μL of DNA (20 ng), and 5.0 μL of H_2_O. The following PCR cycling conditions were used: 94 °C for 5 min; 40 cycles of 94 °C for 1 min, 50–56 °C for 1 min, 72 °C for 1 min (Apppendix S1) and a final extension at 72 °C for 10 min in a Thermal Cycler 4000 (Bio-Rad Laboratories, Inc., Hercules, CA, USA). Amplification was verified on 1.5% agarose gels, and amplified products were purified using ExoSAP-IT Product Cleanup (Affymetrix, Santa Clara, CA, USA) (Appendix S1).

Bidirectional sequencing was performed using the Sanger method. Direct and reverse sequences were assembled, verified, and edited in CLC Main Workbench 20.0 (https://www.qiagenbioinformatics.com/products/clc-main-workbench/) and we obtained 2634 contigs. Alignments per each locus were performed using MAFFT (https://mafft.cbrc.jp/alignment/server/index.html), followed by visual inspection, manual correction of misalignments and determination of reading frames using Mesquite (https://mesquiteproject.org/). To resolve ambiguities, PHASE algorithm was implemented with 10,000 iterations per simulation in DnaSP v6.12.03 (http://www.ub.edu/dnasp/downloadTv6.html ).

### Molecular phylogenetic analysis and species delimitation

We generated two alignments (Table S6). First, a nuclear alignment composed by all seven nuclear loci (3,936 bp) comprising TRNA (597 bp), PJH (601 bp), CISP (522 bp), LSM (569 bp), UPCA (641 bp), UPMETAL (506 bp), and 28S (500 bp). Second, a full alignment that concatenates both nuclear and mitochondrial loci (4,368 bp). We then applied three Maximum Likelihood (ML) methods to generate phylogenetic reconstructions from these alignments. First, IQ-Tree 2 (https://www.iqtree.org/ ) was run with partitions by locus and assessing node support with traditional bootstrap (1,000 replicates), Ultrafast Bootstrap (10,000 replicates), aBayes, and SH-aLRT. We estimated locus-specific substitution models with the Bayesian Information Criterion (BIC) in ModelFinder (https://github.com/przigoda/model-finder/tree/master ). Second, we used FastTree with the GTR + CAT model (https://github.com/PavelTorgashov/FastTree) . Third, we used PhyML with the GTR + R substitution model (estimated by SMS), and node support was evaluated using traditional bootstrap (1,000 replicates) (https://github.com/stephaneguindon/phyml).

We also estimated Bayesian Inference (BI) trees for each alignment using MrBayes 3.2 (https://nbisweden.github.io/MrBayes/) and ASTRAL (https://github.com/astral-sh). Partitions were applied, and nucleotide substitution models were estimated with MrModeltest (https://github.com/nylander/MrModeltest2). Two independent runs of 15 million generations each were conducted, sampling every 1,000 generations. Convergence of the runs and effective sample size (ESS) > 200 for all parameters were assessed using Tracer v1.6 (https://github.com/beast-dev/tracer). The output files from the runs were combined using LogCombiner^59^ with the first 10% of trees discarded as burn-in. Consensus trees, including those with the highest credibility and 95% highest posterior density (HPD) intervals for nodes, were generated using TreeAnnotator. Phylogenetic trees were visualized with FigTree (http://tree.bio.ed.ac.uk/software/figtree/) and iTOL (https://github.com/TongZhou2017/itol.toolkit).

We evaluated the likelihood of the topological hypotheses derived from the full alignment (4,368 bp) using the Topology Test in IQ-Tree(http://www.iqtree.org/doc/Advanced-Tutorial). The following tests were employed in the Topology Test: RELL approximation, Kishino-Hasegawa test and weighted KS test, Shimodaira-Hasegawa test and weighted SH test, as well as expected likelihood weights and the approximately unbiased (AU) test (http://www.iqtree.org/doc/Advanced-Tutorial). We estimated a species tree with partitions, corresponding to each gene fragment, using a coalescence-based approach implemented in StarBEAST2 v2.4. These partitions were incorporated into *BEAST v2.3.2 (https://www.beast2.org/), applying both linked and unlinked tree models. The nucleotide substitution model was inferred using the bModelTest package (https://github.com/BEAST2-Dev/bModelTest).

To estimate divergence times, we first used maximum likelihood in MEGA X to estimate the molecular clock model (https://www.megasoftware.net/). We assumed Yule speciation model in *BEAST2 (https://www.beast2.org/). Calibration of the root note was performed using a uniform prior distribution set between 20.44 and 13.82 Ma derived from a fossil of *Panstrongylus hispaniolae* available in PaleoDatabase (https://paleobiodb.org). We performed four independent runs of 10,000,000 generations each, sampling every 1,000 generations. Convergence of the runs and effective sample size (ESS) > 200 for all parameters were verified using Tracer v1.6 (http://tree.bio.ed.ac.uk/software/tracer/). The output files from the four runs were combined using LogCombiner (https://beast.community/logcombiner).

TreeAnnotator (https://www.beast2.org/treeannotator/) was used to discard the initial 10% of trees as burn-in and to generate consensus trees with the highest credibility and 95% highest posterior density (HPD) intervals for each node. The 95% confidence intervals for estimated divergence dates were calculated from the posterior distribution of ages for each clade in TreeAnnotator (https://www.beast2.org/treeannotator).

We used a joint Bayesian inference approach for species delimitation using iBPP (https://github.com/cecileane/iBPP). In each analysis, we used the species-tree topology generated by PhyML as the guide tree. The molecular matrix included all sequences available for the markers. We set nine combinations of prior distributions for the ancestral population size (θ) and the root age of the tree (τ), ranging from scenarios with large population sizes and deep divergence times (θ = G(1,10) and τ = G(1,10)) to those with small population sizes and shallow divergence times (θ = G(2,2000) and τ = G(2,2000))^64^.

### Population genetics analysis

We characterized genetic variability at four levels: (i) within species in the tribe, (ii) between genus in the tribe (*Psammolestes* and *Rhodnius*), (iii) between the three groups within the *Rhodnius* genus (*prolixus*, *pallescens* and *pictipes*), (iv) *prolixus* clades : *prolixus* 1 (*R. neglectus*, *R. nasutus*, *R. milesi*) and *prolixus* 2 (*R. prolixus*/*R. robustus*/*R. marabaensis*/*R. montenegrensis*)^39^. For this we estimated genetic diversity measures for all loci using DNASP v6.12.03: haplotype diversity (h), number of segregating sites (S), population substitution rate (θ), nucleotide diversity (π), and three neutrality tests—Tajima’s D (D), Tajima’s F, and the D statistics by Fu & Li. We evaluated shared ancestry and clustering with STRUCTURE v2.3 using the nuclear alignment, applying 100,000 Markov Chain Monte Carlo (MCMC) generations, and sampling K values from 1 to 20 with 5 iterations per K. The optimal K value was determined using STRUCTURE HARVESTER (https://github.com/dentearl/structureHarvester) and visualized with pophelper (http://pophelper.com/). Finally, we evaluated genetic structure among the 17 species in the tribe, as well as among *Psammolestes* and the groups within the *Rhodnius* genus (*prolixus*, *pallescens* and *pictipes*), by estimating the fixation index (F_ST_) and two absolute measures (Da, Dxy). We applied the Hudson permutation *t*-test with 1,000 replicates to assess deviations from panmixia for F_ST_ in DNASP v6.12.03 (http://www.ub.edu/dnasp/downloadTv6.html).

## Supplementary Information

Supplementary Information.

## Data Availability

Sequence data that support the findings of this study have been deposited in Genbank under the accession numbers PQ605815 -PQ606053 and PQ585846-PQ586084.
