# Supplementary material for: Multilocus analysis uncovers the evolution of the Rhodniini tribe, vectors of Trypanosoma cruzi
Source: Sci Rep. 2025 Jul 1;15:20401. doi: 10.1038/s41598-025-03789-9 (PMC12218322; doi:10.1038/s41598-025-03789-9)
Supplement: Supplementary file 1 — Supplementary Information. [file 41598_2025_3789_MOESM1_ESM.pdf]

Appendix S1. DNA extraction and PCR

1. **DNA extraction protocol used in this study:** DNeasy® Blood & Tissue Qiagen kit protocol with modifications

2. Dissect thorax and legs of each insect and put in Eppendorf tubes.
3. Wash 3 times with Water PCR-grade by centrifugation for 1 min (2000 rpm).
4. Add 360µl Buffer ATL and 40 µl proteinase K. Mix gently by hand shaking.
5. Incubate at 56°C for 12 hours.
6. Add 400 µl Buffer AL to the sample. Mix gently by hand shaking.
7. Add 400 µl ethanol (96–100%) and mix again by hand shaking.
8. Pipet the mixture from step 5 into the DNeasy Mini spin column placed in a 2 ml collection tube (provided). Centrifuge at (8000 rpm) for 1 min. Discard flow-through and collection tube.
9. Place the DNeasy Mini spin column in a new 2 ml collection tube (provided), add 500 µl Buffer AW1, and centrifuge for 1 min at 8000 rpm. Discard flow-through and collection tube.
10. Place the DNeasy Mini spin column in a new 2 ml collection tube, add 500 µl Buffer AW2, and centrifuge for 3 min at 14,000 rpm to dry the DNeasy membrane. Discard flow-through and collection tube.
11. Place the DNeasy Mini spin column in a clean 1.5 ml or 2 ml microcentrifuge tube, and pipet 150 µl Buffer AE directly onto the DNeasy membrane. Incubate at room temperature for 1 min, and then centrifuge for 1 min at 8000 rpm) to elute.

Notes: The ends of pippetes tips used in the protocol were cut.

Finally, we analyzed the concentration of extracted DNA with nanodrop (Table 1) and Quibit and verified the integrity on agarose gel (Figure 1.)

ID	DNA yield (100 µ L of DNA)	DNA Purity 260/280
**1**	0.7 ug	2.0
**2**	**1.7 ug**	**1.9**
**3**	2.5 ug	1.3
**4**	1.9 ug	1.4
**5**	1.0 ug	1.3
**6**	1.1 ug	1.3
**7**	1.0 ug	1.3
**8**	1.5 ug	1.6
**9**	1.5 ug	1.6
**10**	**0.95 ug**	**1.8**
**11**	**1.7 ug**	**1.9**
**12**	**0.9**	**2.0**

Figure 1. M: 1 Kb DNA ladder **1-10, R9-1, R9-2:** *R. prolixus* DNA**.** Electrophoresis on a 2% agarose gel visualized by staining with Gel Red 100-bp weight marker.

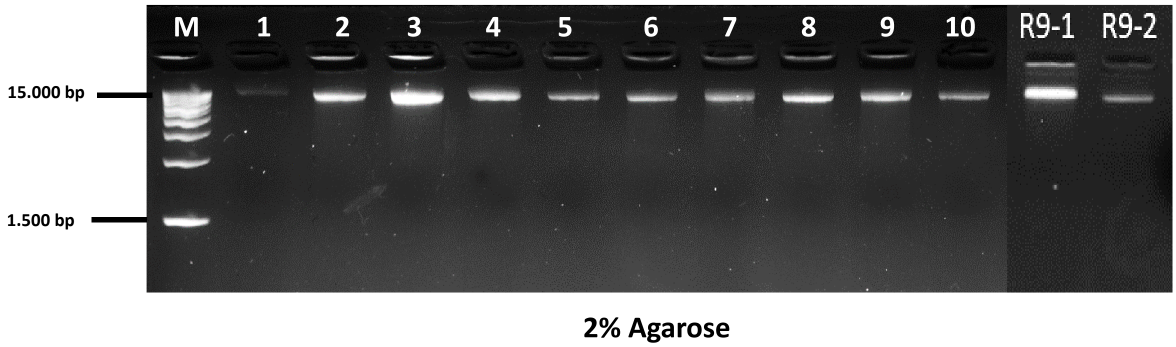

2. PCR Conditions used in this study

PCR reactions had a final volume of 25 µl, consisting of 12.5 µl of GoTaq Green Master Mix (Promega, Madison, WI, USA), 1.25 µL (10 µM) of each primer and, 5.0 µl of DNA (20 ng) and 5µL of H_2O. Amplification was conducted in a Thermal Cycler 4000 (Bio-Rad La-boratories, Inc., Hercules, CA, USA). The following PCR cycling conditions were used: 94°C for 5 min; 40 cycles of 94°C for 1 min, 50–56°C for 1 min (Table 2), and a final extension at 72°C for 10 min.

Figure 2. tRNA (Guanina (37) -N (1) metiltransferasa (842 bp).

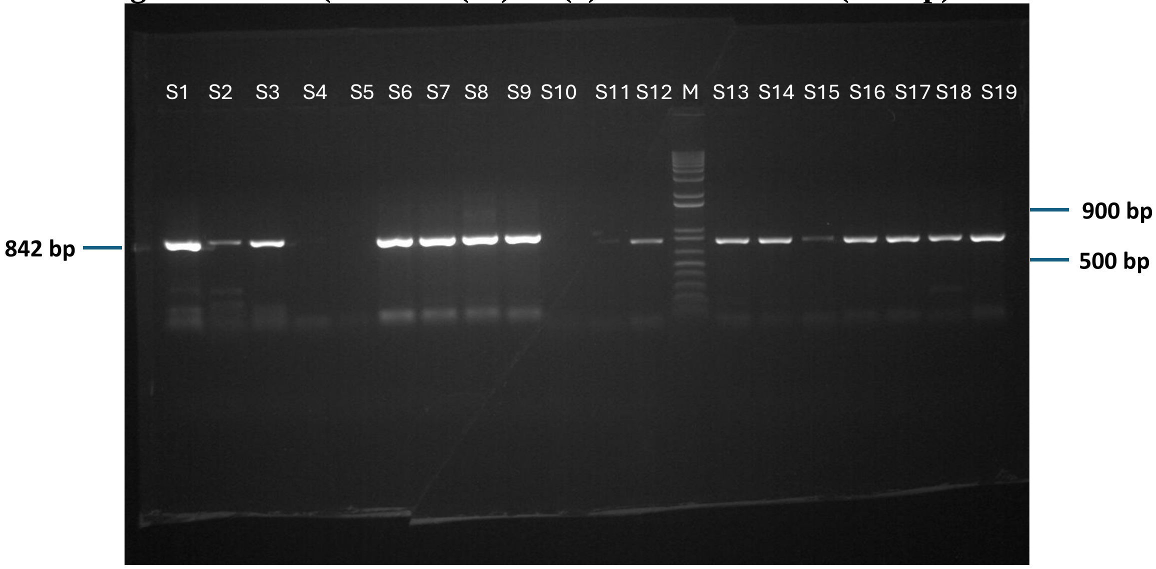

M: 1Kb DNA ladder **S1-S19:** *R. prolixus* DNA**.** Electrophoresis on a 1% agarose gel visualized by staining with Gel Red 100-bp weight marker.

Figure 3. Putative juvenile hormone inducible protein (720 bp)

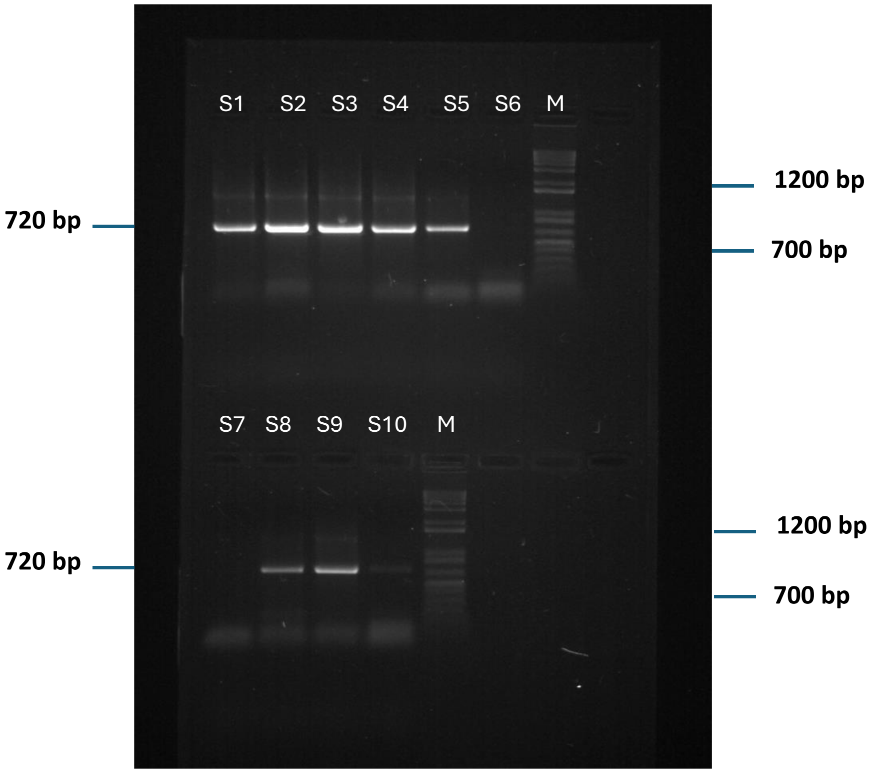

M: 1Kb DNA ladder **S1-S3:** *R. prolixus* DNA on a 1% agarose gel visualized by staining with Gel Red 100-bp weight marker.

Figure 4. Probable cytosolic iron sulfur protein assembly protein Ciao 1 (706 bp)

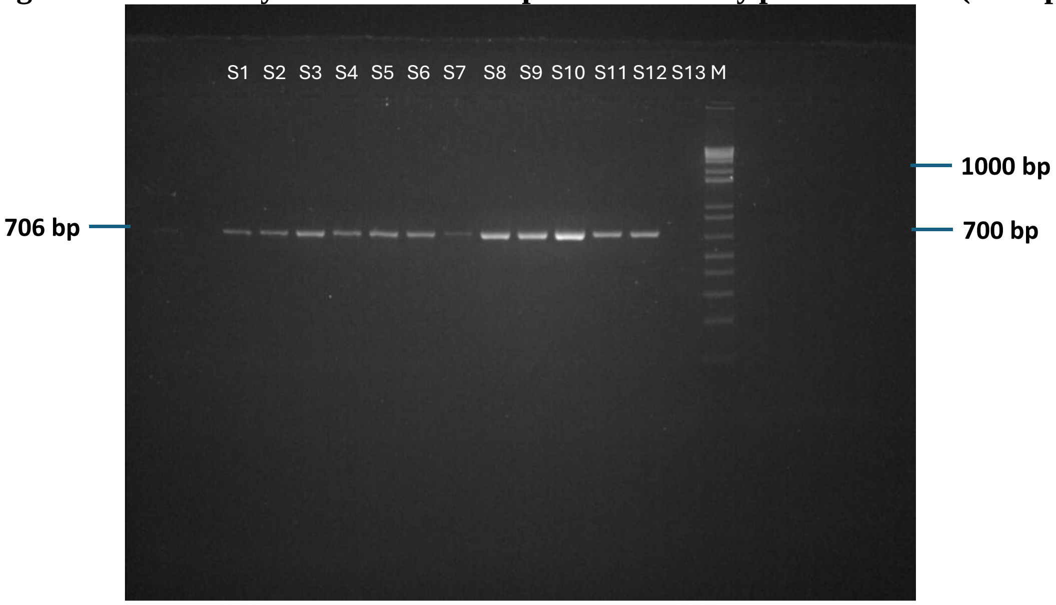

M: 1Kb DNA ladder **S1-S2:** *R. prolixus* DNA**.** Electrophoresis on a 1% agarose gel visualized by staining with Gel Red 100-bp weight marker.

Figure 5. Lipoyl synthase mitocondrial (768 bp)

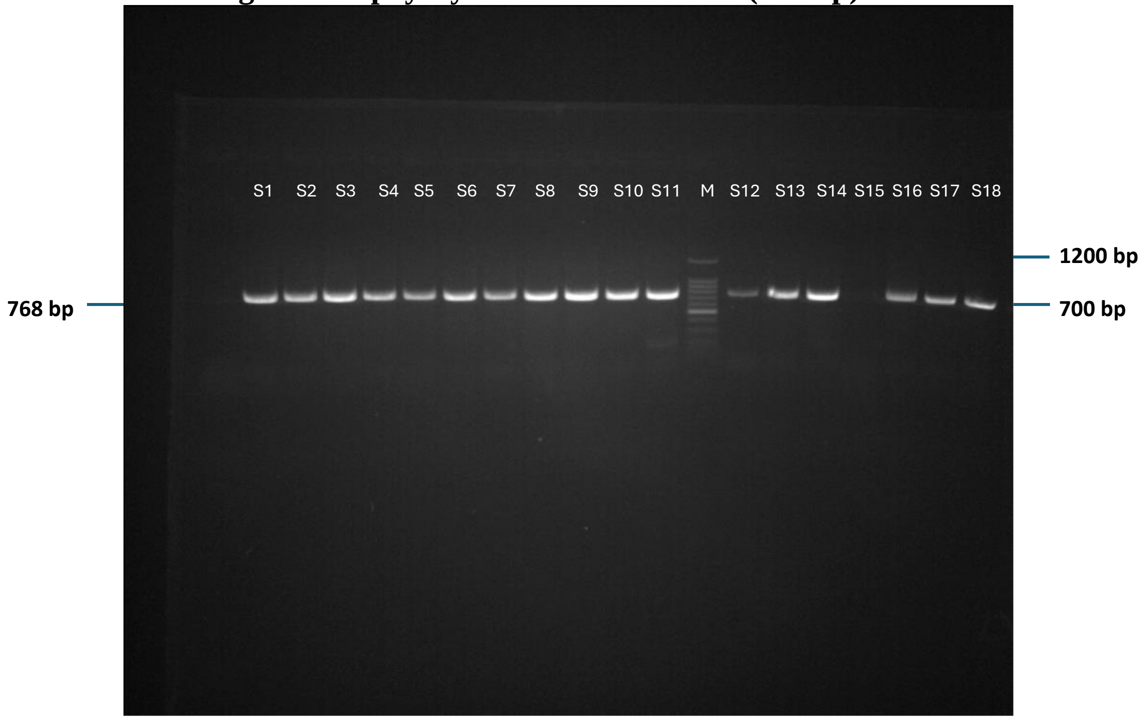

M: 1Kb DNA ladder **S1-S2:** *R. prolixus* DNA**.** Electrophoresis on a 1% agarose gel visualized by staining with Gel Red 100-bp weight marker.

Figure 6. Uncharacterized protein Cell adhesion (795 bp)

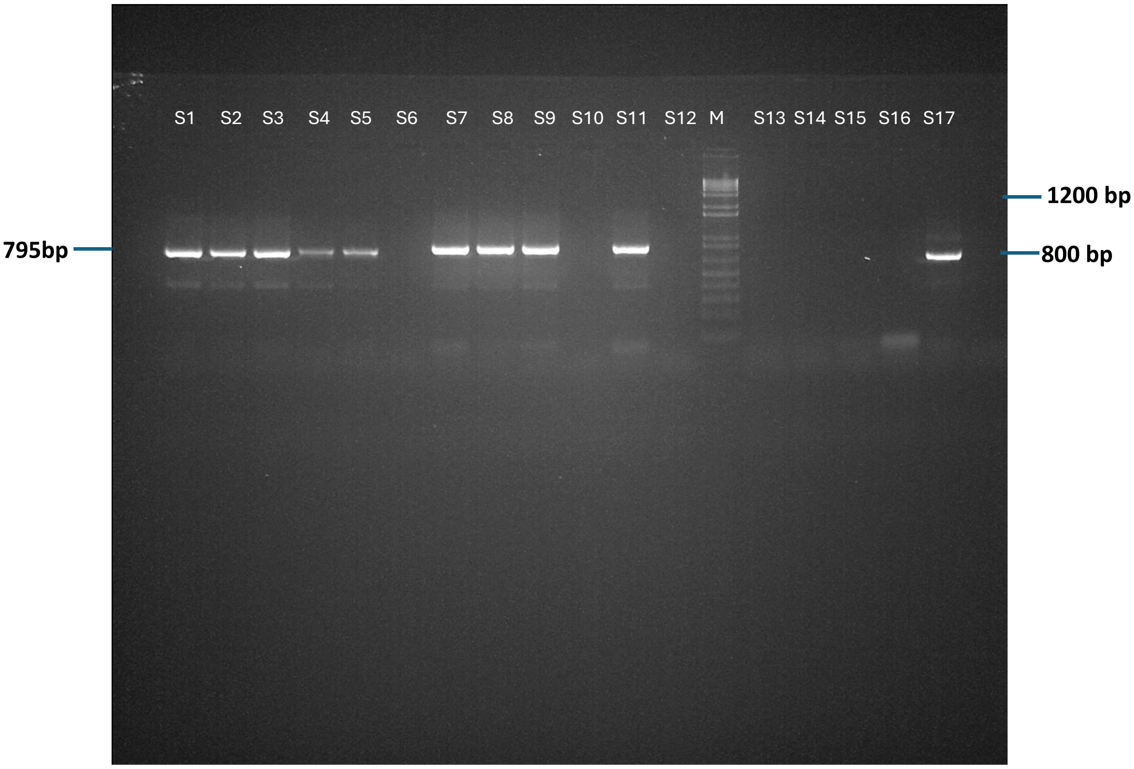

M: 1Kb DNA ladder **S1-S2:** *R. prolixus* DNA**.** Electrophoresis on a 1% agarose gel visualized by staining with Gel Red 100-bp weight marker.

Figure 7. Uncharacterized protein- metal ion binding (725 bp)

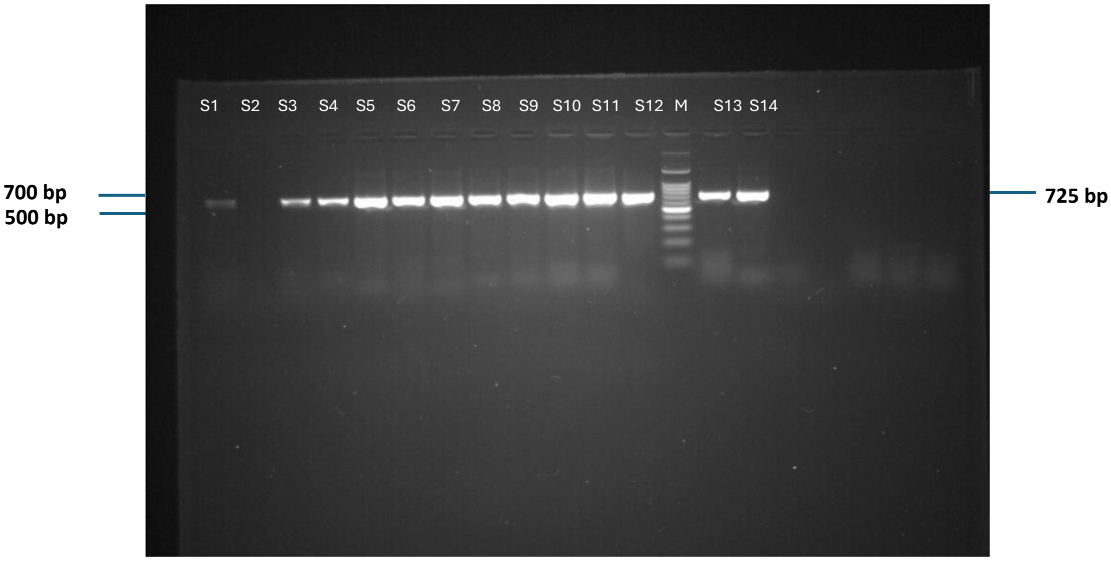

M: 500bp DNA ladder **S1-S3**: *R. prolixus* DNA. Electrophoresis on a 1% agarose gel visualized by staining with Gel Red 100-bp weight marker.

Figure 8. Cytochrome b (840 bp)

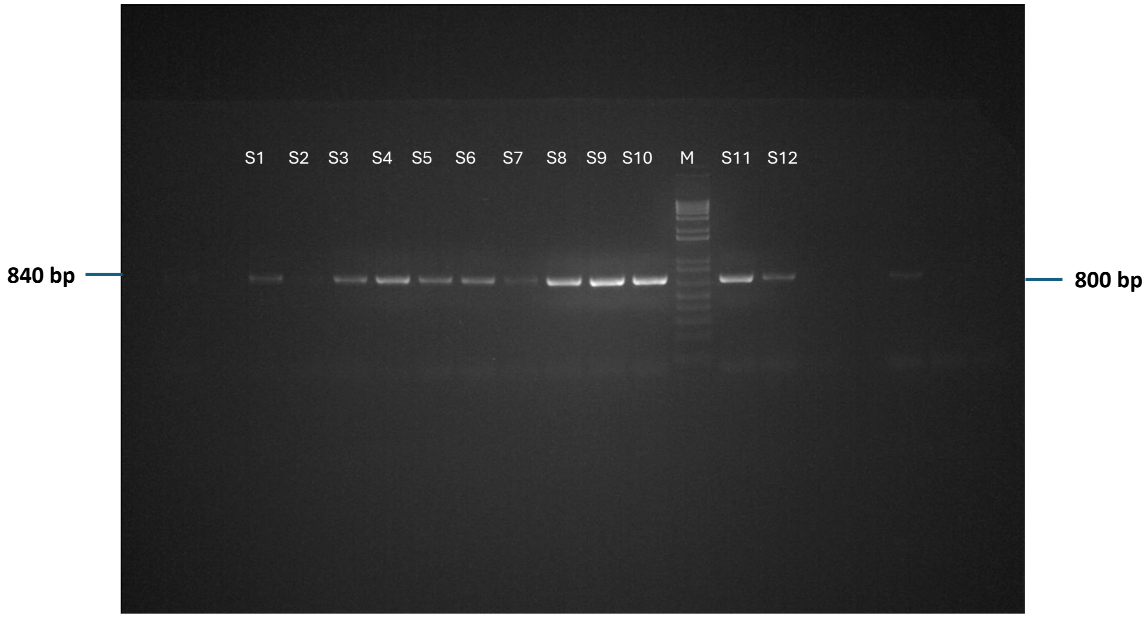

M: 1kb DNA ladder **S1-S6**: *R. prolixus* DNA **NC**: Negative Control Electrophoresis on a 1% agarose gel visualized by staining with Gel Red 100-bp weight

Figure 9.28S rRNA (696 bp)

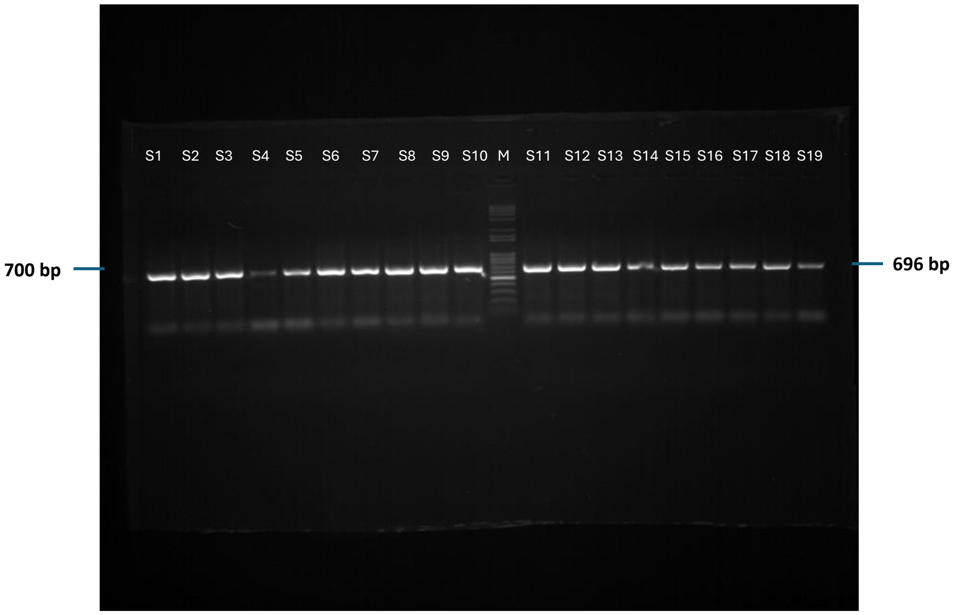

M: 1kb DNA ladder **S1-S4**: *R. prolixus* DNA **NC**: Negative Control. Electrophoresis on a 1% agarose gel visualized by staining with Gel Red 100-bp weight

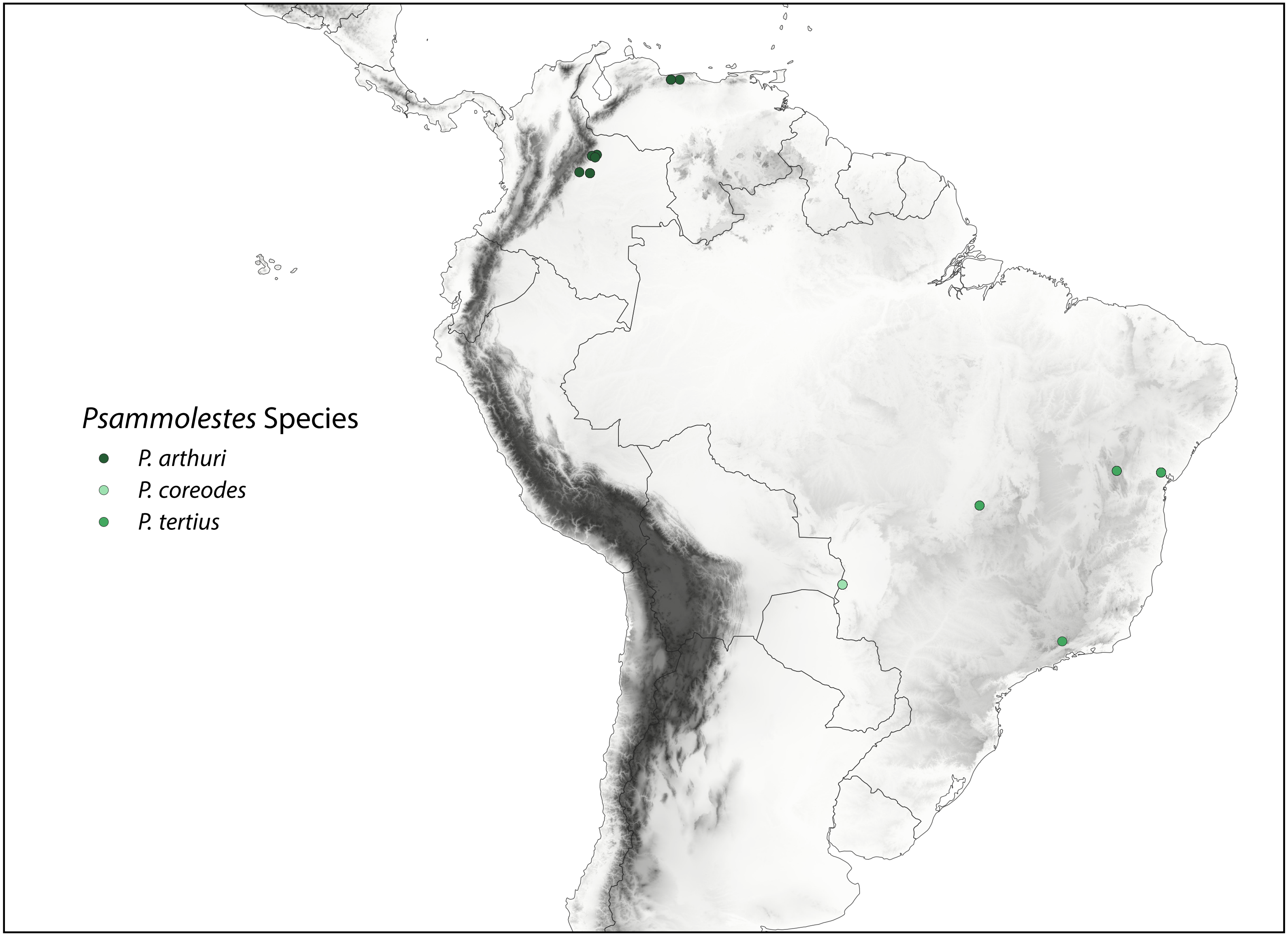

Supplementary Figure 1. Geographical distribution of Psammolestes species included in this study. The map shows the distribution of all three species of the Psammolestes genus, with dots indicating the sampling loca-tions.

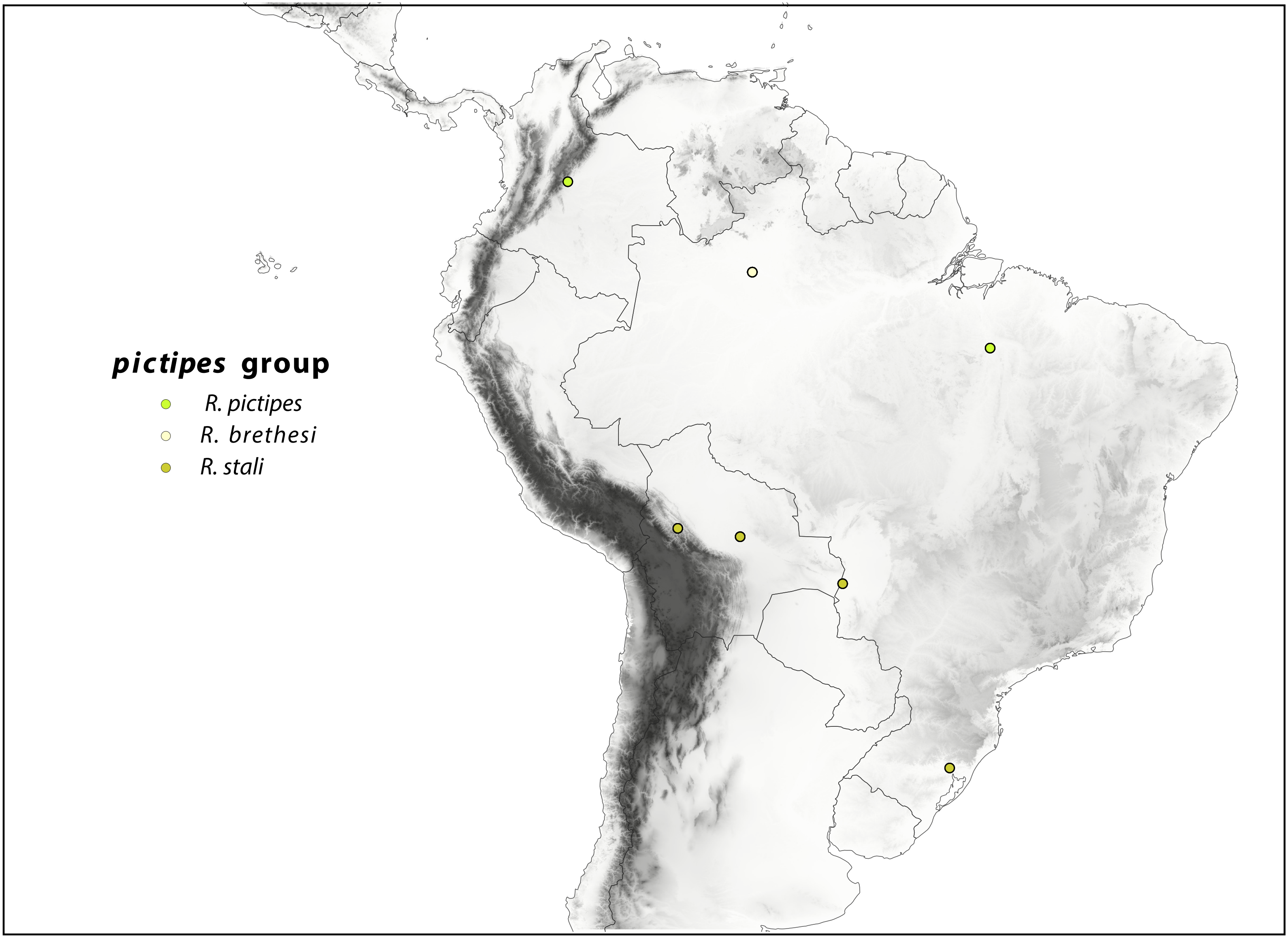

Supplementary Figure 2. Geographical distribution of *pictipes* group species included in this study. The map illustrates the distribution of the three species of the *pictipes* group, with dots indicating the sampling locations.

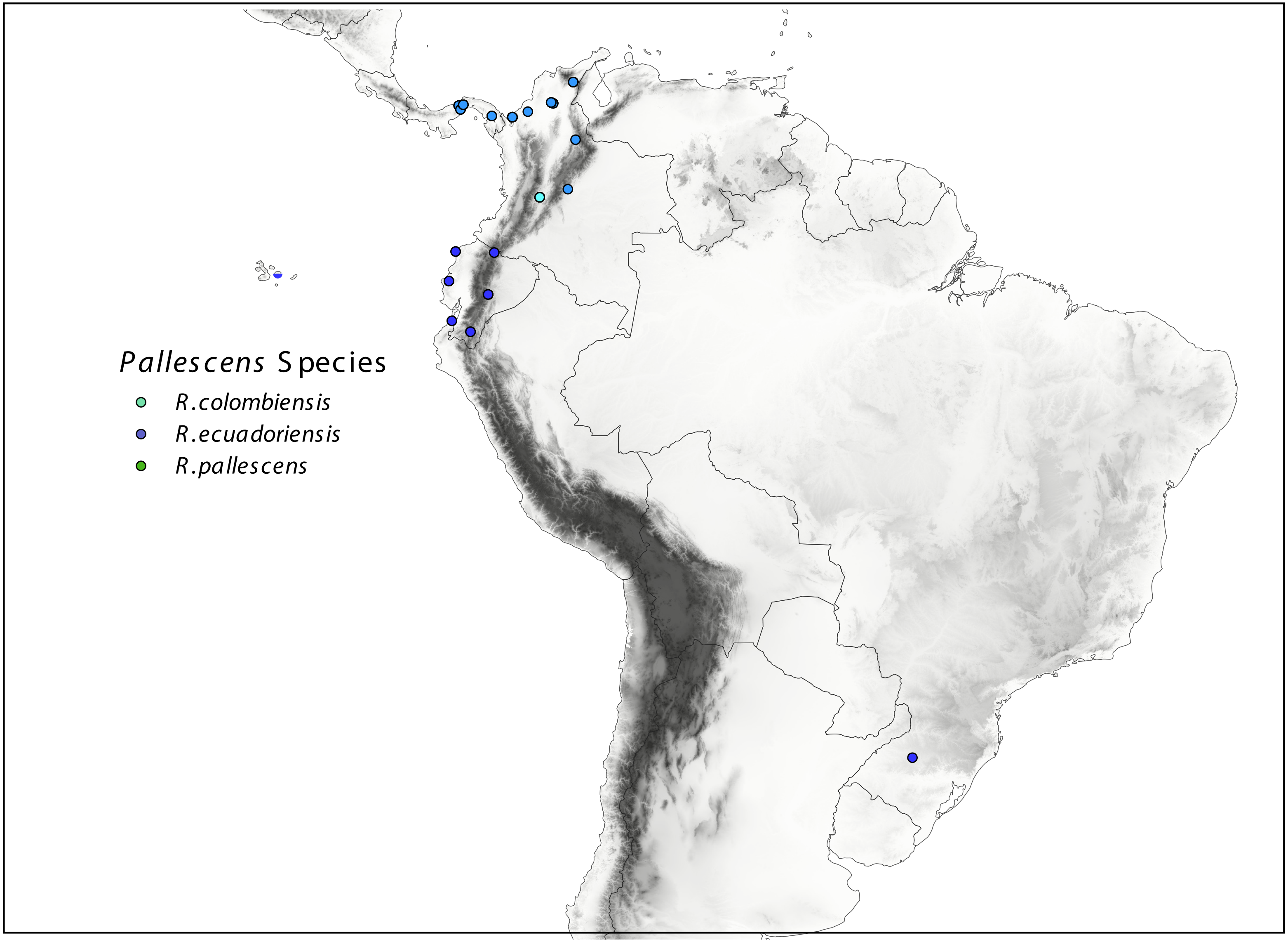

Supplementary Figure 3. Geographical distribution of *pallescens* group species included in this study. The map illustrates the distribution of the three species of the *pallescens* group, with dots indicating the sampling locations.

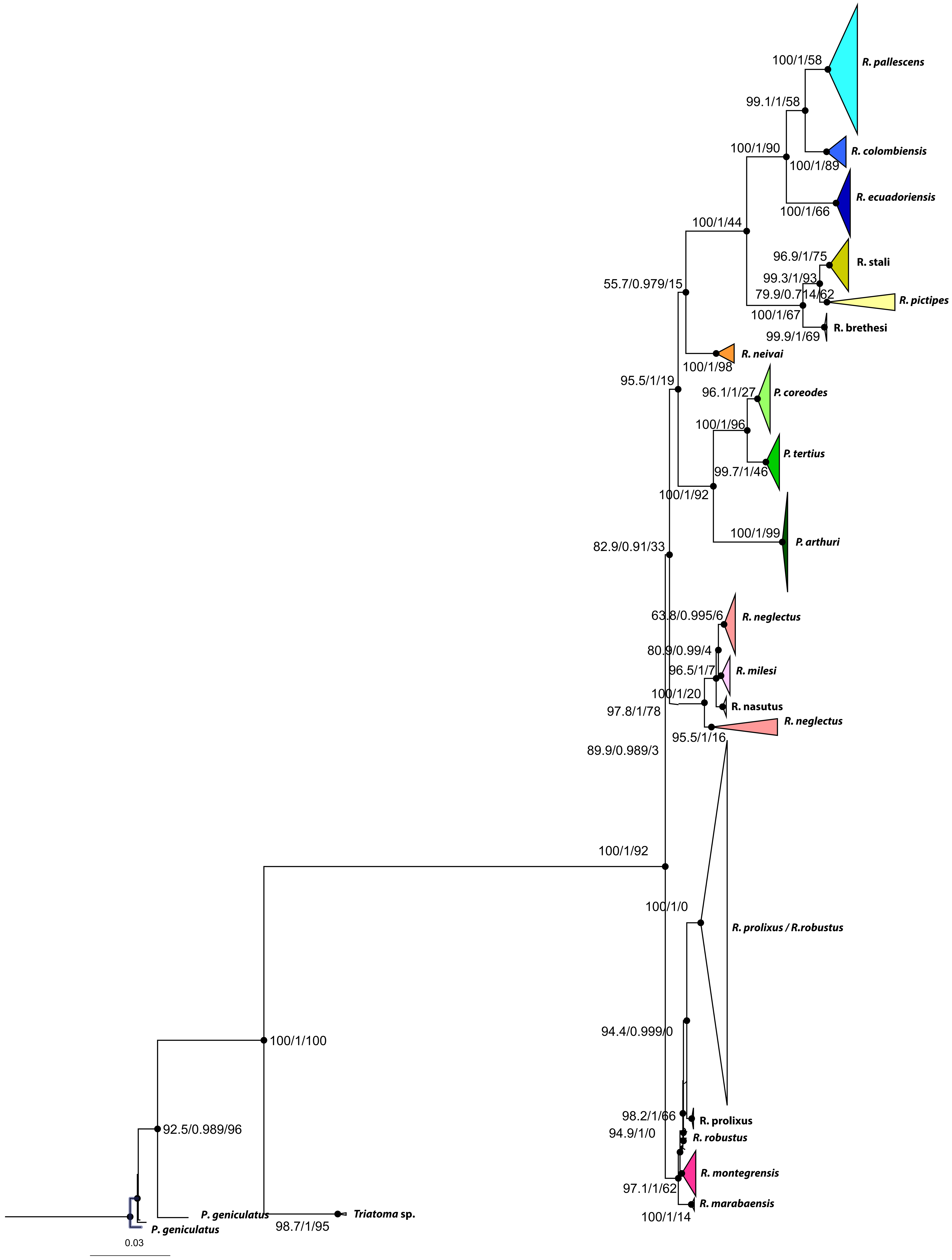

Supplementary Figure 5. Phylogenetic reconstruction of the Rhodniini tribe using ML generated with IQTree. This figure shows the phylogenetic reconstruction of the Rhodniini tribe based on a concatenated alignment (size = 4436 bp) of the eight loci used in this study (nuclear, ribosomal, and mitochondrial). The reconstruction was performed using the Maximum Likelihood algorithm with IQTree. Bootstrap values on the internal nodes are shown in the following order: SH-aLRT/aBayes/ultrafast bootstrap support.

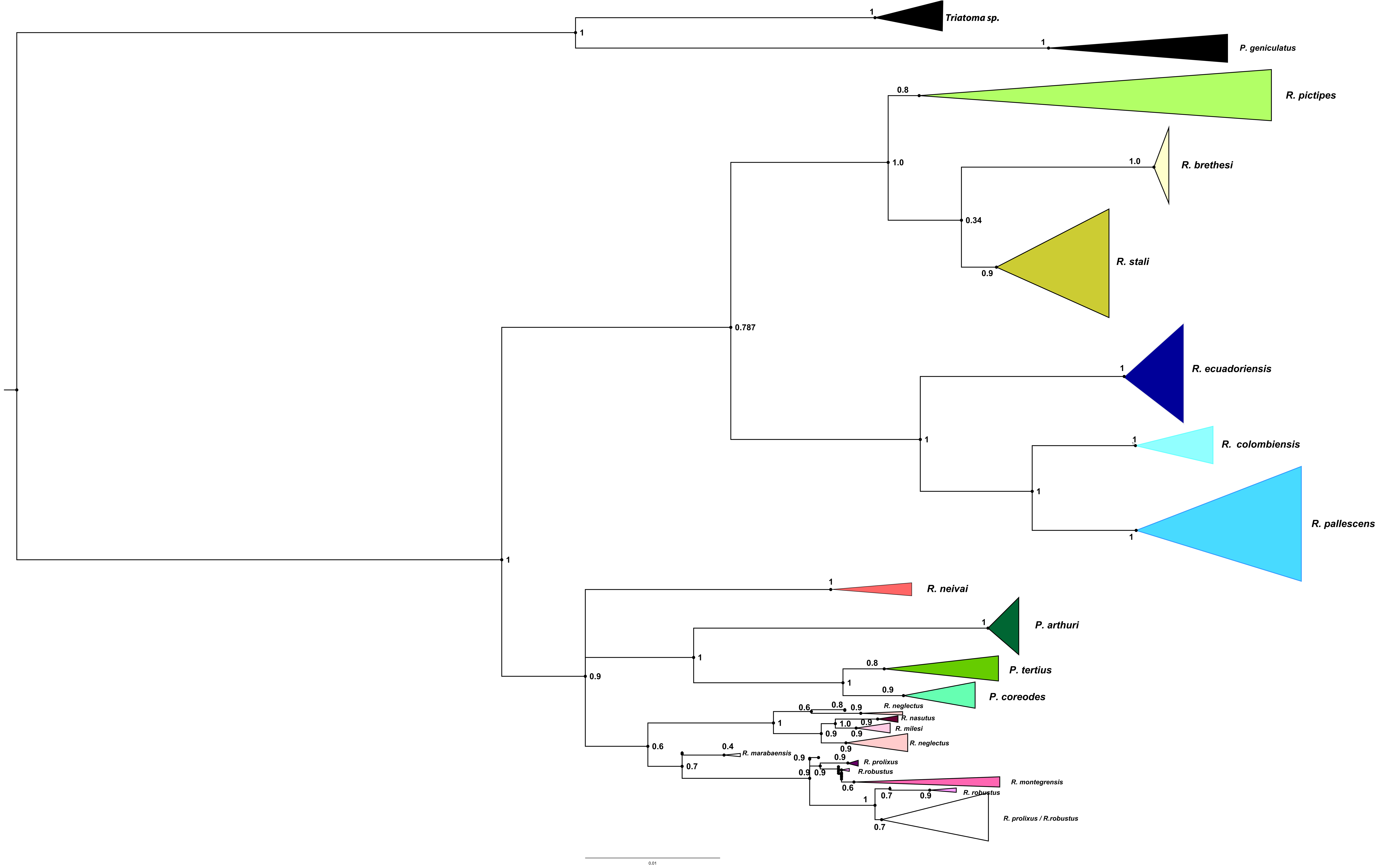

Supplementary Figure 6. Phylogenetic reconstruction of the Rhodniini tribe using ML generated with Fast Tree. This figure shows the phylogenetic reconstruction of the Rhodniini tribe based on a concatenated alignment (size = 4436 bp) of the eight loci used in this study (nuclear, ribosomal, and mitochondrial). The reconstruction was performed using the Maximum Likelihood algorithm in FastTree. Bootstrap values on the internal nodes are shown.

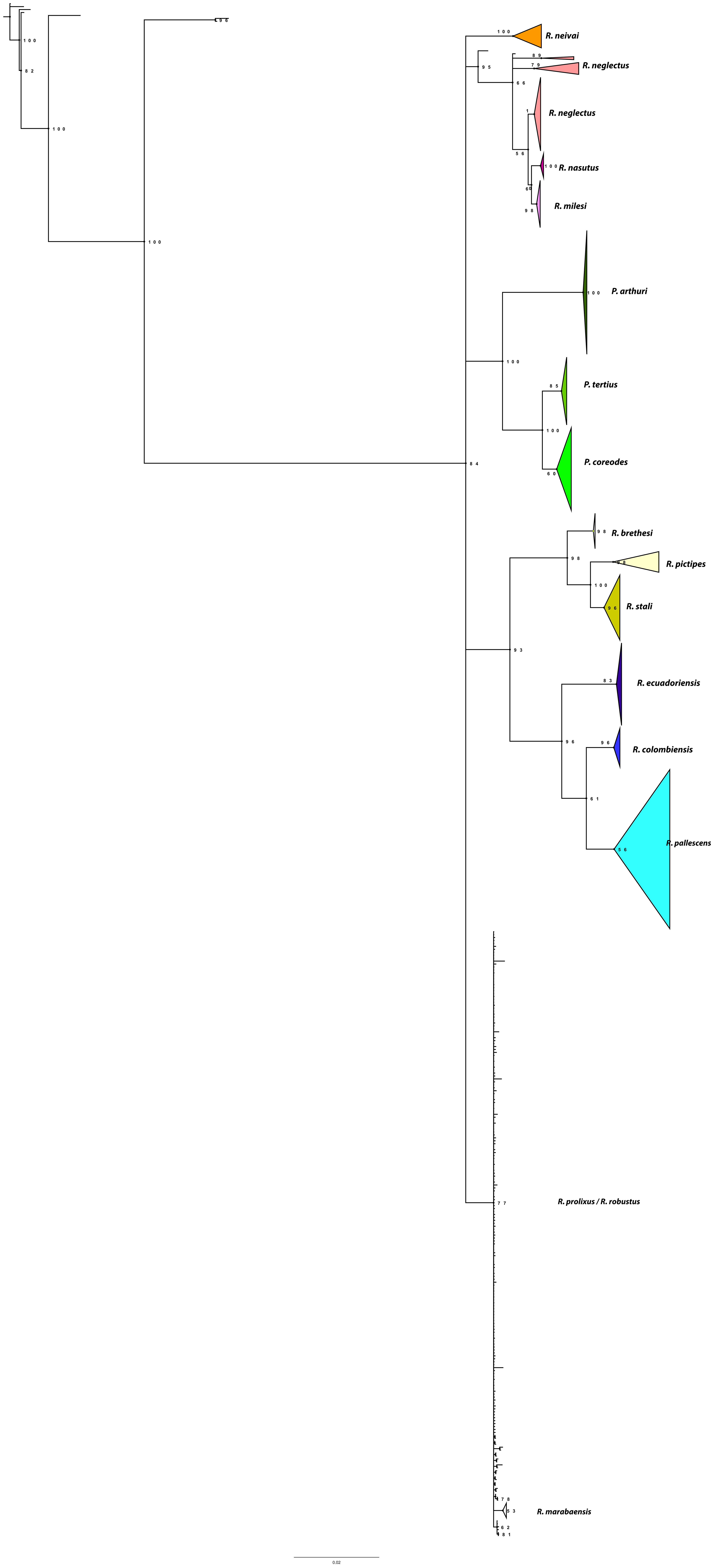

Supplementary Figure 7. Bayesian inference phylogenetic tree of the Rhodniini tribe using MrBayes 3.2. This figure shows the phylogenetic reconstruction of the Rhodniini tribe based on a concatenated alignment (size = 4436 bp of the eight loci used in this study (nuclear, ribosomal, and mitochondrial). The reconstruction was performed using Bayesian Inference algorithm with MrBayes 3.2. Posterior probability values of internal nodes are shown.

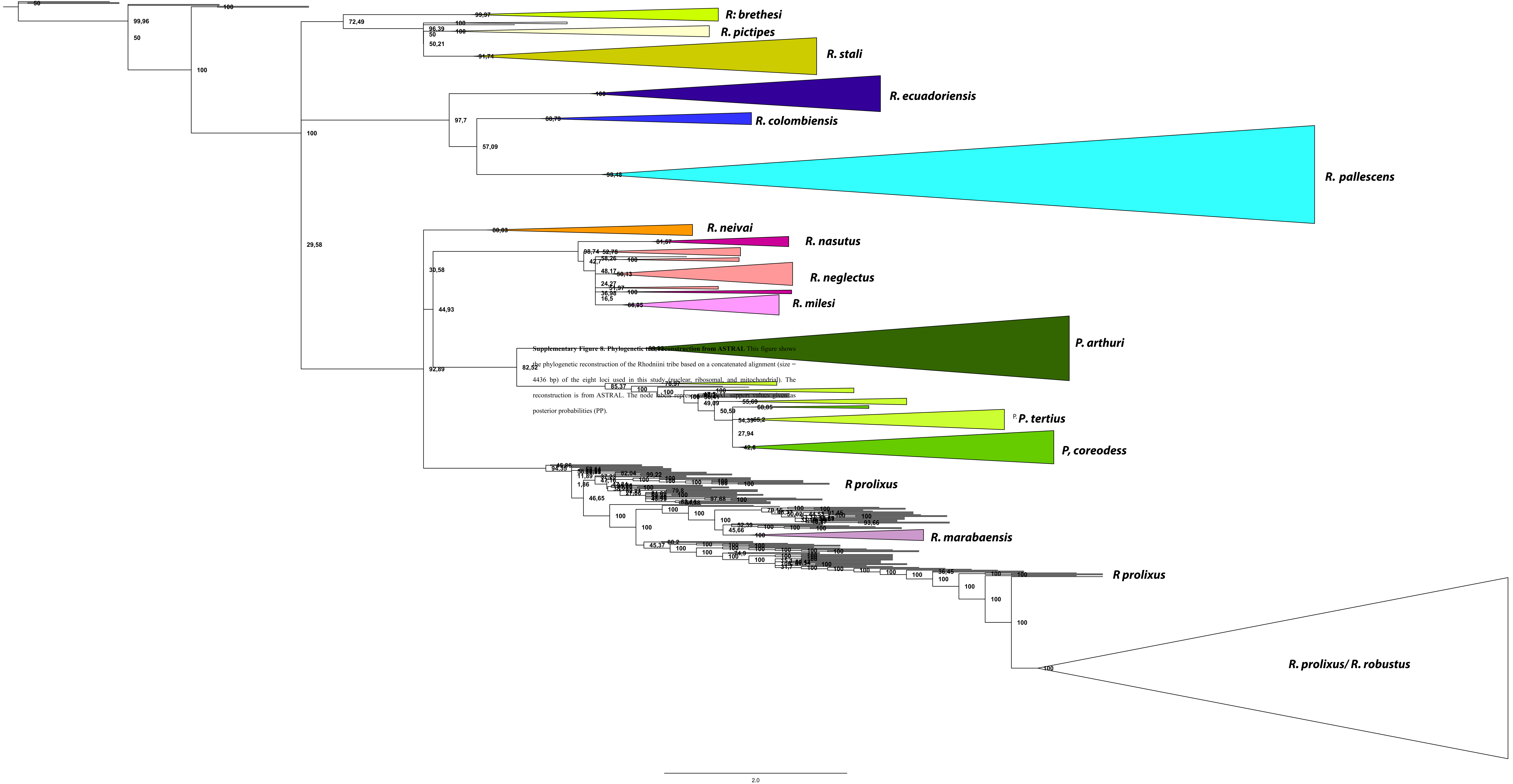

Supplementary Figure 8. Phylogenetic tree reconstruction from ASTRAL This figure shows the phylogenetic reconstruction of the Rhodniini tribe based on a concatenated alignment (size = 4436 bp) of the eight loci used in this study (nuclear, ribosomal, and mitochondrial). The reconstruction is from ASTRAL. The node labels represent ASTRAL support values given as posterior probabilities (PP).

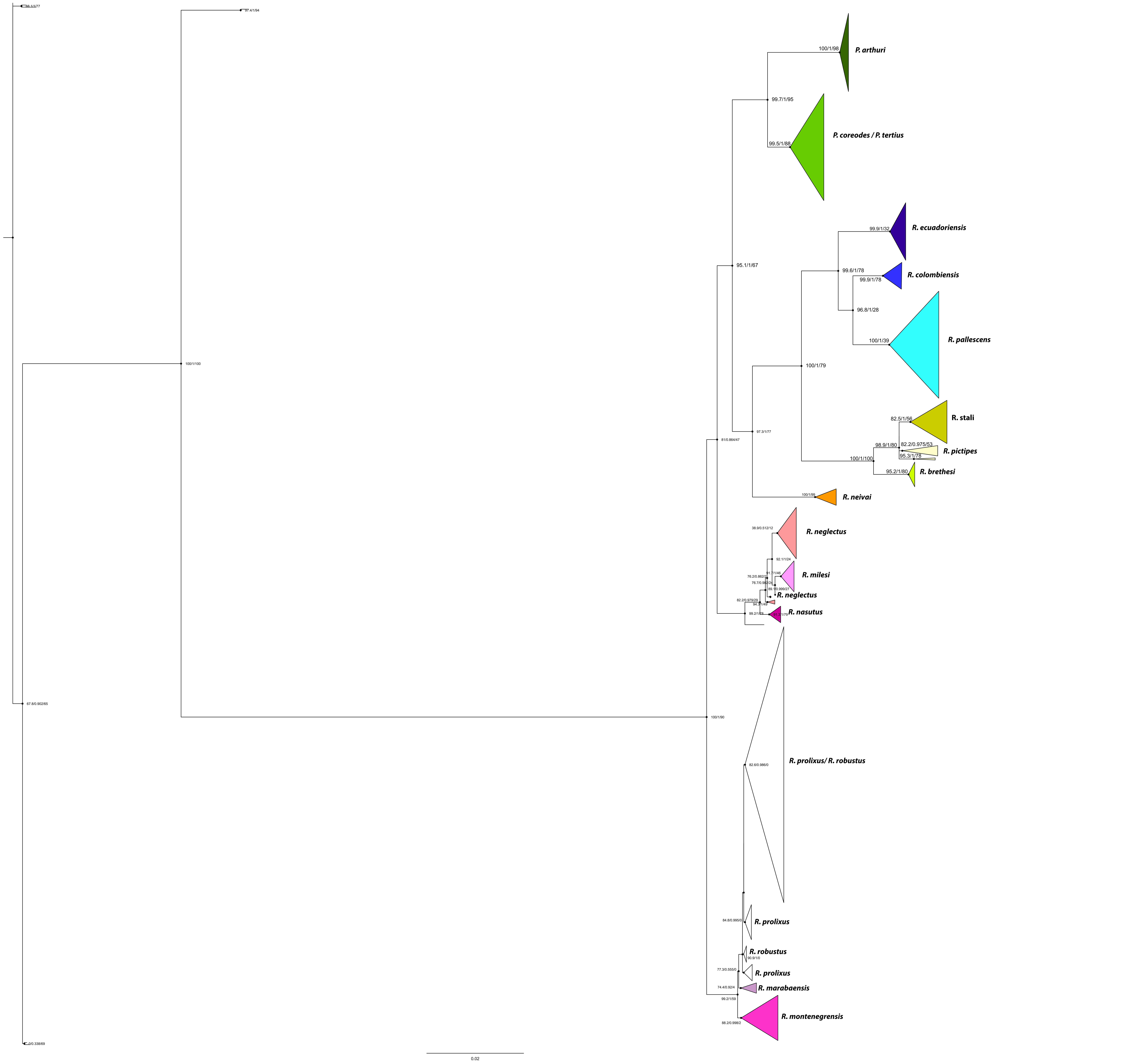

Supplementary Figure 9. Nuclear Loci Phylogenetic reconstruction of the Rhodniini tribe using ML generated with IQTree. This figure shows the phylogenetic reconstruction of the Rhodniini tribe based on a concatenated alignment (size = 3,936 bp) of the seven loci used in this study (nuclear and ribosomal). The reconstruction was performed using the Maximum Likelihood algorithm with IQTree. Bootstrap values on the internal nodes are shown in the following order: SH-aLRT/aBayes/ultrafast bootstrap support.

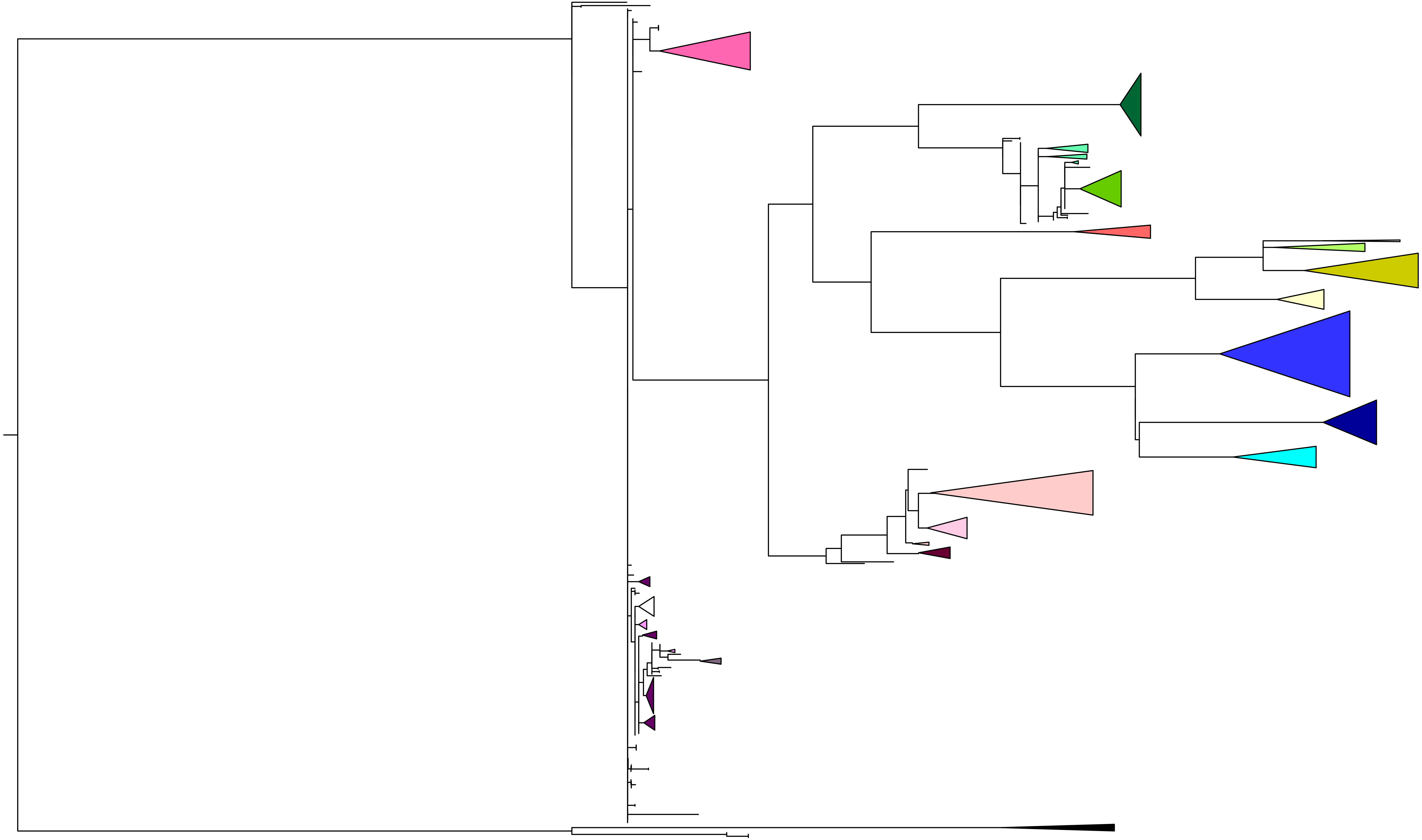

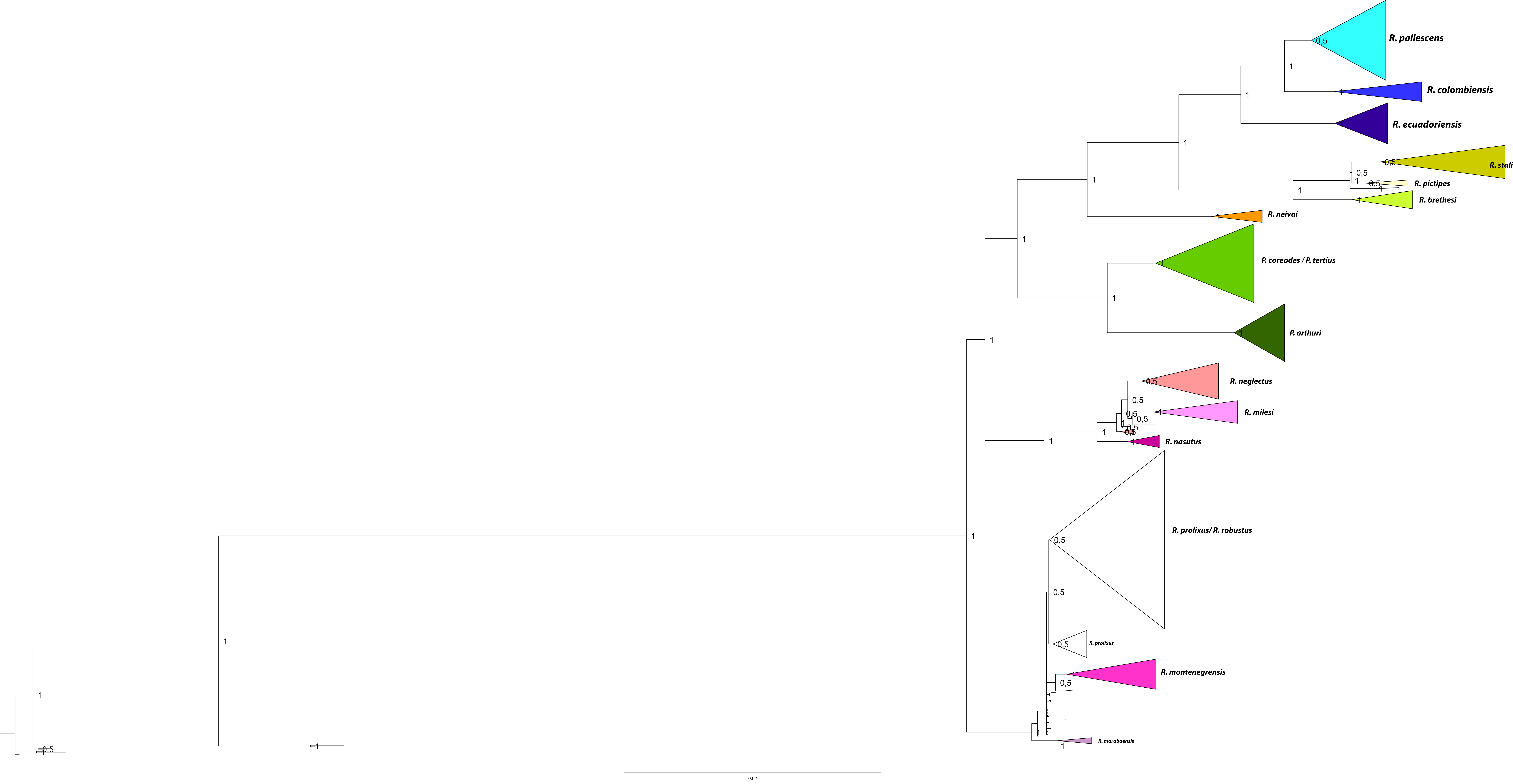

Supplementary Figure 11. Nuclear Loci Bayesian inference phylogenetic tree of the Rhodniini tribe using MrBayes 3.2. This figure shows the phylogenetic reconstruction of the Rhodniini tribe based on a concatenated alignment (size = 3,936 bp) of the seven loci used in this study (nuclear and ribosomal). The reconstruction was performed using Bayesian Inference algorithm with MrBayes 3.2. Posterior probability values of internal nodes are shown.

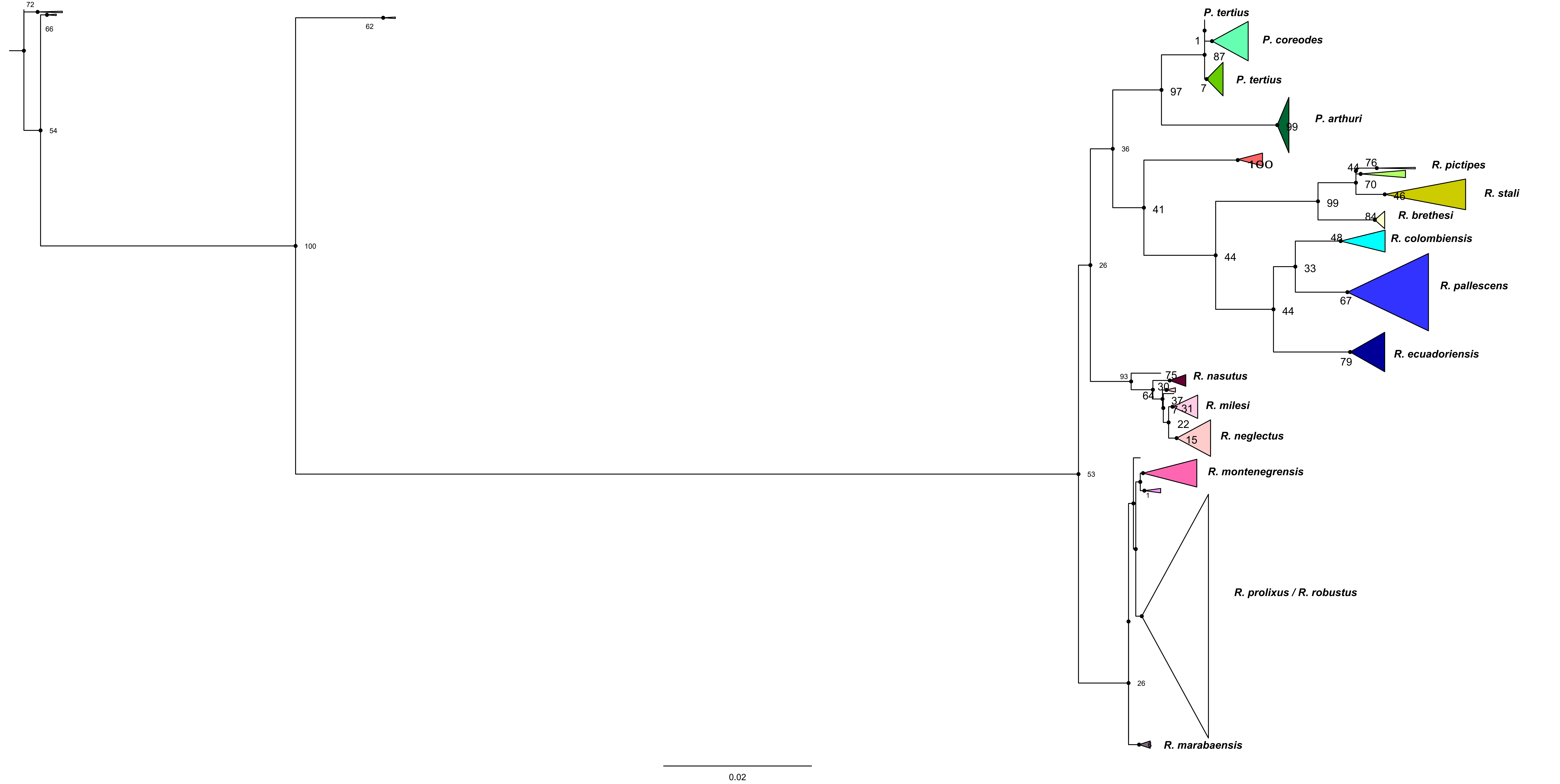

Supplementary Figure 12. Nuclear Loci Phylogenetic reconstruction of the Rhodniini tribe using ML generated with PHYML. This figure shows the phylogenetic reconstruction of the Rhodniini tribe based on a concatenated alignment (size = 3,936 bp) of the seven loci used in this study (nuclear and ribosomal). The reconstruction was performed using th Maximum Likelihood algorithm with PHYML and 1000 bootstrap repetitions. Bootstrap values are indicated in the nodes.

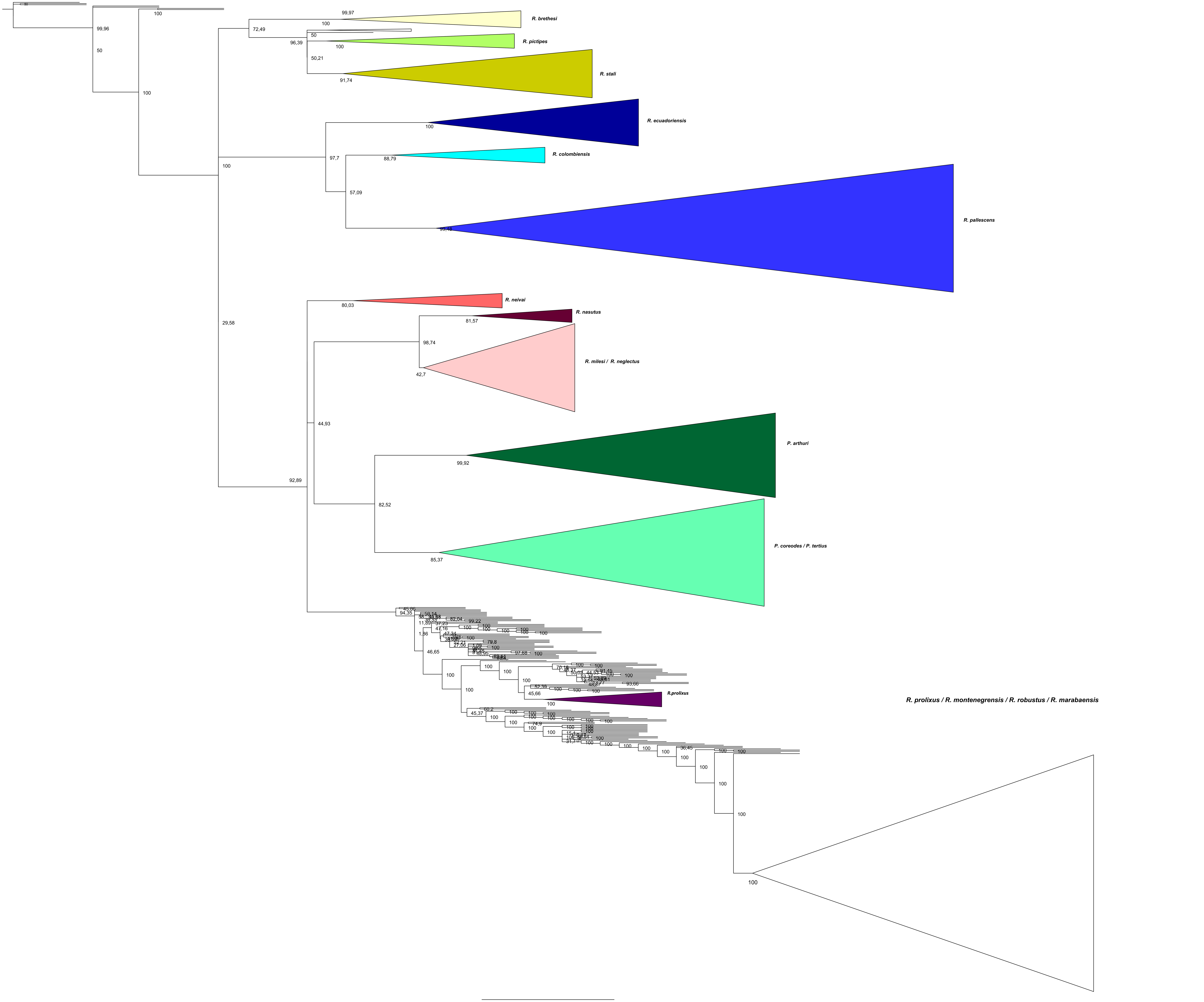

Supplementary Figure 13. Phylogenetic tree reconstruction from ASTRAL. This figure shows the phylogenetic reconstruction of the Rhodniini tribe based on a concatenated alignment (size = 3,936 bp) of the seven loci used in this study (nuclear and ribosomal). The reconstruction is from ASTRAL . The node labels represent ASTRAL support values given as posterior probabilities (PP).

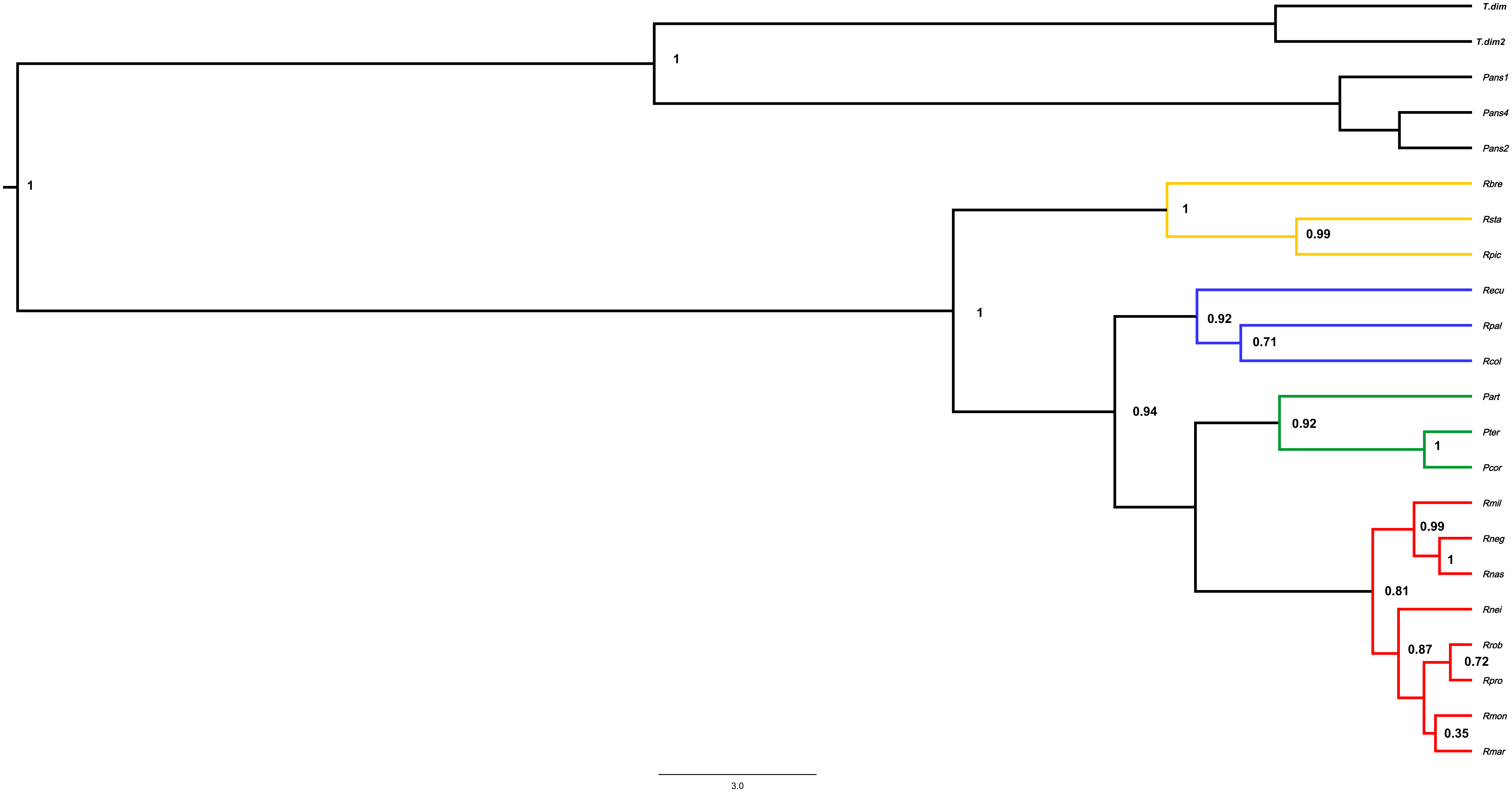

Supplementary Figure 14. Nuclear loci Species tree estimation. Species tree inferred from Bayesian analysis of the seven nuclear genes examined in this study using StartBEAST2. Posterior probabilities are showed in the nodes.

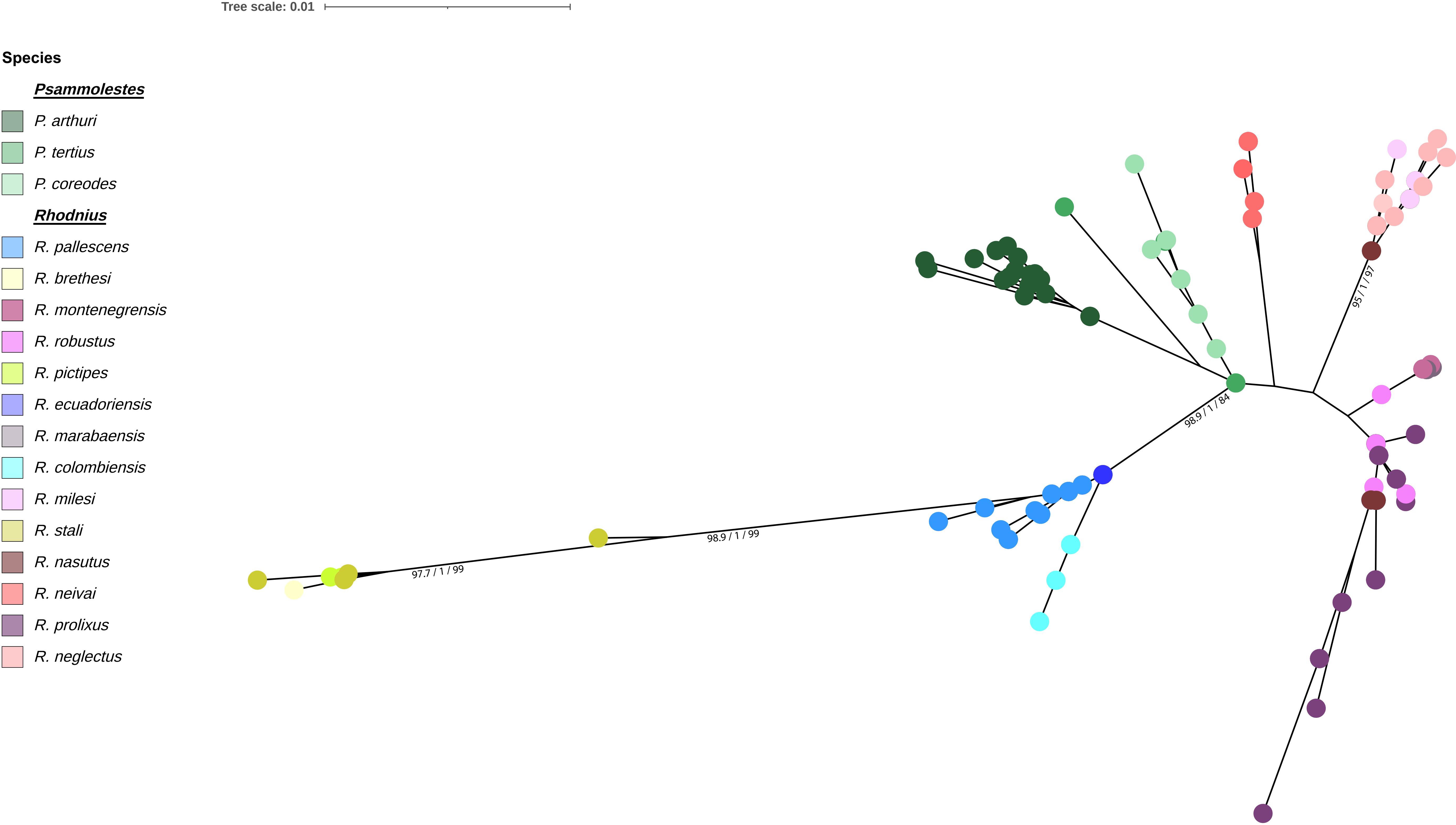

Supplementary Figure 15. 28S Phylogenetic reconstruction. Phylogenetic reconstruction with the ML algorithm based on the nuclear marker 28S. The reconstruction was performed using the Maximum Likelihood algorithm with IQTree. Bootstrap values on the internal nodes are shown in the following order: SH-aLRT/aBayes/ultrafast bootstrap support.

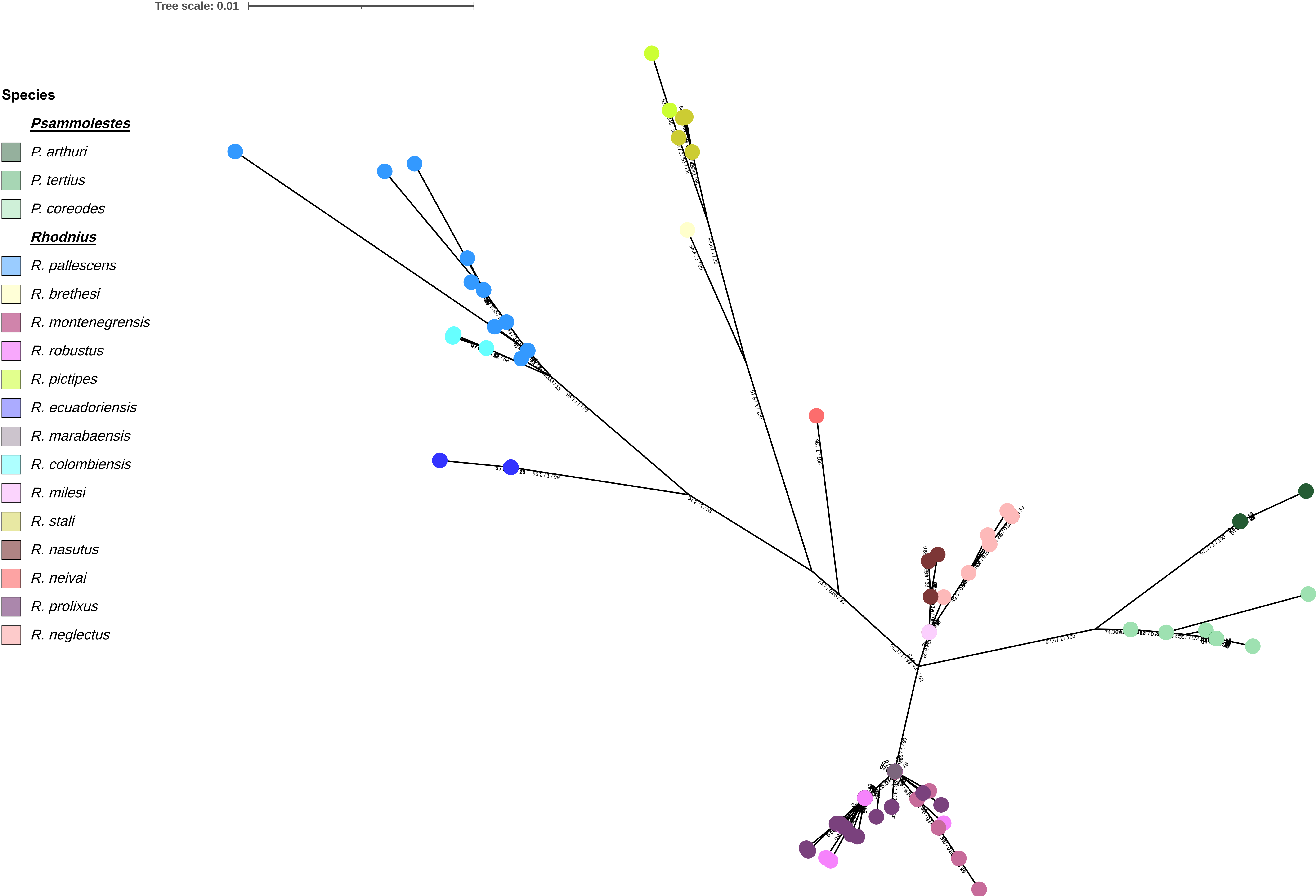

Supplementary Figure 16. CISP Phylogenetic reconstruction. Phylogenetic reconstruction with the ML algorithm based on the nuclear marker CISP. The reconstruction was performed using the Maximum Likelihood algorithm with IQTree. Bootstrap values on the internal nodes are shown in the following order: SH-aLRT/aBayes/ultrafast bootstrap support.

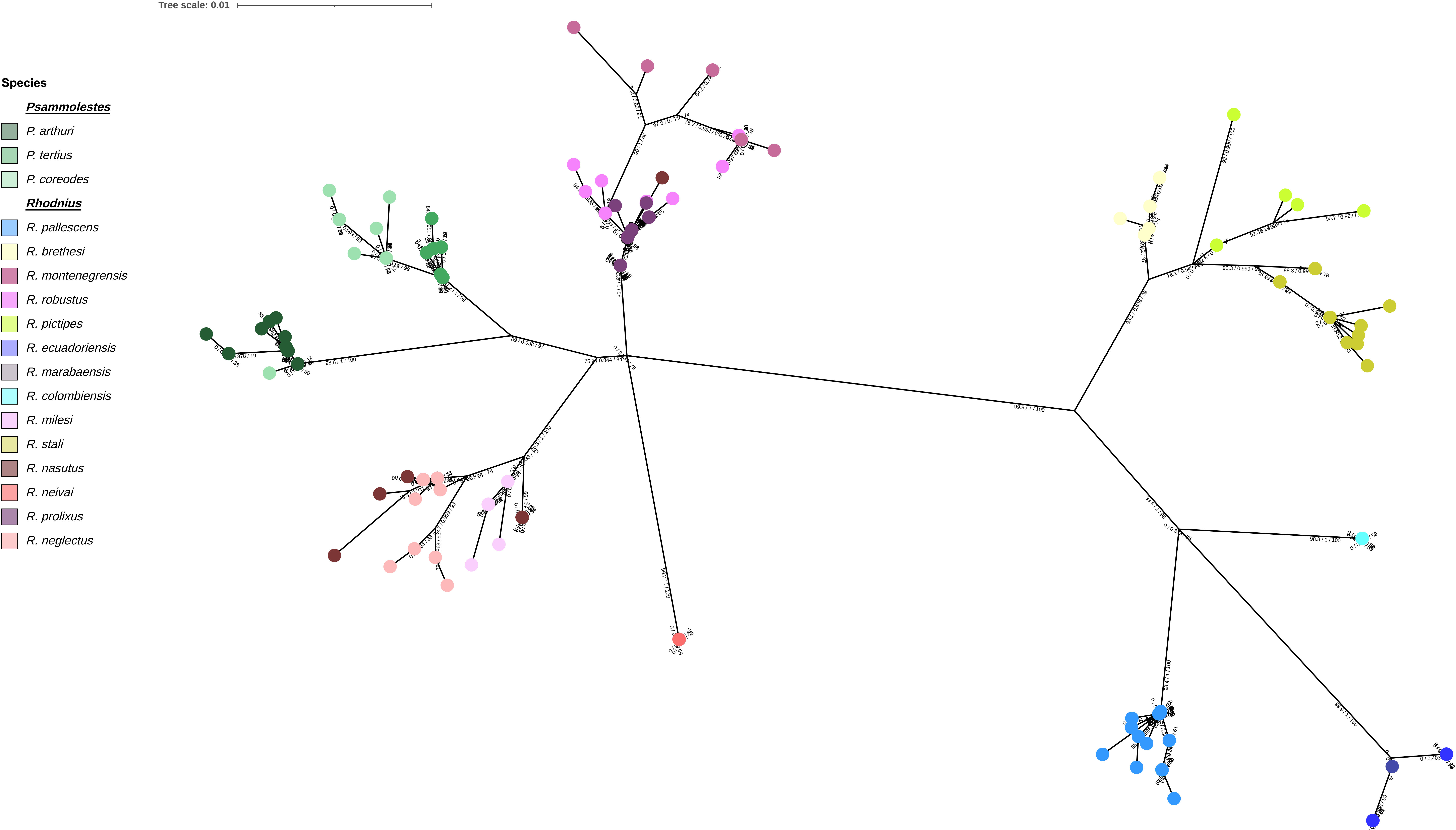

Supplementary Figure 17. PJH Phylogenetic reconstruction. Phylogenetic reconstruction with the ML algorithm based on the nuclear marker PJH. The reconstruction was performed using the Maximum Likelihood algorithm with IQTree. Bootstrap values on the internal nodes are shown in the following order: SH-aLRT/aBayes/ultrafast bootstrap support.

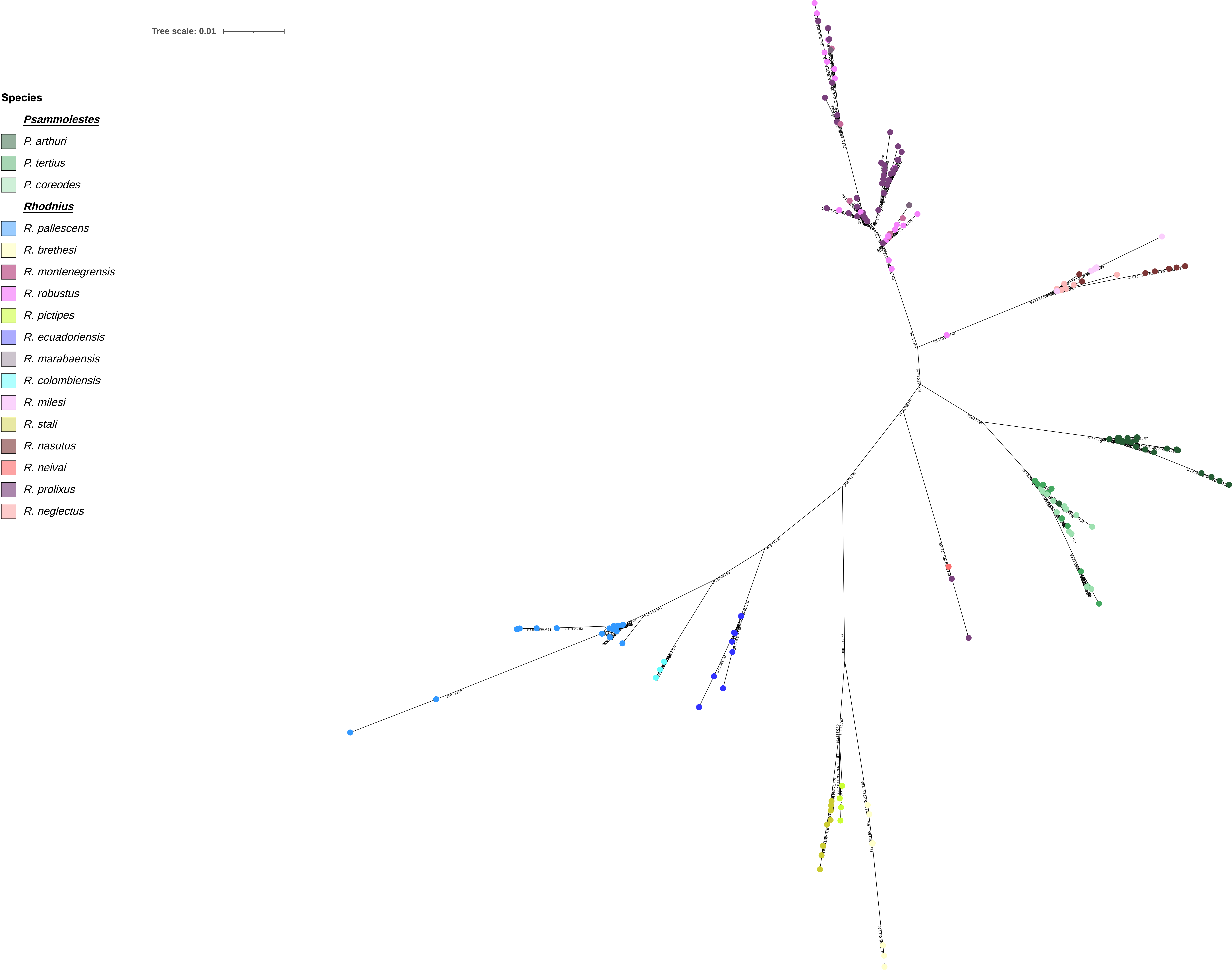

Supplementary Figure 18. TRNA Phylogenetic reconstruction. Phylogenetic reconstruction with the ML algorithm based on the nuclear marker TRNA. The reconstruction was performed using the Maximum Likelihood algorithm with IQTree. Bootstrap values on the internal nodes are shown in the following order: SH-aLRT/aBayes/ultrafast bootstrap support.

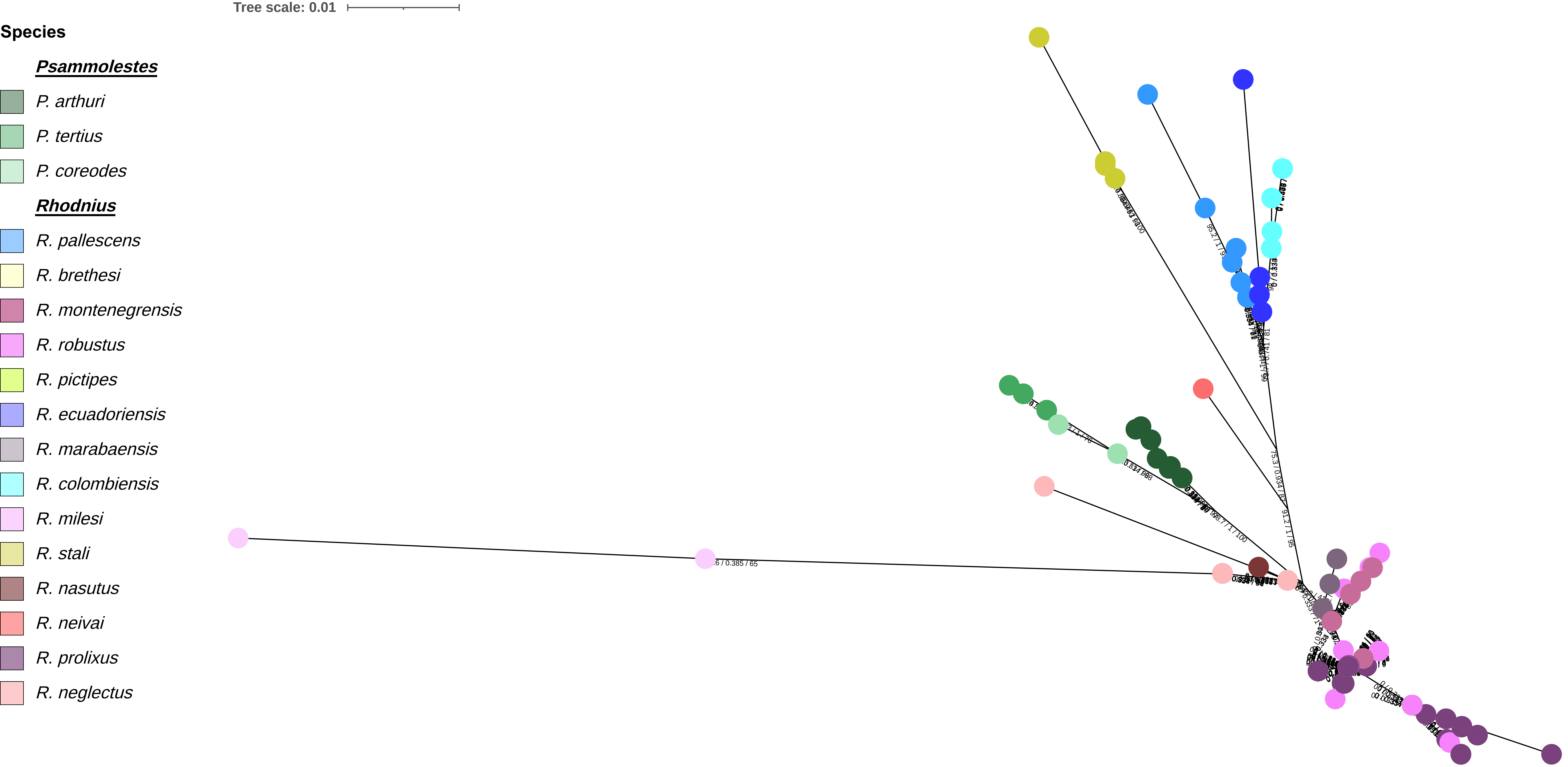

Supplementary Figure 19. UPMETAL Phylogenetic reconstruction. Phylogenetic reconstruction with the ML algorithm based on the nuclear loci UPMETAL. The reconstruction was performed using the Maximum Likelihood algorithm with IQTree. Bootstrap values on the internal nodes are shown in the following order: SH-aLRT/aBayes/ultrafast bootstrap support.

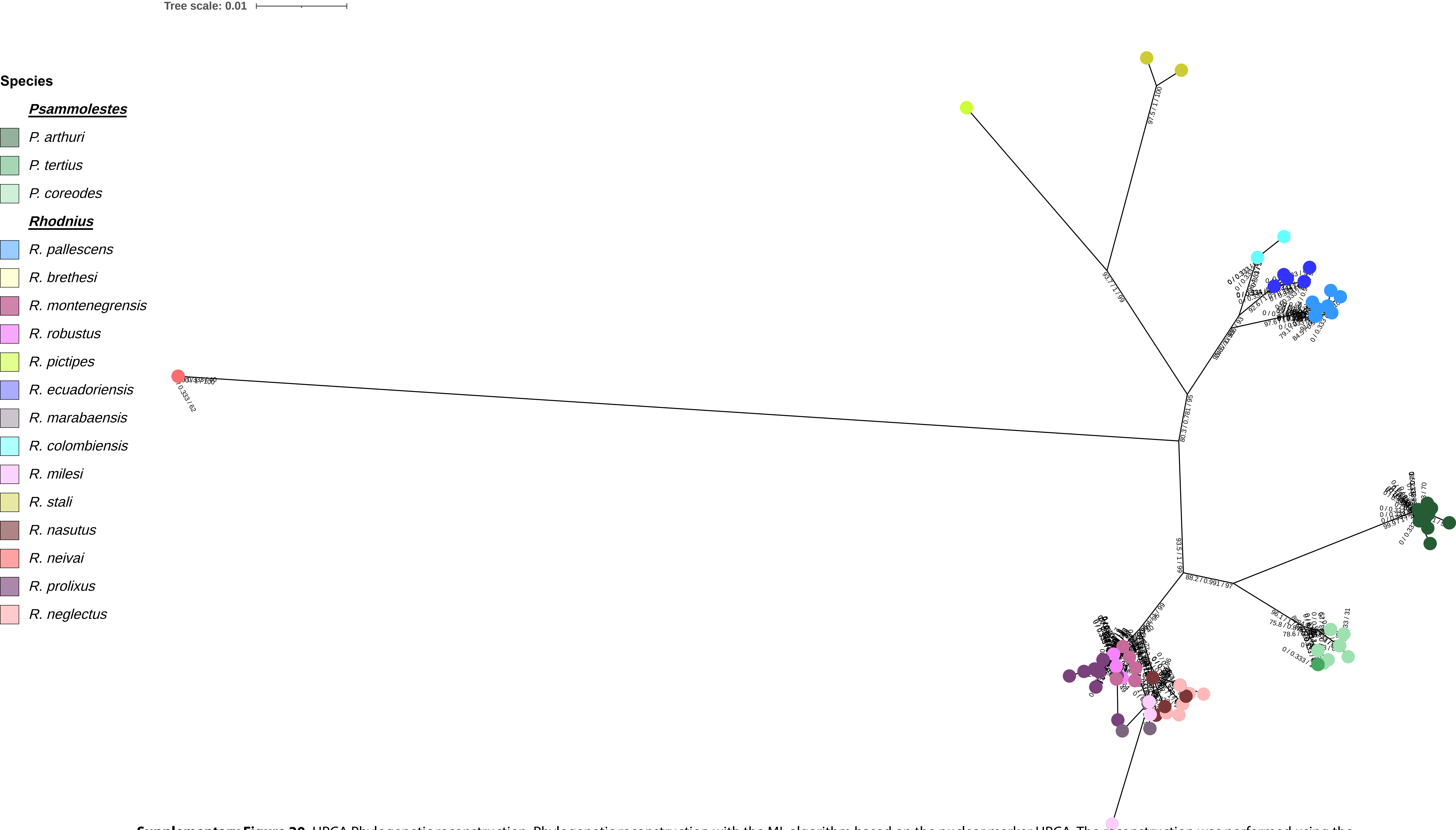

Supplementary Figure 20. UPCA Phylogenetic reconstruction. Phylogenetic reconstruction with the ML algorithm based on the nuclear marker UPCA. The reconstruction was performed using the Maximum Likelihood algorithm with IQTree. Bootstrap values on the internal nodes are shown in the following order: SH-aLRT/aBayes/ultrafast bootstrap support.

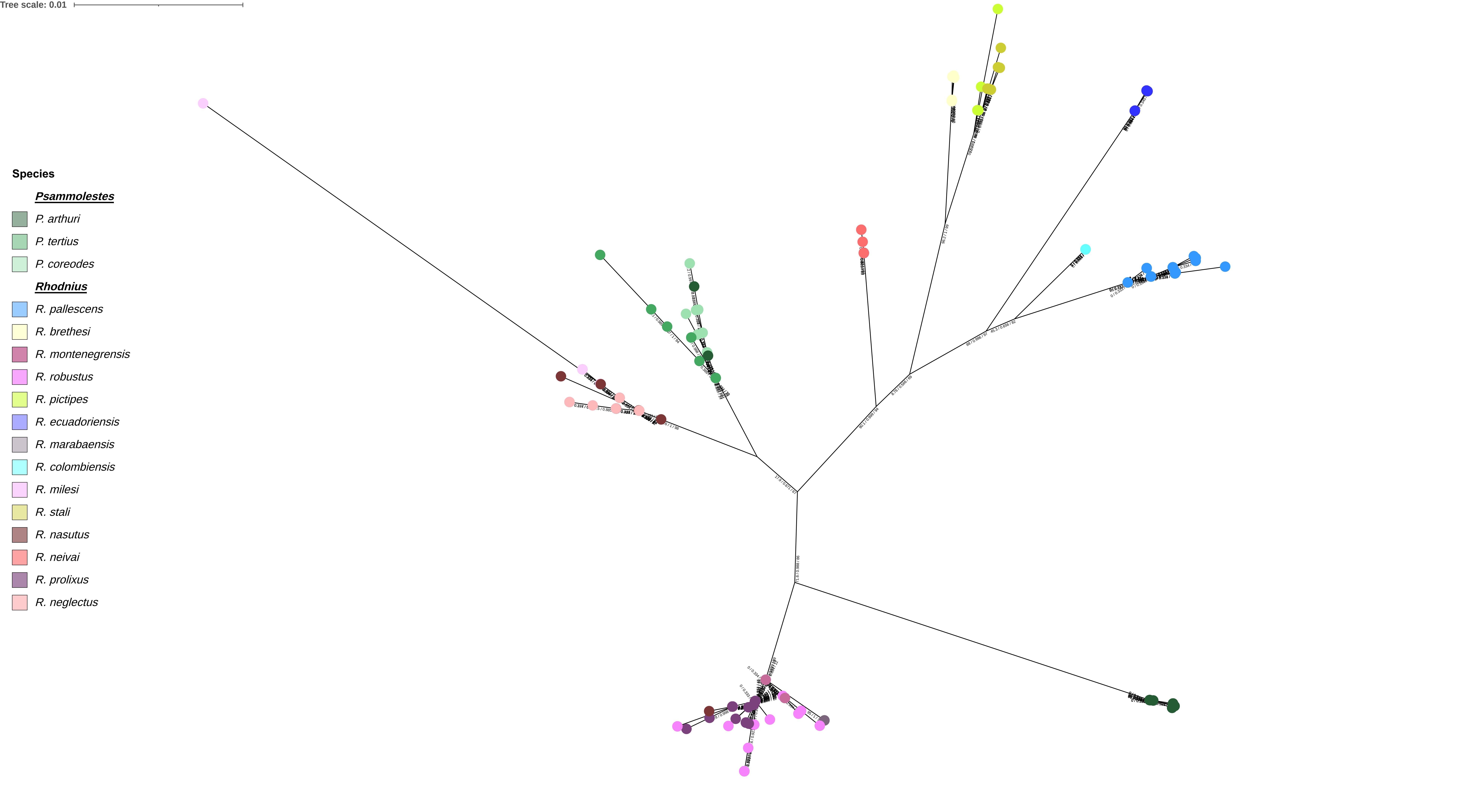

Supplementary Figure 21. LSM Phylogenetic reconstruction. Phylogenetic reconstruction with the ML algorithm based on the nuclear marker UPCA. The reconstruction was performed using the Maximum Likelihood algorithm with IQTree. Bootstrap values on the internal nodes ar shown in the following order: SH-aLRT/aBayes/ultrafast bootstrap support.

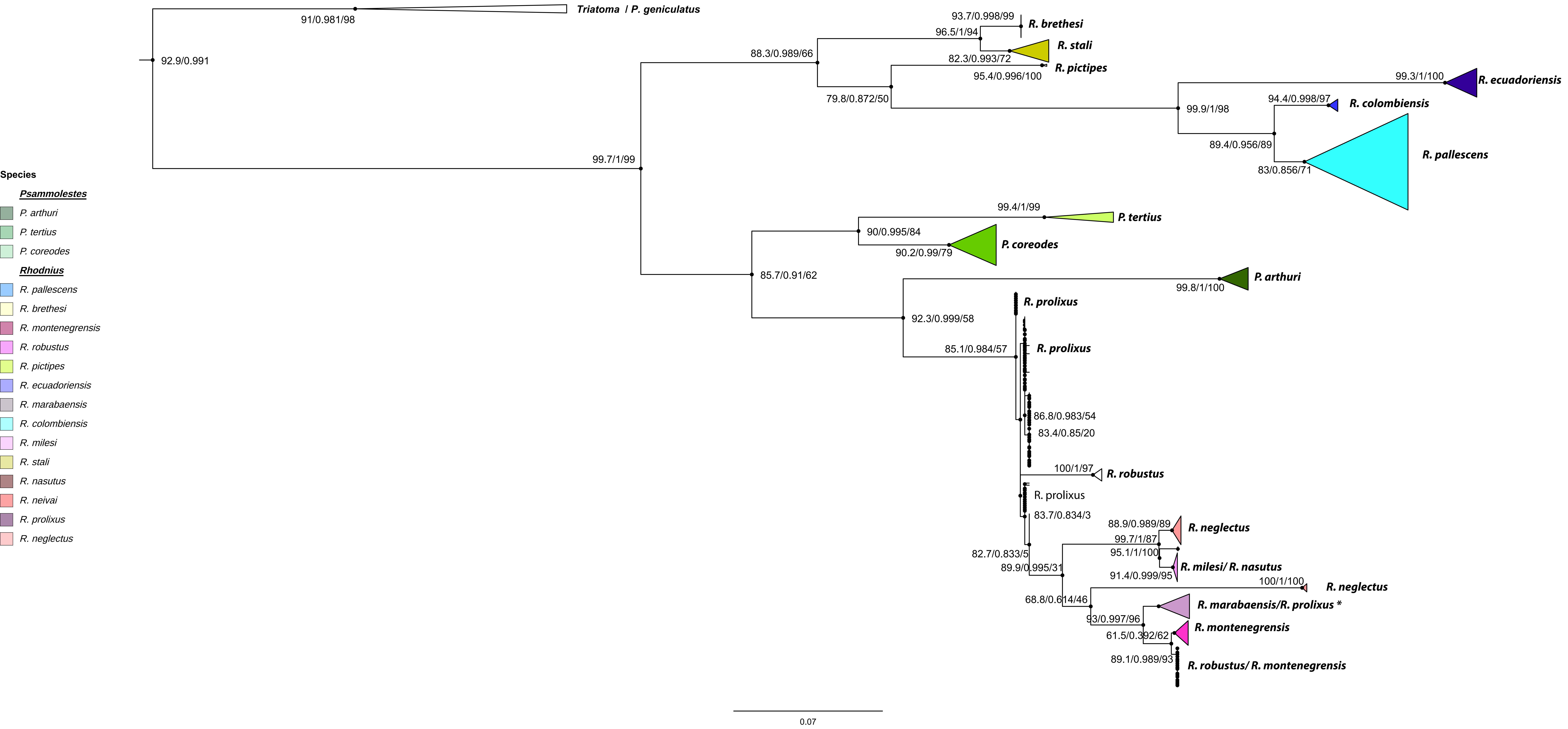

Supplementary Figure 22. CYTB Phylogenetic reconstruction. Phylogenetic reconstruction with the ML algorithm based on the mitochondrial loci CYTB. The reconstruction was performed using the Maximum Likelihood algorithm with IQTree. Bootstrap values on the internal nodes are shown in the following order: SH-aLRT/aBayes/ultrafast bootstrap support.

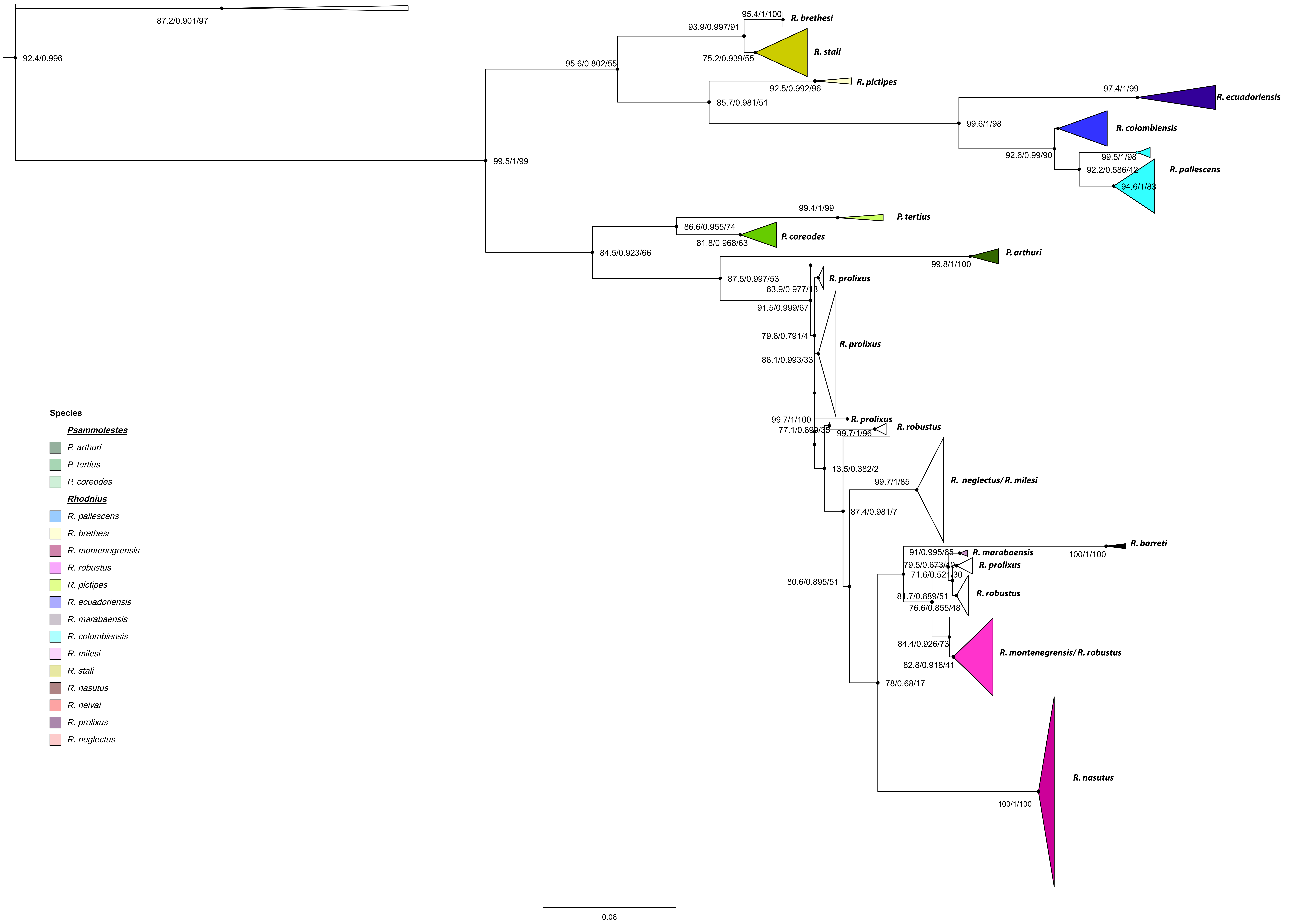

Supplementary Figure 23. CYTB Phylogenetic reconstruction of Rhodniini tribe using sequences from this study and GenBank. Phylogenetic reconstruction with the ML algorithm based on the mitochondrial loci CYTB. The reconstruction was performed using the Maximum Likelihood algorithm with IQTree. Bootstrap values on the internal nodes are shown in the following order: SH-aLRT/aBayes/ultrafast bootstrap support.

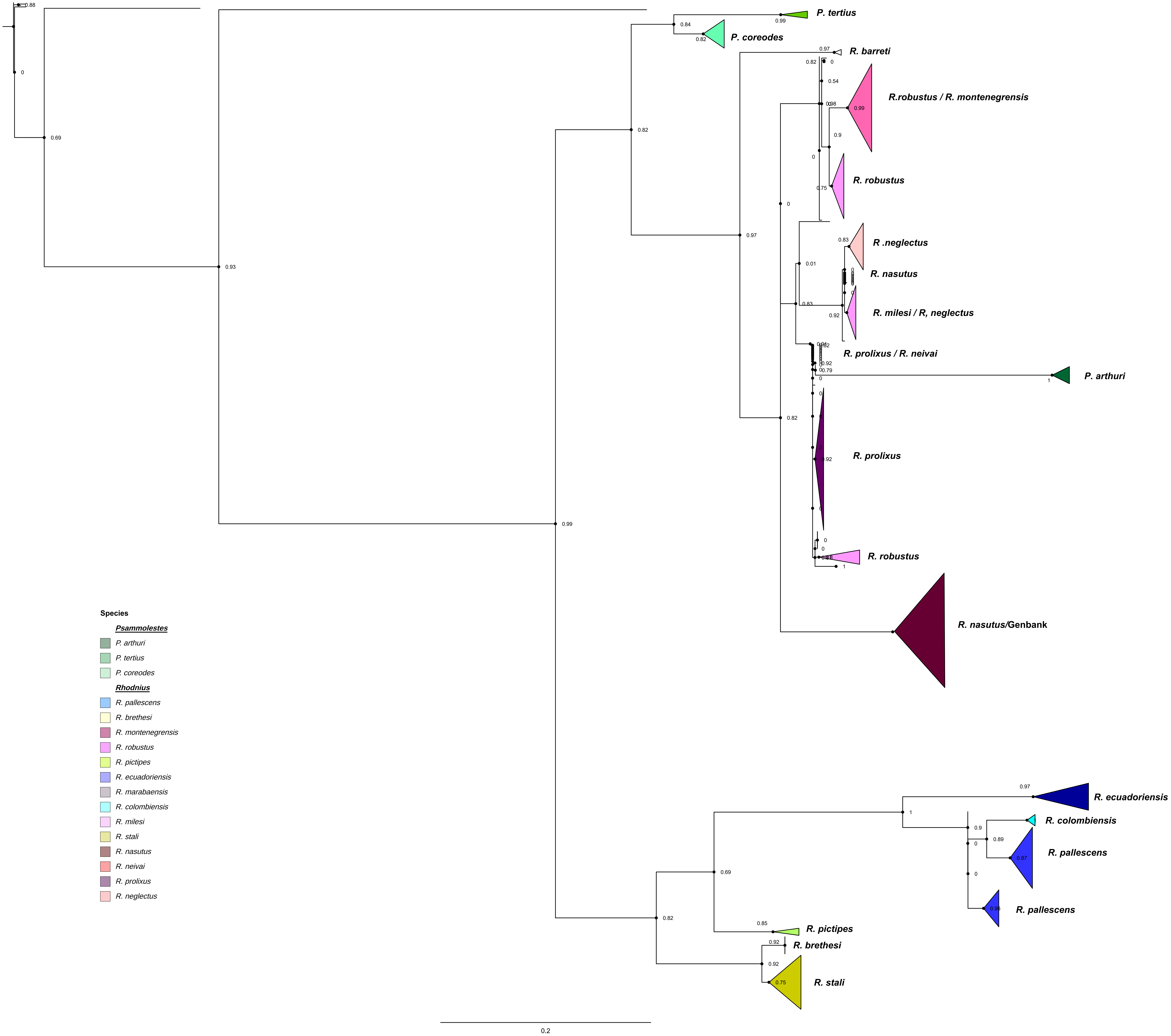

Supplementary Figure 24. CYTB Phylogenetic reconstruction. Phylogenetic reconstruction with the ML algorithm based on the mitochondrial loci CYTB. The reconstruction was performed using the Maximum Likelihood algorithm with PHYML and 1000 bootstrap repetitions. Bootstrap values are indicated in the nodes.

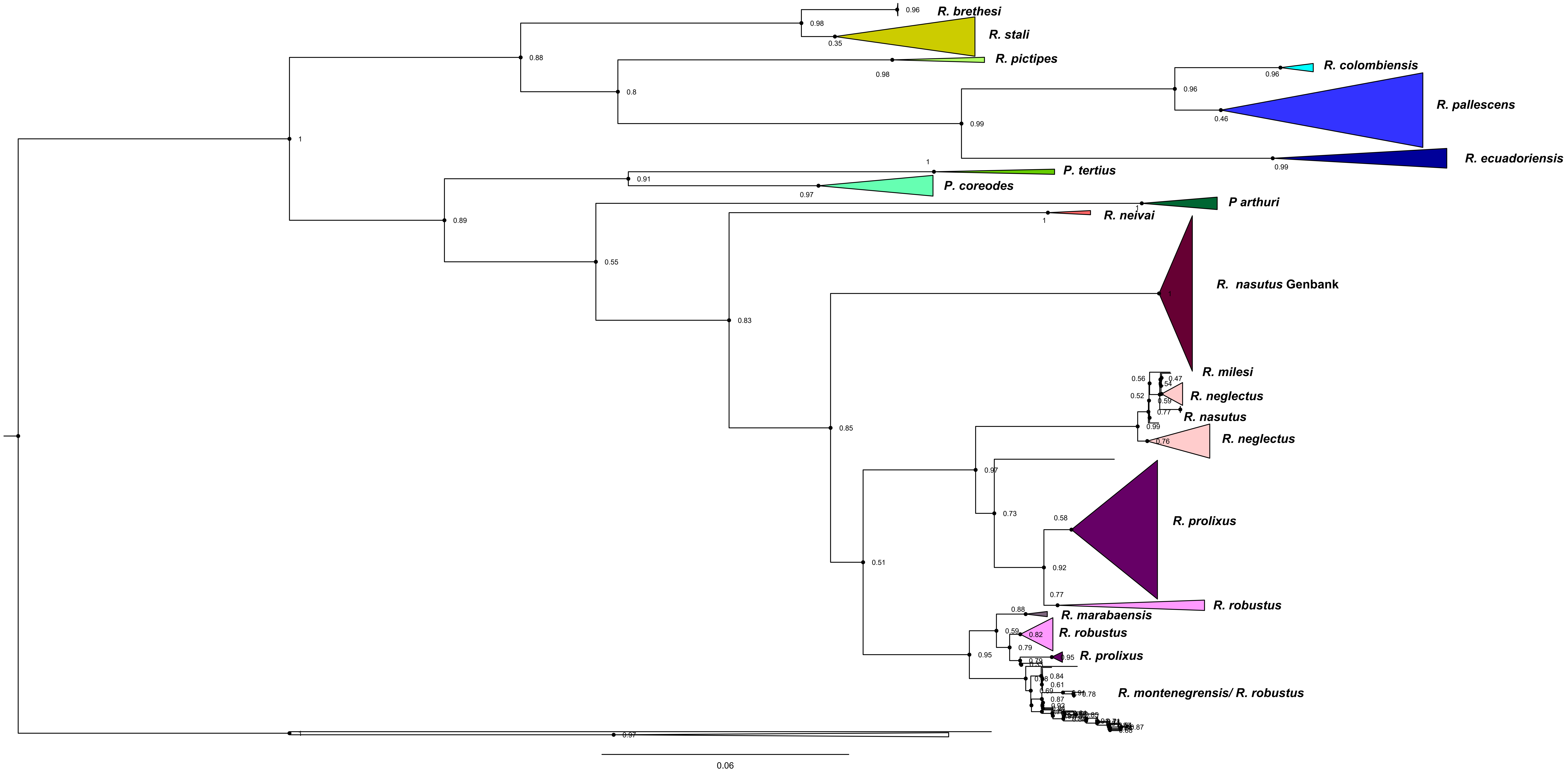
R. brethesi
R. stali
R. pictipes
R. colombiensis
R. pallescens
R. ecuadoriensis
P. tertius
P. coreodes
P arthuri
R. neivai
R. nasutus Genbank
R. milesi
R. neglectus
R. nasutus
R. neglectus
R. prolixus
R. robustus
R. marabaensis
R. robustus
R. prolixus
R. montenegrensis/ R. robustus
0.06

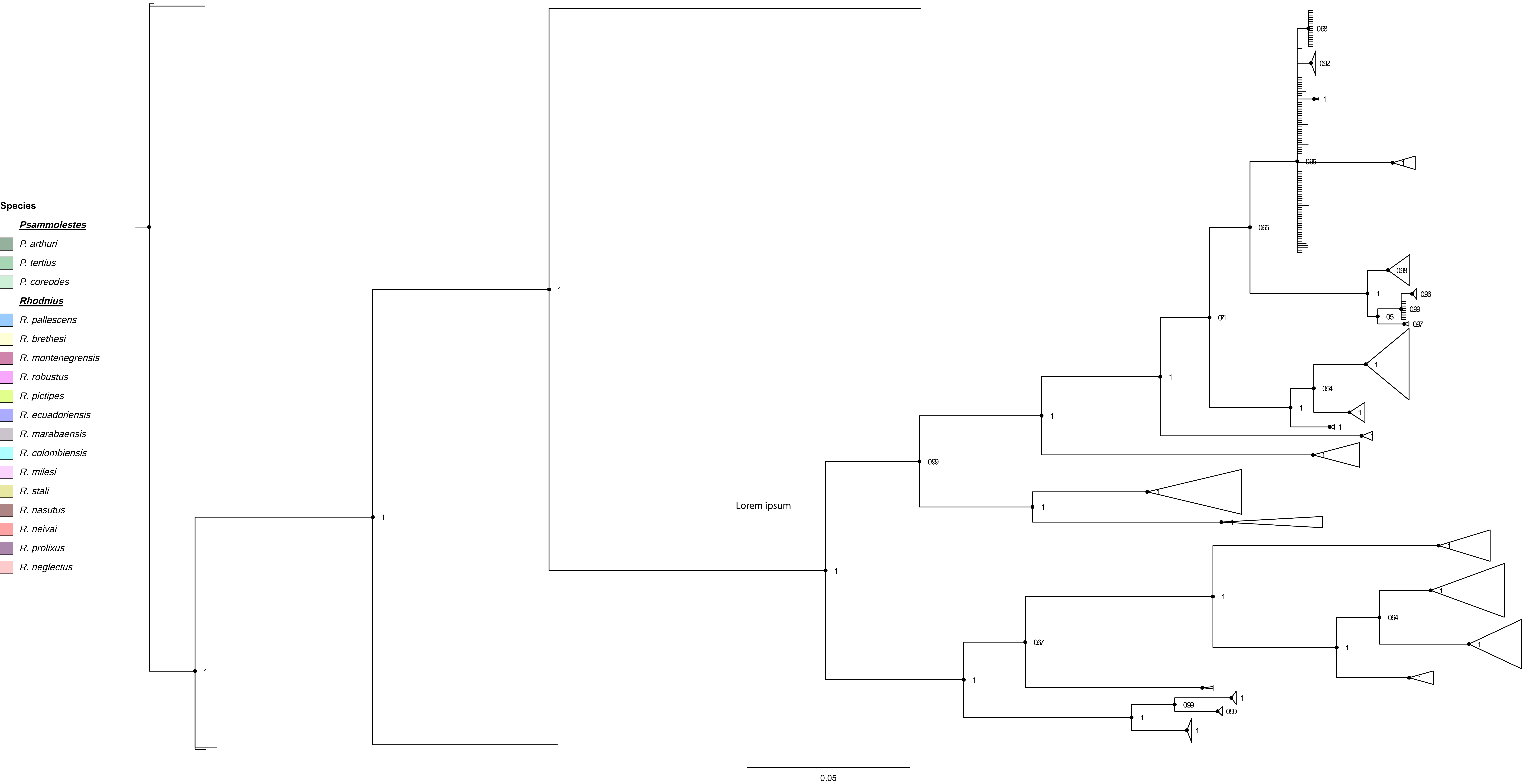

Supplementary Figure 26. CYTB phylogenetic tree using MrBayes 3.2. Phylogenetic reconstruction with the BI algorithm based on the mitochondrial loci CYTB. The reconstruction was performed using Bayesian Inference algorithm with MrBayes 3.2. Posterior probability values of internal nodes are shown.

Supplementary Figure 27. Divergence time estimation; purple bars represent the 95% highest posterior density (HPD) intervals for node divergence times. Species and genera are color coded.

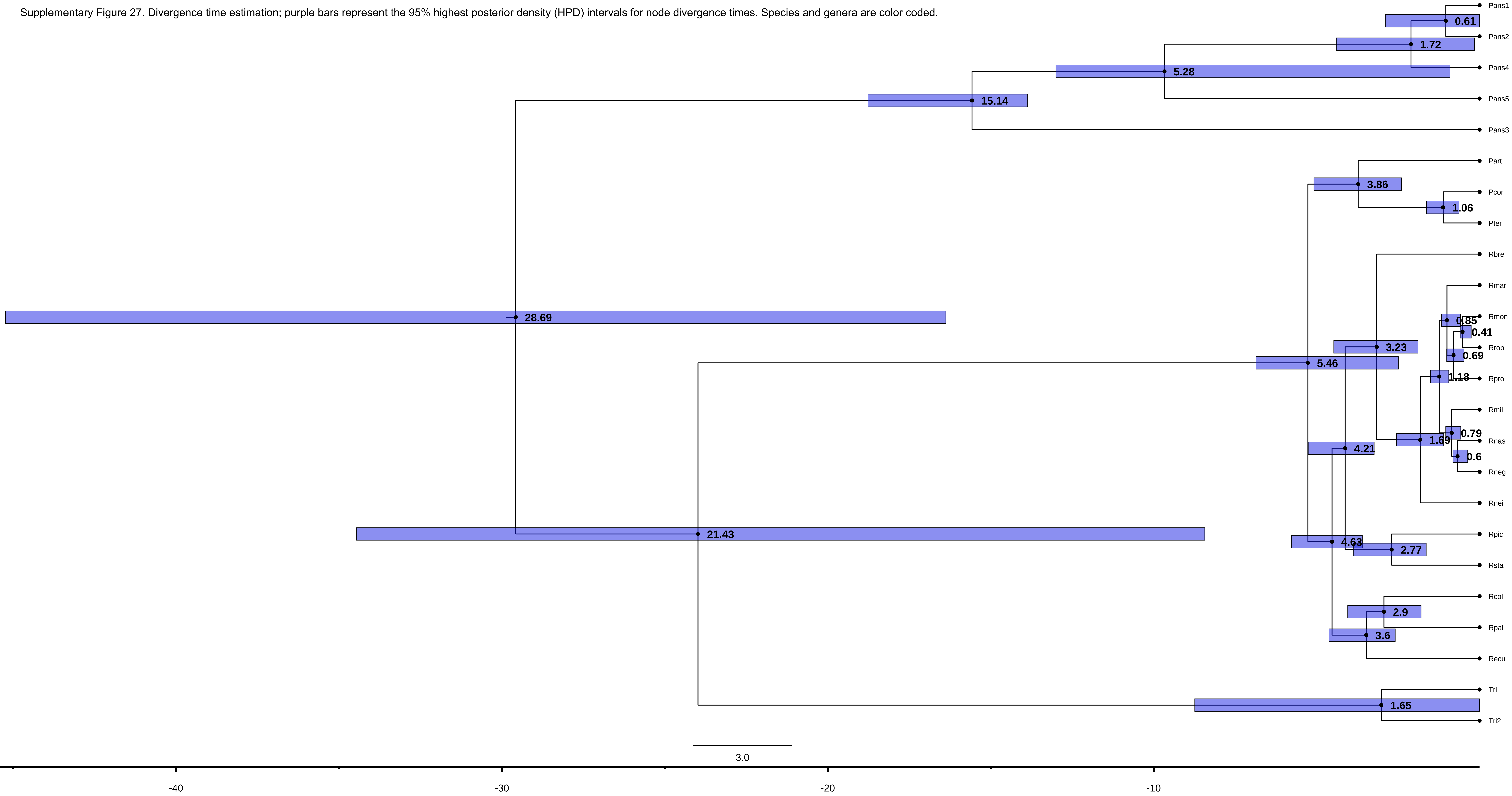

Supplementary Figure 28. Divergence time estimation; purple bars represent the 95% highest posterior density (HPD) intervals for node divergence times. Species and genera are color coded.

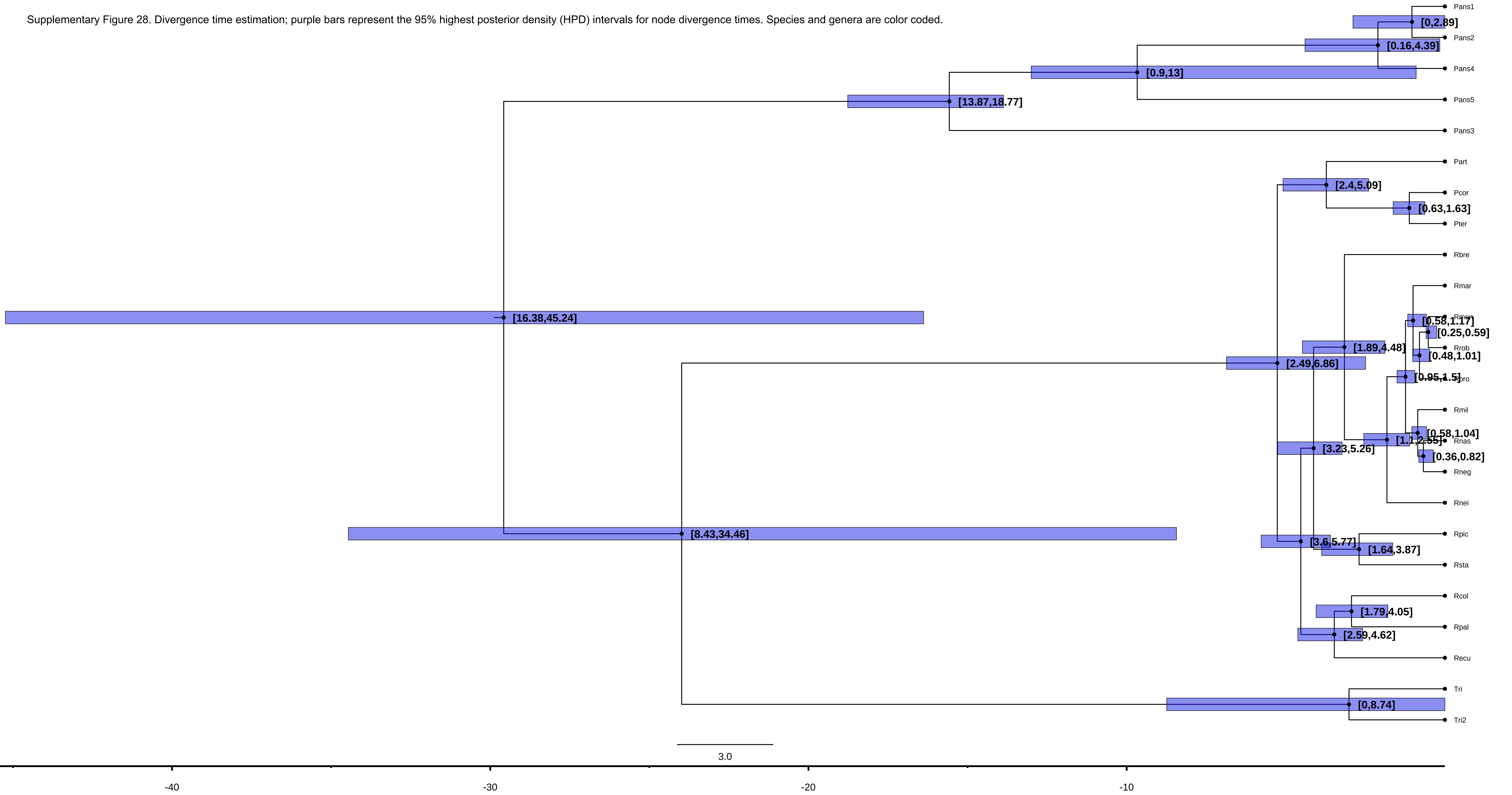

FST Species

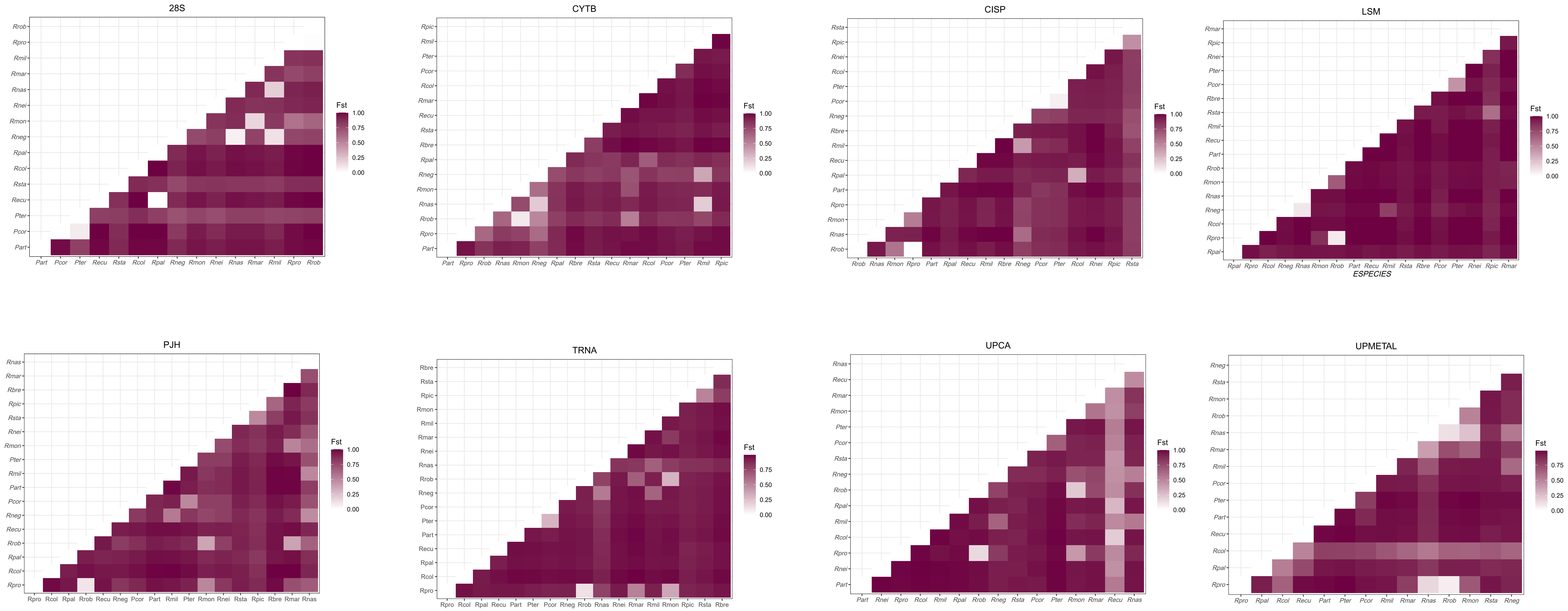

Supplementary Figure 29. Fst for all species of the Rhodniini tribe. Heatmaps calculated for Fst statistic for all species of the Rhodniini tribe based on the molecular data obtained from the eight nuclear loci analyzed in this study.

Da Species

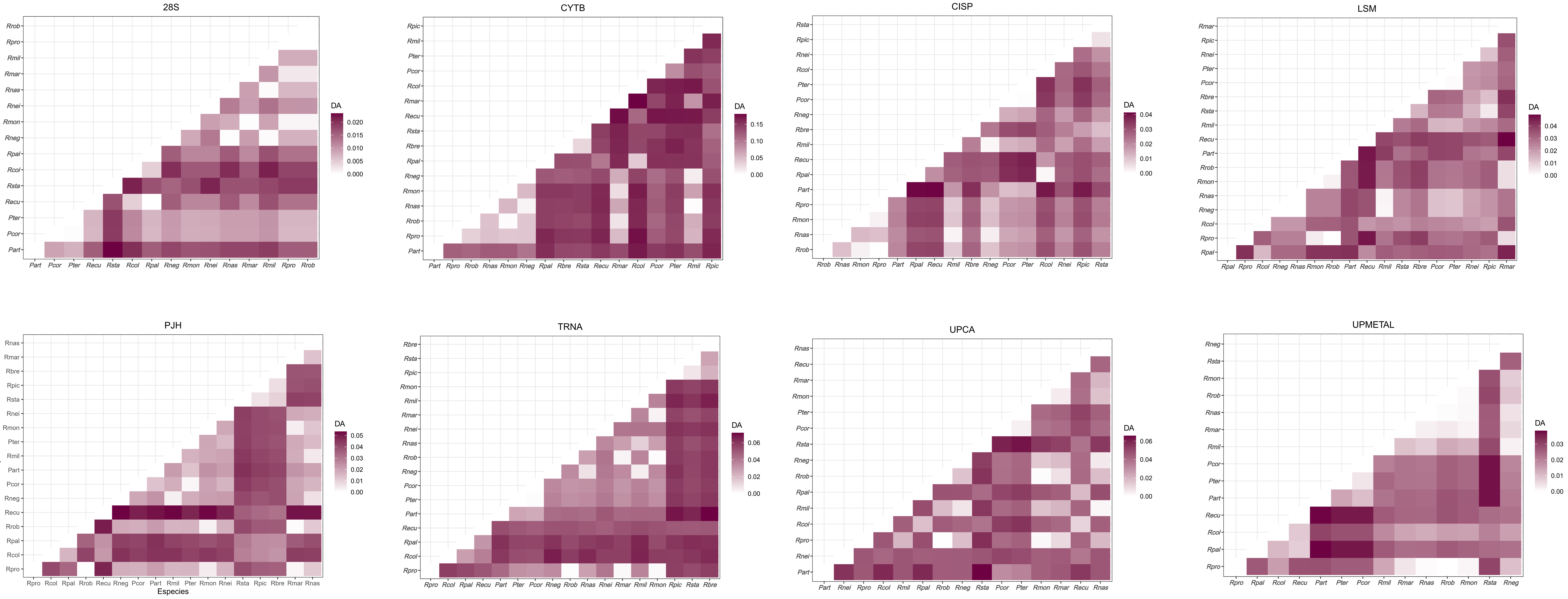

Supplementary Figure 30. Da for all species of the Rhodniini tribe. Heatmaps calculated for Da statistic for all species of the Rhodniini tribe based on the molecular data obtained from the eight nuclear loci analyzed in this study.

Dxy Species

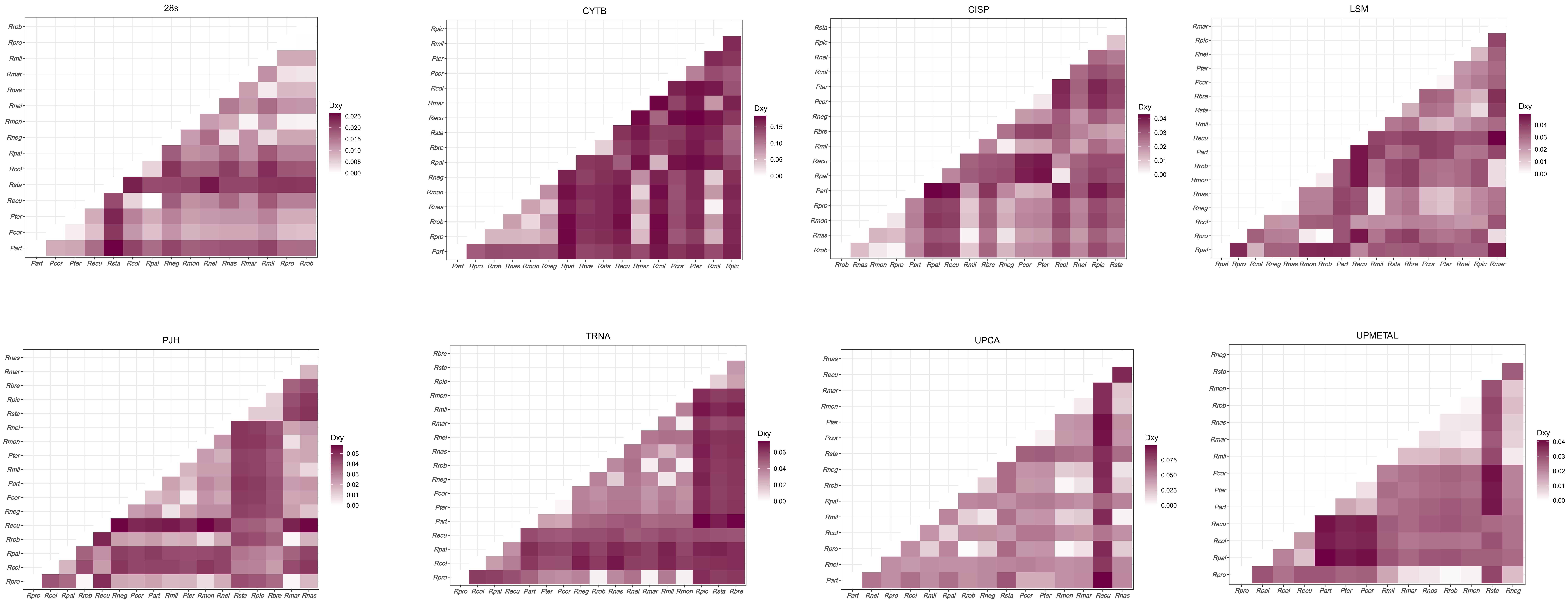

Supplementary Figure 31. Dxy for all species of the Rhodniini tribe. Heatmaps calculated for Dxy statistic for all species of the Rhodniini tribe based on the molecular data obtained from the eight nuclear loci analyzed in this study.

FST Groups

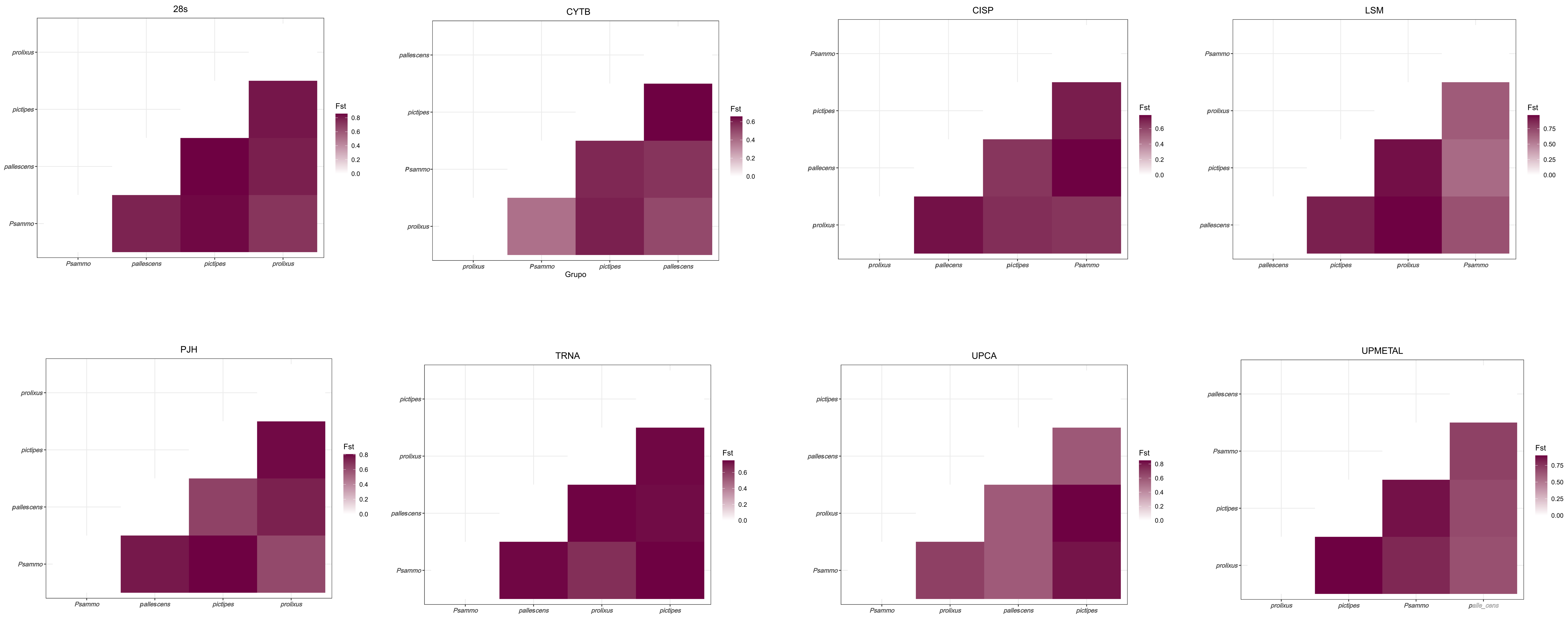

Supplementary Figure 32. Fst for groups of the Rhodniini tribe. Heatmaps calculated for Fst statistic for all species of the Rhodniini tribe based on the molecular data obtained from the eight nuclear loci analyzed in this study

Da groups

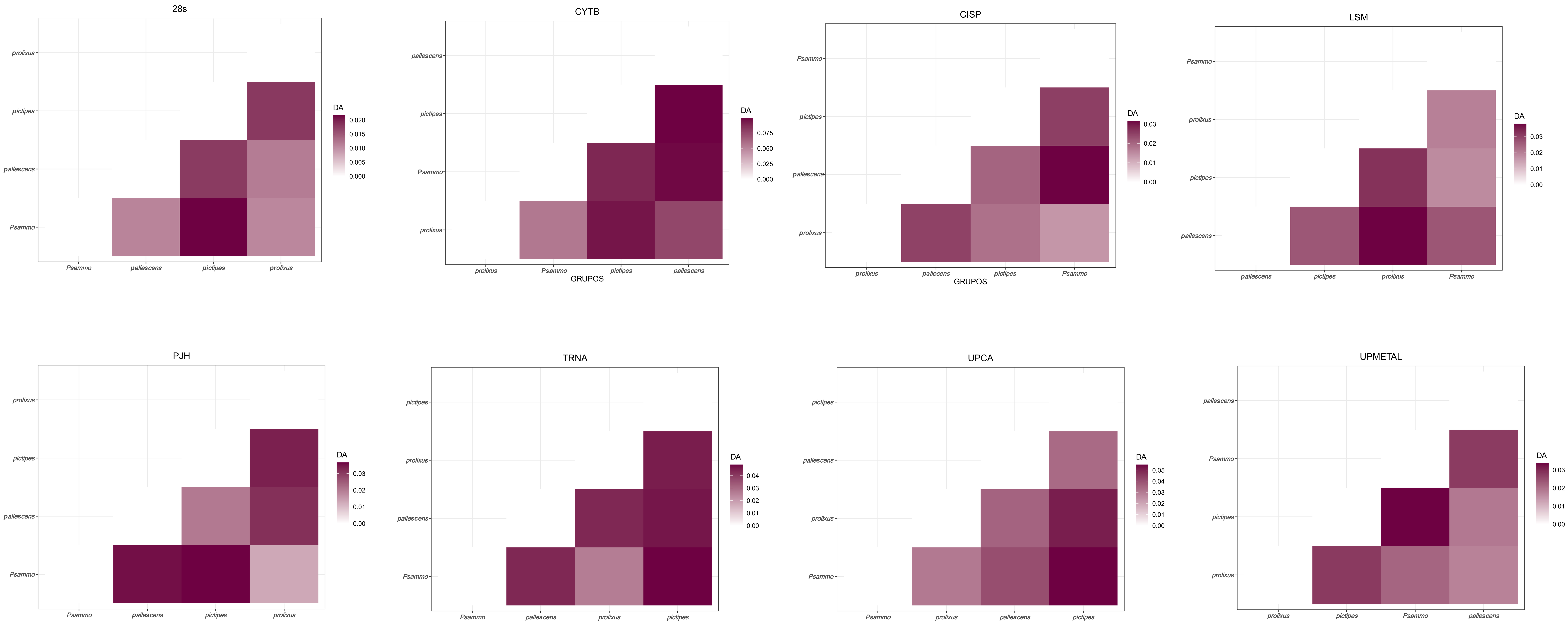

Supplementary Figure 33. Da for groups of the Rhodniini tribe. Heatmaps calculated for Da statistic for all species of the Rhodniini tribe based on the molecular data obtained from the eight nuclear loci analyzed in this study

Dxy groups

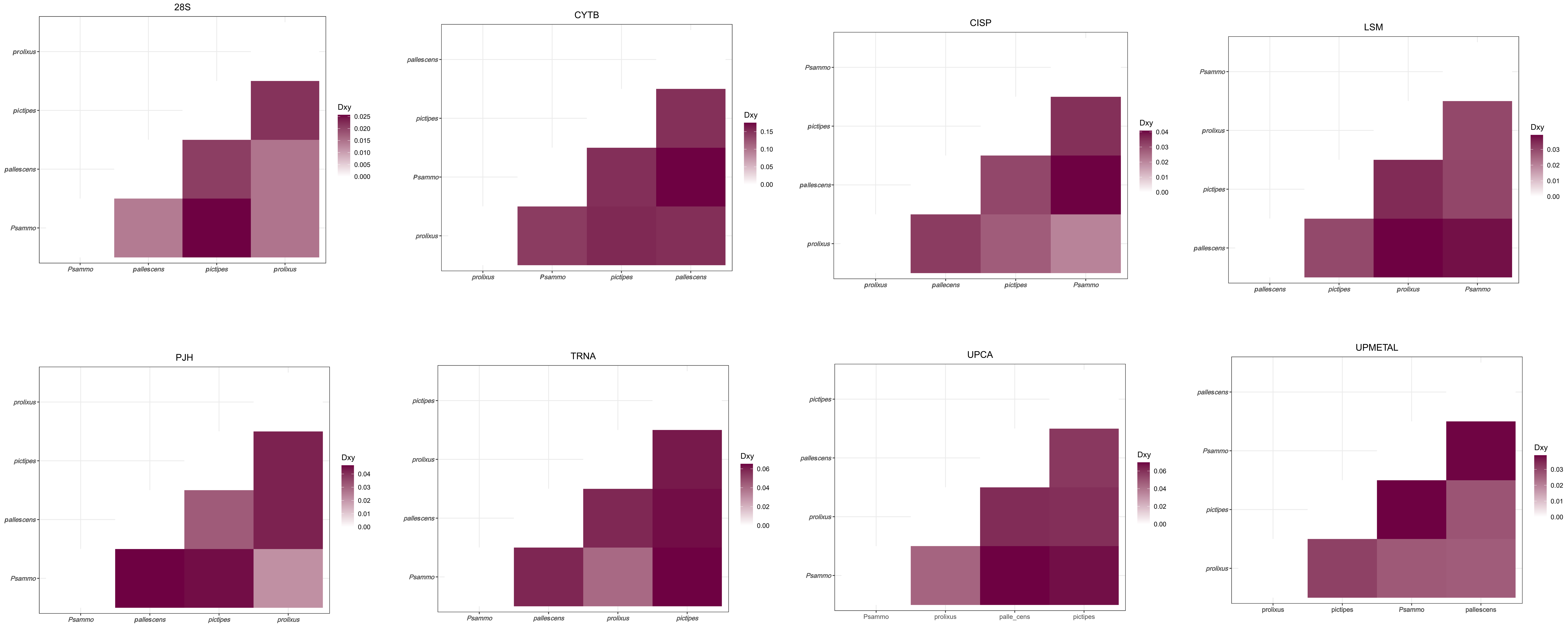

Supplementary Figure 34. Dxy for groups of the Rhodniini tribe. Heatmaps calculated for Dxy statistic for all species of the Rhodniini tribe based on the molecular data obtained from the eight nuclear loci analyzed in this study

Results from the Structure Harvester Evanno method (K=20)

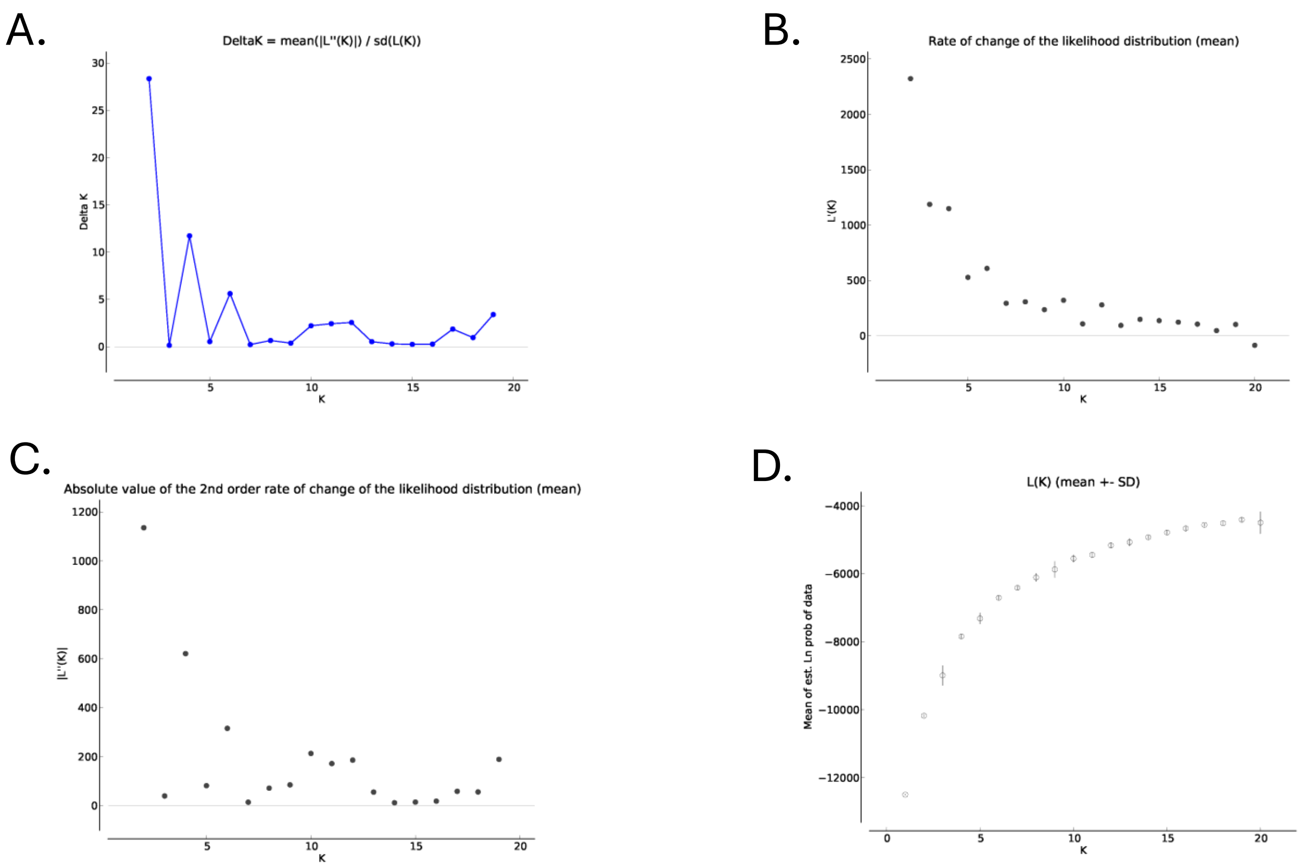

Supplementary Figure 35. Results from the Structure Harvester Evanno method (K=20). The analysis comprised 100,000 Markov Chain Monte Carlo (MCMC) generations, sampling K values from 1 to 20 with 5 iterations per K. A. Delta K B. Rate of change of the likelihood distribution. C. Absolute value of the 2nd order rate of change of the likelihood distribution D. L(K).

Supplementary Table 1. Sample information and GenBank accession numbersof individuals included in this study

ID	Number	Group	Species name	Country	State	Town	Latitude	Longitude	28s		CISP	TRNA		CYTB	UPMETAL		UPCA		PJH		LSM	
224_Part	224	*Psammolestes*	*P.arthuri*	Colombia	Casanare	Mani	4.818055556	-72.28111111	PQ585911						PV633165		PV632837		PV633522			
225_Part	225	*Psammolestes*	*P.arthuri*	Colombia	Casanare	Mani	4.818055556	-72.28111111	PQ585882			PV617795			PV633187		PV632836		PV633454		PV632174	
226_Part	226	*Psammolestes*	*P.arthuri*	Colombia	Casanare	Mani	4.818055556	-72.28111111	PQ586032			PV618067	PV618068		PV633159				PV633453		PV632175	
227_Part	227	*Psammolestes*	*P.arthuri*	Colombia	Casanare	Mani	4.818055556	-72.28111111	PQ586012		PQ605822	PV618171	PV618172				PV632838		PV633537		PV632176	
228_Part	228	*Psammolestes*	*P.arthuri*	Colombia	Casanare	Mani	4.818055556	-72.28111111	PQ585883								PV632841		PV633392			
229_Part	229	*Psammolestes*	*P.arthuri*	Colombia	Casanare	Mani	4.818055556	-72.28111111	PQ585884						PV633316				PV633529		PV632177	
230_Part	230	*Psammolestes*	*P.arthuri*	Colombia	Casanare	Mani	4.818055556	-72.28111111	PQ585885			PV618011	PV618012		PV633322		PV632849		PV633493		PV632178	
231_Part	231	*Psammolestes*	*P.arthuri*	Colombia	Casanare	Mani	4.818055556	-72.28111111	PQ585971						PV633160		PV632813		PV633564			
232_Part	232	*Psammolestes*	*P.arthuri*	Colombia	Casanare	Mani	4.818055556	-72.28111111			PQ605823	PV618189	PV618190		PV633166		PV632814		PV633565		PV632179	
233_Part	233	*Psammolestes*	*P.arthuri*	Colombia	Casanare	Mani	4.818055556	-72.28111111	PQ585846			PV618013	PV618014		PV633323		PV632842		PV633429		PV632180	
234_Part	234	*Psammolestes*	*P.arthuri*	Colombia	Casanare	Mani	4.818055556	-72.28111111	PQ585940								PV632839		PV633378		PV632181	
514_Part	514	*Psammolestes*	*P.arthuri*	Venezuela	Aragua	Maracay	10.23535	-67.59113	PQ586027			PV617880		PV632485			PV632833		PV633654		PV632302	
515_Part	515	*Psammolestes*	*P.arthuri*	Venezuela	Aragua	Maracay	10.23535	-67.59113	PQ585848		PQ605885	PV617843		PV632487			PV632834		PV633387		PV632303	
516_Part	516	*Psammolestes*	*P.arthuri*	Venezuela	Aragua	Maracay	10.23535	-67.59113				PV617844		PV632489			PV632835		PV633404		PV632304	
517_Part	517	*Psammolestes*	*P.arthuri*	Venezuela	Aragua	Maracay	10.23535	-67.59113						PV632486			PV632840				PV632305	
518_Part	518	*Psammolestes*	*P.arthuri*	Venezuela	Aragua	Maracay	10.23535	-67.59113						PV632491								
689_Part	689	*Psammolestes*	*P.arthuri*	Venezuela	Aragua	Maracay	10.23535	-67.59113	PQ586041			PV617856							PV633360		PV632360	
690_Part	690	*Psammolestes*	*P.arthuri*	Venezuela	Aragua	Maracay	10.23535	-67.59113	PQ585934			PV617857		PV632810			PV632829		PV633374		PV632361	
691_Part	691	*Psammolestes*	*P.arthuri*	Venezuela	Aragua	Maracay	10.23535	-67.59113	PQ586029			PV617858					PV632830		PV633488		PV632413	
692_Part	692	*Psammolestes*	*P.arthuri*	Venezuela	Aragua	Maracay	10.23535	-67.59113				PV617882					PV632831					
693_Part	693	*Psammolestes*	*P.arthuri*	Venezuela	Aragua	Maracay	10.23535	-67.59113	PQ585909			PV617859					PV632832		PV633701	PV633702	PV632362	
705_Part	705	*Psammolestes*	*P.arthuri*	Venezuela	Aragua	Maracay	10.23535	-67.59113	PQ586021		PQ605988	PV617860		PV632805			PV632821		PV633376			
706_Part	706	*Psammolestes*	*P.arthuri*	Venezuela	Aragua	Maracay	10.23535	-67.59113	PQ585910			PV617911									PV632365	
707_Part	707	*Psammolestes*	*P.arthuri*	Venezuela	Aragua	Maracay	10.23535	-67.59113	PQ586083	PQ586084	PQ605962	PV617865					PV632822		PV633600			
709_Part	709	*Psammolestes*	*P.arthuri*	Venezuela	Aragua	Maracay	10.23535	-67.59113			PQ605989	PV617902					PV632823		PV633375		PV632429	
711_Part	711	*Psammolestes*	*P.arthuri*	Venezuela	Aragua	Maracay	10.23535	-67.59113						PV632488								
712_Part	712	*Psammolestes*	*P.arthuri*	Venezuela	Aragua	Maracay	10.23535	-67.59113	PQ586043			PV617942		PV632490			PV632824		PV633377		PV632366	
715_Part	715	*Psammolestes*	*P.arthuri*	Venezuela	Aragua	Maracay	10.23535	-67.59113						PV632484								
727_Part	727	*Psammolestes*	*P.arthuri*	Colombia	Casanare	Paz de Ariporo	5.883	-71.883			PQ605920	PV617924							PV633577		PV632411	
728_Part	728	*Psammolestes*	*P.arthuri*	Colombia	Casanare	Paz de Ariporo	5.883	-71.883			PQ605945	PV617948							PV633596		PV632428	
729_Part	729	*Psammolestes*	*P.arthuri*	Colombia	Casanare	Paz de Ariporo	5.883	-71.883	PQ585936			PV617912							PV633400			
731_Part	731	*Psammolestes*	*P.arthuri*	Colombia	Casanare	Tamara	5.817	-72.167	PQ586079	PQ586080		PV617913			PV633146				PV633597		PV632370	
732_Part	732	*Psammolestes*	*P.arthuri*	Colombia	Casanare	Tamara	5.817	-72.167	PQ585942		PQ605946	PV617920							PV633611		PV632371	
733_Part	733	*Psammolestes*	*P.arthuri*	Colombia	Casanare	Tamara	5.817	-72.167			PQ605965	PV617904			PV633148		PV633093	PV633094	PV633667		PV632372	
734_Part	734	*Psammolestes*	*P.arthuri*	Colombia	Casanare	Tamara	5.817	-72.167	PQ585943													
737_Part	737	*Psammolestes*	*P.arthuri*	Colombia	Casanare	Pore	5.733	-71.983	PQ585933		PQ605919	PV617950					PV632825		PV633639		PV632373	
738_Part	738	*Psammolestes*	*P.arthuri*	Colombia	Casanare	Pore	5.733	-71.983	PQ585938		PQ605991	PV618117	PV618118				PV633095	PV633096	PV633498		PV632374	
742_Part	742	*Psammolestes*	*P.arthuri*	Colombia	Casanare	Pore	5.733	-71.983	PQ586015								PV632826		PV633549			
759_Part	759	*Psammolestes*	*P.arthuri*	Colombia	Casanare	Monterrey	4.87615	-72.8971	PQ586073	PQ586074	PQ605916	PV618121	PV618122				PV633097	PV633098	PV633499		PV632375	
760_Part	760	*Psammolestes*	*P.arthuri*	Colombia	Casanare	Monterrey	4.87615	-72.8971	PQ585939			PV617921					PV632827		PV633612		PV632388	
761_Part	761	*Psammolestes*	*P.arthuri*	Colombia	Casanare	Monterrey	4.87615	-72.8971			PQ605921	PV617864					PV632843		PV633598		PV632376	
765_Part	765	*Psammolestes*	*P.arthuri*	Colombia	Arauca	Arauca	7,08471	-70.75	PQ585945		PQ605947	PV617905					PV632828		PV633550		PV632377	
510_Pcor	510	*Psammolestes*	*P.coreodes*	Brasil	Mato Grosso do Sul	Corumba	-19.0098	-57.6547	PQ586009			PV617967		PV632672			PV632951		PV633450		PV632299	
511_Pcor	511	*Psammolestes*	*P.coreodes*	Brasil	Mato Grosso do Sul	Corumba	-19.0098	-57.6547				PV617875		PV632674	PV633179		PV632920		PV633543		PV632300	
512_Pcor	512	*Psammolestes*	*P.coreodes*	Brasil	Mato Grosso do Sul	Corumba	-19.0098	-57.6547	PQ586010		PQ605884	PV617901		PV632675	PV633151		PV633109	PV633110	PV633503		PV632301	
647_Pcor	647	*Psammolestes*	*P.coreodes*	Brasil	Mato Grosso do Sul	Corumba	-19.0098	-57.6547				PV617855					PV632929		PV633542		PV632422	
648_Pcor	648	*Psammolestes*	*P.coreodes*	Brasil	Mato Grosso do Sul	Corumba	-19.0098	-57.6547	PQ586053	PQ586054	PQ605915	PV618097	PV618098	PV632700			PV632934		PV633662		PV632469	PV632470
649_Pcor	649	*Psammolestes*	*P.coreodes*	Brasil	Mato Grosso do Sul	Corumba	-19.0098	-57.6547				PV618063	PV618064	PV632738			PV633103	PV633104	PV633495		PV632347	
650_Pcor	650	*Psammolestes*	*P.coreodes*	Brasil	Mato Grosso do Sul	Corumba	-19.0098	-57.6547				PV617907		PV632767			PV632941		PV633399			
652_Pcor	652	*Psammolestes*	*P.coreodes*	Brasil	Mato Grosso do Sul	Corumba	-19.0098	-57.6547				PV618099	PV618100	PV632766			PV633056		PV633637		PV632348	
654_Pcor	654	*Psammolestes*	*P.coreodes*	Brasil	Mato Grosso do Sul	Corumba	-19.0098	-57.6547			PQ605913	PV617935		PV632691			PV632930		PV633396			
655_Pcor	655	*Psammolestes*	*P.coreodes*	Brasil	Mato Grosso do Sul	Corumba	-19.0098	-57.6547			PQ605959	PV617936		PV632692					PV633497		PV632349	
658_Pcor	658	*Psammolestes*	*P.coreodes*	Brasil	Mato Grosso do Sul	Corumba	-19.0098	-57.6547	PQ586026			PV617863					PV632931		PV633397		PV632350	
660_Pcor	660	*Psammolestes*	*P.coreodes*	Brasil	Mato Grosso do Sul	Corumba	-19.0098	-57.6547			PQ605960	PV617937		PV632693			PV632932		PV633398		PV632471	PV632472
662_Pcor	662	*Psammolestes*	*P.coreodes*	Brasil	Mato Grosso do Sul	Corumba	-19.0098	-57.6547				PV617938		PV632694			PV632933		PV633395		PV632351	
663_Pcor	663	*Psammolestes*	*P.coreodes*	Brasil	Mato Grosso do Sul	Corumba	-19.0098	-57.6547			PQ605922			PV632695			PV632991				PV632352	PV632353
664_Pcor	664	*Psammolestes*	*P.coreodes*	Brasil	Mato Grosso do Sul	Corumba	-19.0098	-57.6547			PQ606000	PV617881					PV632935				PV632354	
665_Pcor	665	*Psammolestes*	*P.coreodes*	Brasil	Mato Grosso do Sul	Corumba	-19.0098	-57.6547	PQ586038								PV632936	PV632937				
666_Pcor	666	*Psammolestes*	*P.coreodes*	Brasil	Mato Grosso do Sul	Corumba	-19.0098	-57.6547	PQ585851			PV617908		PV632779			PV632938				PV632355	PV632356
667_Pcor	667	*Psammolestes*	*P.coreodes*	Brasil	Mato Grosso do Sul	Corumba	-19.0098	-57.6547	PQ585946		PQ605996	PV617918		PV632686					PV633485		PV632357	
669_Pcor	669	*Psammolestes*	*P.coreodes*	Brasil	Mato Grosso do Sul	Corumba	-19.0098	-57.6547	PQ586055	PQ586056	PQ605984	PV617939		PV632687			PV632939		PV633357		PV632358	
670_Pcor	670	*Psammolestes*	*P.coreodes*	Brasil	Mato Grosso do Sul	Corumba	-19.0098	-57.6547	PQ585932			PV617928		PV632688			PV632942		PV633355		PV632359	
671_Pcor	671	*Psammolestes*	*P.coreodes*	Brasil	Mato Grosso do Sul	Corumba	-19.0098	-57.6547	PQ585944		PQ605985	PV617919		PV632689								
672_Pcor	672	*Psammolestes*	*P.coreodes*	Brasil	Mato Grosso do Sul	Corumba	-19.0098	-57.6547			PQ605951	PV618105	PV618106	PV632690			PV633058		PV633487		PV632473	PV632474
679_Pcor	679	*Psammolestes*	*P.coreodes*	Brasil	Mato Grosso do Sul	Corumba	-19.0098	-57.6547			PQ605961	PV617899		PV632699			PV632943				PV632446	
680_Pcor	680	*Psammolestes*	*P.coreodes*	Brasil	Mato Grosso do Sul	Corumba	-19.0098	-57.6547	PQ586019		PQ605914	PV617940		PV632804			PV632944					
681_Pcor	681	*Psammolestes*	*P.coreodes*	Brasil	Mato Grosso do Sul	Corumba	-19.0098	-57.6547				PV617909										
682_Pcor	682	*Psammolestes*	*P.coreodes*	Brasil	Mato Grosso do Sul	Corumba	-19.0098	-57.6547	PQ586061	PQ586062	PQ605986	PV617885					PV632948				PV632475	PV632476
683_Pcor	683	*Psammolestes*	*P.coreodes*	Brasil	Mato Grosso do Sul	Corumba	-19.0098	-57.6547	PQ586046			PV618107	PV618108				PV632945					
688_Pcor	688	*Psammolestes*	*P.coreodes*	Brasil	Mato Grosso do Sul	Corumba	-19.0098	-57.6547	PQ585999			PV617910					PV632946		PV633496		PV632396	
694_Pcor	694	*Psammolestes*	*P.coreodes*	Brasil	Mato Grosso do Sul	Corumba	-19.0098	-57.6547	PQ585998		PQ605987			PV632807			PV632947		PV633609		PV632364	
499_Pter	499	*Psammolestes*	*P.tertius*	Brasil	Bahia	Castro Alves	-12.5167	-39.2				PV618087	PV618088				PV632954		PV633679	PV633680	PV632296	
500_Pter	500	*Psammolestes*	*P.tertius*	Brasil	Bahia	Castro Alves	-12.5167	-39.2	PQ586081	PQ586082	PQ605881				PV633156							
502_Pter	502	*Psammolestes*	*P.tertius*	Brasil	Bahia	Castro Alves	-12.5167	-39.2			PQ605882	PV618053	PV618054		PV633161		PV633055				PV632399	
504_Pter	504	*Psammolestes*	*P.tertius*	Brasil	Bahia	Castro Alves	-12.5167	-39.2			PQ605883				PV633157		PV632918		PV633449		PV632297	
505_Pter	505	*Psammolestes*	*P.tertius*	Brasil	Bahia	Castro Alves	-12.5167	-39.2									PV632919					
506_Pter	506	*Psammolestes*	*P.tertius*	Brasil	Bahia	Castro Alves	-12.5167	-39.2											PV633687	PV633688	PV632408	
507_Pter	507	*Psammolestes*	*P.tertius*	Brasil	Bahia	Castro Alves	-12.5167	-39.2				PV617842					PV632962		PV633383			
508_Pter	508	*Psammolestes*	*P.tertius*	Brasil	Bahia	Castro Alves	-12.5167	-39.2							PV633196						PV632298	
613_Pter	613	*Psammolestes*	*P.tertius*	Brasil	Bahia	Castro Alves	-12.5167	-39.2			PQ605909	PV617883							PV633717	PV633718	PV632337	
614_Pter	614	*Psammolestes*	*P.tertius*	Brasil	Bahia	Castro Alves	-12.5167	-39.2			PQ605910	PV617900		PV632761			PV632927		PV633569		PV632338	
615_Pter	615	*Psammolestes*	*P.tertius*	Brasil	Bahia	Castro Alves	-12.5167	-39.2			PQ605950	PV617930							PV633570		PV632339	
616_Pter	616	*Psammolestes*	*P.tertius*	Brasil	Bahia	Castro Alves	-12.5167	-39.2			PQ605956	PV617852					PV632953		PV633539		PV632340	

617_Pter	617	*Psammolestes*	*P.tertius*	Brasil	Bahia	Castro Alves	-12.5167	-39.2				PV617906					PV632988		PV633705	PV633706		
623_Pter	623	*Psammolestes*	*P.tertius*	Brasil	Bahia	Santa Teresinha	-14.4324	-49.7146			PQ605911	PV617915		PV632714			PV632940		PV633435		PV632341	
625_Pter	625	*Psammolestes*	*P.tertius*	Brasil	Bahia	Santa Teresinha	-14.4324	-49.7146				PV617929					PV632989		PV633638			
630_Pter	630	*Psammolestes*	*P.tertius*	Brasil	Bahia	Santa Teresinha	-14.4324	-49.7146				PV617884							PV633636		PV632342	
631_Pter	631	*Psammolestes*	*P.tertius*	Brasil	Bahia	Santa Teresinha	-14.4324	-49.7146	PQ585987			PV617949							PV633571		PV632343	
632_Pter	632	*Psammolestes*	*P.tertius*	Brasil	Bahia	Santa Teresinha	-14.4324	-49.7146			PQ605957	PV617931							PV633723	PV633724	PV632386	
633_Pter	633	*Psammolestes*	*P.tertius*	Brasil	Bahia	Seabra	-12.4214	-41.7668	PQ585912		PQ605958	PV617922		PV632735					PV633540		PV632344	
634_Pter	634	*Psammolestes*	*P.tertius*	Brasil	Bahia	Seabra	-12.4214	-41.7668	PQ586063	PQ586064		PV617932		PV632763					PV633480		PV632385	
635_Pter	635	*Psammolestes*	*P.tertius*	Brasil	Bahia	Seabra	-12.4214	-41.7668			PQ605982	PV617879					PV633057		PV633367		PV632345	
636_Pter	636	*Psammolestes*	*P.tertius*	Brasil	Bahia	Seabra	-12.4214	-41.7668			PQ605912	PV617853		PV632806			PV632928		PV633541		PV632346	
637_Pter	637	*Psammolestes*	*P.tertius*	Brasil	Bahia	Seabra	-12.4214	-41.7668				PV617854					PV632990		PV633481			
703_Pter	703	*Psammolestes*	*P.tertius*	Brasil	Minas Gerais	Itanhandu	-22.2978	-44.9267			PQ605923	PV617941		PV632809			PV633059		PV633500		PV632363	
321_Rbre	321	pictipes	*R.brethesi*	Brasil	Amazonas	Barcelos	-0.97166278	-62.884				PV618113	PV618114						PV633548		PV632215	
323_Rbre	323	pictipes	*R.brethesi*	Brasil	Amazonas	Barcelos	-0.97166278	-62.884				PV617888		PV632778					PV633515			
325_Rbre	325	pictipes	*R.brethesi*	Brasil	Amazonas	Barcelos	-0.97166278	-62.884				PV617890		PV632739					PV633505		PV632216	
327_Rbre	327	pictipes	*R.brethesi*	Brasil	Amazonas	Barcelos	-0.97166278	-62.884			PQ605847	PV617866		PV632708					PV633506			
329_Rbre	329	pictipes	*R.brethesi*	Brasil	Amazonas	Barcelos	-0.97166278	-62.884				PV617956		PV632709					PV633518		PV632407	
331_Rbre	331	pictipes	*R.brethesi*	Brasil	Amazonas	Barcelos	-0.97166278	-62.884				PV617958		PV632508					PV633666		PV632232	
373_Rbre	373	pictipes	*R.brethesi*	Brasil	Amazonas	Barcelos	-0.97166278	-62.884						PV632513								
374_Rbre	374	pictipes	*R.brethesi*	Brasil	Amazonas	Barcelos	-0.97166278	-62.884						PV632770					PV633489		PV632252	
376_Rbre	376	pictipes	*R.brethesi*	Brasil	Amazonas	Barcelos	-0.97166278	-62.884			PQ605854	PV617869		PV632657							PV632253	
378_Rbre	378	pictipes	*R.brethesi*	Brasil	Amazonas	Barcelos	-0.97166278	-62.884				PV617968		PV632797					PV633457		PV632254	
380_Rbre	380	pictipes	*R.brethesi*	Brasil	Amazonas	Barcelos	-0.97166278	-62.884			PQ605855	PV617862		PV632514					PV633405		PV632255	
381_Rbre	381	pictipes	*R.brethesi*	Brasil	Amazonas	Barcelos	-0.97166278	-62.884						PV632515					PV633547		PV632256	
382_Rbre	382	pictipes	*R.brethesi*	Brasil	Amazonas	Barcelos	-0.97166278	-62.884				PV617970										
41_Rcol	41	pallescens	*R.colombiensis*	Colombia	Tolima	Coyaima	3.79694	-75.1946				PV617785			PV633233		PV632853		PV633555		PV632118	
42_Rcol	42	pallescens	*R.colombiensis*	Colombia	Tolima	Coyaima	3.79694	-75.1946				PV617786		PV632717	PV633234		PV632899		PV633525		PV632401	
43_Rcol	43	pallescens	*R.colombiensis*	Colombia	Tolima	Coyaima	3.79694	-75.1946				PV617787			PV633235				PV633342		PV632119	
63_Rcol	63	pallescens	*R.colombiensis*	Colombia	Tolima	Coyaima	3.79694	-75.1946			PQ605931	PV617897		PV632721	PV633139		PV632898		PV633343		PV632120	
64_Rcol	64	pallescens	*R.colombiensis*	Colombia	Tolima	Coyaima	3.79694	-75.1946			PQ605932	PV617895		PV632720	PV633140		PV632896		PV633344		PV632121	
65_Rcol	65	pallescens	*R.colombiensis*	Colombia	Tolima	Coyaima	3.79694	-75.1946						PV632719	PV633167		PV632897					
66_Rcol	66	pallescens	*R.colombiensis*	Colombia	Tolima	Coyaima	3.79694	-75.1946				PV617788		PV632718	PV633164		PV633076		PV633356		PV632122	
67_Rcol	67	pallescens	*R.colombiensis*	Colombia	Tolima	Coyaima	3.79694	-75.1946			PQ605933	PV617789		PV632748	PV633141		PV633077		PV633345		PV632123	
383_Rcol	383	pallescens	*R.colombiensis*	Colombia	Tolima	Coyaima	3.79694	-75.1946	PQ585906		PQ605856	PV617823									PV632257	
385_Rcol	385	pallescens	*R.colombiensis*	Colombia	Tolima	Coyaima	3.79694	-75.1946			PQ605949	PV617824							PV633364		PV632258	
387_Rcol	387	pallescens	*R.colombiensis*	Colombia	Tolima	Coyaima	3.79694	-75.1946	PQ585886		PQ605867	PV617825									PV632259	
399_Rcol	399	pallescens	*R.colombiensis*	Colombia	Tolima	Coyaima	3.79694	-75.1946							PV633144				PV633466		PV632276	
400_Rcol	400	pallescens	*R.colombiensis*	Colombia	Tolima	Coyaima	3.79694	-75.1946									PV632851					
401_Rcol	401	pallescens	*R.colombiensis*	Colombia	Tolima	Coyaima	3.79694	-75.1946				PV617827		PV632785	PV633163				PV633660			
270_Recu	270	pallescens	*R.ecuadoriensis*	Ecuador			-1.831239	-78.183406	PQ585879		PQ605831			PV632624	PV633127				PV633567		PV632201	
271_Recu	271	pallescens	*R.ecuadoriensis*	Ecuador			-1.831239	-78.183406				PV617943										
272_Recu	272	pallescens	*R.ecuadoriensis*	Ecuador			-1.831239	-78.183406	PQ585852		PQ605832			PV632625			PV633072		PV633455		PV632202	
273_Recu	273	pallescens	*R.ecuadoriensis*	Ecuador			-1.831239	-78.183406				PV618137	PV618138									
274_Recu	274	pallescens	*R.ecuadoriensis*	Ecuador			-1.831239	-78.183406	PQ585880		PQ605833	PV617914		PV632626	PV633172				PV633536		PV632203	
276_Recu	276	pallescens	*R.ecuadoriensis*	Ecuador			-1.831239	-78.183406	PQ585881		PQ605834	PV618029	PV618030	PV632627	PV633191		PV632915		PV633683	PV633684		
278_Recu	278	pallescens	*R.ecuadoriensis*	Ecuador			-1.831239	-78.183406	PQ585861		PQ605835	PV617805		PV632628	PV633182		PV633048		PV633513		PV632204	
413_Recu	413	pallescens	*R.ecuadoriensis*	Ecuador	Manabi	Portoviejo	-1.0561	-80.4552				PV617870		PV632516			PV632916					
414_Recu	414	pallescens	*R.ecuadoriensis*	Ecuador	Manabi	Portoviejo	-1.0561	-80.4552			PQ605861			PV632517							PV632281	
415_Recu	415	pallescens	*R.ecuadoriensis*	Ecuador	Manabi	Portoviejo	-1.0561	-80.4552			PQ605862						PV632917				PV632314	
416_Recu	416	pallescens	*R.ecuadoriensis*	Ecuador	Manabi	Portoviejo	-1.0561	-80.4552	PQ586006										PV633358			
417_Recu	417	pallescens	*R.ecuadoriensis*	Ecuador	Manabi	Portoviejo	-1.0561	-80.4552			PQ605863	PV617832		PV632518			PV633115	PV633116	PV633359		PV632260	
419_Recu	419	pallescens	*R.ecuadoriensis*	Ecuador	Manabi	Portoviejo	-1.0561	-80.4552			PQ605864	PV618071	PV618072	PV632654								
420_Recu	420	pallescens	*R.ecuadoriensis*	Ecuador	Manabi	Portoviejo	-1.0561	-80.4552							PV633324				PV633693	PV633694		
421_Recu	421	pallescens	*R.ecuadoriensis*	Ecuador	Manabi	Portoviejo	-1.0561	-80.4552				PV617833		PV632655								
422_Recu	422	pallescens	*R.ecuadoriensis*	Ecuador	Manabi	Portoviejo	-1.0561	-80.4552			PQ605865			PV632757			PV633054		PV633695	PV633696	PV632261	
544_Recu	544	pallescens	*R.ecuadoriensis*	Ecuador	Manabi	San Gregorio	-3.355408	-80.284035									PV632921	PV632922				
545_Recu	545	pallescens	*R.ecuadoriensis*	Ecuador	Manabi	San Gregorio	-3.355408	-80.284035				PV617845		PV632786								
546_Recu	546	pallescens	*R.ecuadoriensis*	Ecuador	Loja	La cienaga	-3.99313	-79.2042						PV632676			PV632923		PV633351		PV632315	
548_Recu	548	pallescens	*R.ecuadoriensis*	Ecuador	Loja	Bramaderos	-4000	-79.81						PV632677			PV632952				PV632316	
549_Recu	549	pallescens	*R.ecuadoriensis*	Ecuador	Loja	Bramaderos	-4000	-79.81				PV617867										
550_Recu	550	pallescens	*R.ecuadoriensis*	Ecuador	Manabi	Cruz Alta	-28.64397	-53.6063													PV632317	
592_Recu	592	pallescens	*R.ecuadoriensis*	Ecuador	Manabi	Naranjo Adentro	0.59318	-77.8307				PV617851							PV633672			
595_Recu	595	pallescens	*R.ecuadoriensis*	Ecuador	Manabi	San Gabriel	0.59318	-77.8307									PV632924				PV632331	
597_Recu	597	pallescens	*R.ecuadoriensis*	Ecuador	Loja	La Extensa	-3.99313	-79.2042									PV632925					
599_Recu	599	pallescens	*R.ecuadoriensis*	Ecuador	Loja	La Extensa	-3.99313	-79.2042	PQ586042								PV632926				PV632425	
600_Recu	600	pallescens	*R.ecuadoriensis*	Ecuador	Loja	La Extensa	-3.99313	-79.2042											PV633483			
601_Recu	601	pallescens	*R.ecuadoriensis*	Ecuador	Loja	San Francisco	0.6543025	-80.066065						PV632787								
602_Recu	602	pallescens	*R.ecuadoriensis*	Ecuador	Loja	San Francisco	0.6543025	-80.066065			PQ605955	PV617878							PV633436			
453_Rmar	453	prolixus	*R.marabaensis*	Brasil			-5.38146	-49.13232	PQ586075	PQ586076	PQ605873	PV617962			PV633178		PV633113	PV633114	PV633452			
454_Rmar	454	prolixus	*R.marabaensis*	Brasil			-5.38146	-49.13232							PV633171		PV632895		PV633510		PV632269	
455_Rmar	455	prolixus	*R.marabaensis*	Brasil			-5.38146	-49.13232				PV617837		PV632673								
456_Rmar	456	prolixus	*R.marabaensis*	Brasil			-5.38146	-49.13232	PQ586031					PV632759	PV633201		PV632997	PV632998	PV633665		PV632270	
458_Rmar	458	prolixus	*R.marabaensis*	Brasil			-5.38146	-49.13232	PQ585860					PV632666			PV633020		PV633546		PV632271	
294_Rmil	294	prolixus	*R.milesi*	Brasil	Para	Braganca	-22.95	-46.54			PQ605839								PV633361		PV632219	
295_Rmil	295	prolixus	*R.milesi*	Brasil	Para	Braganca	-22.95	-46.54	PQ585947		PQ605840			PV632727	PV633169				PV633512		PV632220	
296_Rmil	296	prolixus	*R.milesi*	Brasil	Para	Braganca	-22.95	-46.54				PV618133	PV618134									
297_Rmil	297	prolixus	*R.milesi*	Brasil	Para	Braganca	-22.95	-46.54											PV633647		PV632221	
298_Rmil	298	prolixus	*R.milesi*	Brasil	Para	Braganca	-22.95	-46.54	PQ585969		PQ605841	PV617886			PV633315		PV632864		PV633362		PV632222	
299_Rmil	299	prolixus	*R.milesi*	Brasil	Para	Braganca	-22.95	-46.54	PQ585995		PQ605842	PV617887		PV632728	PV633173				PV633727	PV633728	PV632223	
301_Rmil	301	prolixus	*R.milesi*	Brasil	Para	Braganca	-22.95	-46.54	PQ585893		PQ605843			PV632729			PV632866		PV633391		PV632224	
303_Rmil	303	prolixus	*R.milesi*	Brasil	Para	Braganca	-22.95	-46.54	PQ585929		PQ605844			PV632802			PV632867		PV633648		PV632410	
304_Rmil	304	prolixus	*R.milesi*	Brasil	Para	Braganca	-22.95	-46.54				PV618031	PV618032									
305_Rmil	305	prolixus	*R.milesi*	Brasil	Para	Braganca	-22.95	-46.54	PQ585874					PV632730	PV633206				PV633649		PV632209	
306_Rmil	306	prolixus	*R.milesi*	Brasil	Para	Braganca	-22.95	-46.54				PV618033	PV618034									

460_Rmil	460	prolixus	*R.milesi*	Brasil	Para	Braganca	-22.95	-46.54	PQ586018								PV632883		PV633394		PV632272	
462_Rmil	462	prolixus	*R.milesi*	Brasil	Para	Braganca	-22.95	-46.54	PQ585996								PV632884					
464_Rmil	464	prolixus	*R.milesi*	Brasil	Para	Braganca	-22.95	-46.54	PQ585974								PV632958		PV633516		PV632273	
465_Rmil	465	prolixus	*R.milesi*	Brasil	Para	Braganca	-22.95	-46.54						PV632780								
466_Rmil	466	prolixus	*R.milesi*	Brasil	Para	Braganca	-22.95	-46.54				PV617838					PV633070		PV633653		PV632283	
467_Rmil	467	prolixus	*R.milesi*	Brasil	Para	Braganca	-22.95	-46.54	PQ585953		PQ605875	PV618051	PV618052						PV633651		PV632398	
119_Rmon	119	prolixus	*R.montenegrensis*	Brasil	Rondonia	Monte Negro	-29.68	-51.46	PQ585957		PQ606018	PV618141	PV618142	PV632555	PV633333	PV633334			PV633631		PV632172	
120_Rmon	120	prolixus	*R.montenegrensis*	Brasil	Rondonia	Monte Negro	-29.68	-51.46			PQ605820	PV618145	PV618146	PV632556	PV633278		PV633092		PV633677	PV633678	PV632397	
121_Rmon	121	prolixus	*R.montenegrensis*	Brasil	Rondonia	Monte Negro	-29.68	-51.46	PQ585990		PQ605998	PV618147	PV618148	PV632557	PV633335	PV633336	PV632986				PV632384	
122_Rmon	122	prolixus	*R.montenegrensis*	Brasil	Rondonia	Monte Negro	-29.68	-51.46				PV618149	PV618150	PV632558	PV633325	PV633326	PV632979	PV632980	PV633721	PV633722	PV632227	
124_Rmon	124	prolixus	*R.montenegrensis*	Brasil	Rondonia	Monte Negro	-29.68	-51.46			PQ605918	PV618151	PV618152	PV632559	PV633228		PV633041		PV633606		PV632443	
125_Rmon	125	prolixus	*R.montenegrensis*	Brasil	Rondonia	Monte Negro	-29.68	-51.46	PQ585975		PQ605981	PV618127	PV618128	PV632744	PV633283		PV633086		PV633719	PV633720	PV632406	
127_Rmon	127	prolixus	*R.montenegrensis*	Brasil	Rondonia	Monte Negro	-29.68	-51.46				PV618193	PV618194	PV632561	PV633280				PV633715	PV633716	PV632409	
128_Rmon	128	prolixus	*R.montenegrensis*	Brasil	Rondonia	Monte Negro	-29.68	-51.46	PQ585948		PQ605983	PV618002		PV632562	PV633281		PV633119	PV633120	PV633645		PV632430	
469_Rmon	469	prolixus	*R.montenegrensis*	Brasil	Rondonia	Monte Negro	-29.68	-51.46	PQ585954			PV618085	PV618086	PV632776	PV633210		PV633111	PV633112	PV633685	PV633686		
471_Rmon	471	prolixus	*R.montenegrensis*	Brasil	Rondonia	Monte Negro	-29.68	-51.46	PQ586067	PQ586068		PV618081	PV618082	PV632772	PV633194		PV633121	PV633122	PV633652		PV632284	
474_Rmon	474	prolixus	*R.montenegrensis*	Brasil	Rondonia	Monte Negro	-29.68	-51.46			PQ605876			PV63279					PV633438			
475_Rmon	475	prolixus	*R.montenegrensis*	Brasil	Rondonia	Monte Negro	-29.68	-51.46						PV632777	PV633195		PV633101	PV633102	PV633403		PV632285	
476_Rmon	476	prolixus	*R.montenegrensis*	Brasil	Rondonia	Monte Negro	-29.68	-51.46			PQ605877											
478_Rmon	478	prolixus	*R.montenegrensis*	Brasil	Rondonia	Monte Negro	-29.68	-51.46				PV617963		PV632760	PV633202		PV633123	PV633124	PV633697	PV633698	PV632286	
110_Rnas	110	prolixus	*R.nasutus*	Brasil	Ceara	Varzea Alegre	-9.96	-48.754	PQ585960		PQ606020	PV618010		PV632742			PV632981		PV633626		PV632403	
111_Rnas	111	prolixus	*R.nasutus*	Brasil	Ceara	Varzea Alegre	-9.96	-48.754			PQ606007	PV618103	PV618104	PV632549					PV633627		PV632389	
112_Rnas	112	prolixus	*R.nasutus*	Brasil	Ceara	Varzea Alegre	-9.96	-48.754	PQ585888		PQ606016			PV632743					PV633604		PV632390	
113_Rnas	113	prolixus	*R.nasutus*	Brasil	Ceara	Varzea Alegre	-9.96	-48.754	PQ585907					PV632550			PV633040		PV633640		PV632391	
114_Rnas	114	prolixus	*R.nasutus*	Brasil	Ceara	Varzea Alegre	-9.96	-48.754	PQ585908		PQ606021			PV632551			PV632967		PV633628			
115_Rnas	115	prolixus	*R.nasutus*	Brasil	Ceara	Varzea Alegre	-9.96	-48.754			PQ605816			PV632552			PV632978		PV633629		PV632160	
116_Rnas	116	prolixus	*R.nasutus*	Brasil	Ceara	Varzea Alegre	-9.96	-48.754			PQ605817	PV618005		PV632553					PV633634		PV632404	
117_Rnas	117	prolixus	*R.nasutus*	Brasil	Ceara	Varzea Alegre	-9.96	-48.754	PQ586069	PQ586070	PQ605818	PV618007		PV632496					PV633630		PV632441	
118_Rnas	118	prolixus	*R.nasutus*	Brasil	Ceara	Varzea Alegre	-9.96	-48.754			PQ605819	PV618009		PV632554			PV633107	PV633108	PV633605		PV632432	
85_Rneg	85	prolixus	*R.neglectus*	Brasil	Goias	Formoso	-14.9505	-46.2348						PV632601			PV633005		PV633610		PV632150	
86_Rneg	86	prolixus	*R.neglectus*	Brasil	Goias	Formoso	-14.9505	-46.2348						PV632602			PV633006		PV633618		PV632151	
87_Rneg	87	prolixus	*R.neglectus*	Brasil	Goias	Formoso	-14.9505	-46.2348						PV632603			PV633052		PV633574		PV632152	
88_Rneg	88	prolixus	*R.neglectus*	Brasil	Goias	Formoso	-14.9505	-46.2348						PV632762			PV633053		PV633619		PV632153	
89_Rneg	89	prolixus	*R.neglectus*	Brasil	Goias	Formoso	-14.9505	-46.2348						PV632534					PV633592		PV632229	
90_Rneg	90	prolixus	*R.neglectus*	Brasil	Goias	Formoso	-14.9505	-46.2348						PV632535			PV633061		PV633620		PV632228	
93_Rneg	93	prolixus	*R.neglectus*	Brasil	Goias	Formoso	-14.9505	-46.2348						PV632536	PV633319		PV632975		PV633621		PV632154	
94_Rneg	94	prolixus	*R.neglectus*	Brasil	Goias	Formoso	-14.9505	-46.2348			PQ605969			PV632741	PV633320				PV633622		PV632155	
95_Rneg	95	prolixus	*R.neglectus*	Brasil	Goias	Formoso	-14.9505	-46.2348			PQ606014			PV632537	PV633321		PV633088				PV632156	
96_Rneg	96	prolixus	*R.neglectus*	Brasil	Goias	Formoso	-14.9505	-46.2348			PQ606011			PV632538					PV633594		PV632157	
354_Rneg	354	prolixus	*R.neglectus*	Brasil	Minas Gerais	Uberaba	-19.7502	-47.9325				PV617964		PV632510	PV633190							
355_Rneg	355	prolixus	*R.neglectus*	Brasil	Minas Gerais	Uberaba	-19.7502	-47.9325	PQ586057	PQ586058				PV632754					PV633385		PV632245	
357_Rneg	357	prolixus	*R.neglectus*	Brasil	Minas Gerais	Uberaba	-19.7502	-47.9325	PQ586059	PQ586060	PQ605851			PV632755			PV632876		PV633363		PV632246	
359_Rneg	359	prolixus	*R.neglectus*	Brasil	Minas Gerais	Uberaba	-19.7502	-47.9325	PQ586065	PQ586066	PQ605852			PV632511	PV633176		PV633027		PV633490		PV632247	
362_Rneg	362	prolixus	*R.neglectus*	Brasil	Minas Gerais	Uberaba	-19.7502	-47.9325				PV618069	PV618070									
363_Rneg	363	prolixus	*R.neglectus*	Brasil	Minas Gerais	Uberaba	-19.7502	-47.9325	PQ585952					PV632731			PV633029		PV633725	PV633726		
365_Rneg	365	prolixus	*R.neglectus*	Brasil	Minas Gerais	Uberaba	-19.7502	-47.9325									PV632877	PV632878			PV632249	
366_Rneg	366	prolixus	*R.neglectus*	Brasil	Minas Gerais	Uberaba	-19.7502	-47.9325														
367_Rneg	367	prolixus	*R.neglectus*	Brasil	Minas Gerais	Uberaba	-19.7502	-47.9325			PQ605853						PV632879		PV633386		PV632463	PV632464
368_Rneg	368	prolixus	*R.neglectus*	Brasil	Minas Gerais	Uberaba	-19.7502	-47.9325				PV617821		PV632756								
369_Rneg	369	prolixus	*R.neglectus*	Brasil	Minas Gerais	Uberaba	-19.7502	-47.9325	PQ585930										PV633379		PV632282	
370_Rneg	370	prolixus	*R.neglectus*	Brasil	Minas Gerais	Uberaba	-19.7502	-47.9325				PV617822		PV632734								
371_Rneg	371	prolixus	*R.neglectus*	Brasil	Minas Gerais	Uberaba	-19.7502	-47.9325	PQ585978								PV632880		PV633482		PV632251	
372_Rneg	372	prolixus	*R.neglectus*	Brasil	Minas Gerais	Uberaba	-19.7502	-47.9325				PV618039	PV618040									
375_Rneg	375	prolixus	*R.neglectus*	Brasil	Minas Gerais	Uberaba	-19.7502	-47.9325	PQ586035					PV632656					PV633352			
403_Rneg	403	prolixus	*R.neglectus*	Brasil	Tocantins	Taquarussu	-22.4886	-53.3519						PV632732								
404_Rneg	404	prolixus	*R.neglectus*	Brasil	Tocantins	Taquarussu	-22.4886	-53.3519			PQ605857	PV617828			PV633302				PV633657			
405_Rneg	405	prolixus	*R.neglectus*	Brasil	Tocantins	Taquarussu	-22.4886	-53.3519						PV632740					PV633380		PV632277	
406_Rneg	406	prolixus	*R.neglectus*	Brasil	Tocantins	Taquarussu	-22.4886	-53.3519				PV617829					PV632881					
407_Rneg	407	prolixus	*R.neglectus*	Brasil	Tocantins	Taquarussu	-22.4886	-53.3519			PQ605858			PV632773					PV633517		PV632278	
409_Rneg	409	prolixus	*R.neglectus*	Brasil	Tocantins	Taquarussu	-22.4886	-53.3519	PQ585894		PQ605859	PV617830		PV632733	PV633155		PV633030		PV633390		PV632279	
411_Rneg	411	prolixus	*R.neglectus*	Brasil	Tocantins	Taquarussu	-22.4886	-53.3519			PQ605860	PV617831		PV632799	PV633183		PV632882		PV633492		PV632280	
479_Rnei	479	prolixus	*R.neivai*	Venezuela	Valencia	ND	-29.68	-51.46	PQ586051	PQ586052	PQ605878				PV633147		PV632815		PV633502		PV632287	
481_Rnei	481	prolixus	*R.neivai*	Venezuela	Valencia	ND	-29.68	-51.46	PQ585979			PV617839					PV632816		PV633443		PV632288	
483_Rnei	483	prolixus	*R.neivai*	Venezuela	Valencia	ND	-29.68	-51.46	PQ585961								PV632817		PV633444		PV632289	
485_Rnei	485	prolixus	*R.neivai*	Venezuela	Valencia	ND	-29.68	-51.46	PQ586036													
486_Rnei	486	prolixus	*R.neivai*	Venezuela	Valencia	ND	-29.68	-51.46			PQ605879						PV632818		PV633445		PV632290	
487_Rnei	487	prolixus	*R.neivai*	Venezuela	Valencia	ND	-29.68	-51.46									PV632819		PV633446		PV632291	
488_Rnei	488	prolixus	*R.neivai*	Venezuela	Valencia	ND	-29.68	-51.46				PV617840										
777_Rnei	777	prolixus	*R.neivai*	Colombia	Boyaca	Cubara	7.033	-72.067														
781_Rnei	781	prolixus	*R.neivai*	Colombia	Boyaca	Cubara	7.033	-72.067	PQ586011			PV617971		PV632749			PV632820		PV633486	PV633711	PV632380	
14_Rpal	14	pallescens	*R.pallescens*	Colombia	Antioquia	Necocli	8.4357	-76.7767				PV617998		PV632527					PV633347		PV632129	
58_Rpal	58	pallescens	*R.pallescens*	Colombia	Antioquia	Necocli	8.4357	-76.7767				PV617790		PV632726	PV633142		PV632900		PV633556		PV632124	
59_Rpal	59	pallescens	*R.pallescens*	Colombia	Antioquia	Necocli	8.4357	-76.7767				PV617893		PV632725	PV633143		PV632901		PV633557		PV632125	
60_Rpal	60	pallescens	*R.pallescens*	Colombia	Antioquia	Necocli	8.4357	-76.7767			PQ605972	PV617898		PV632724	PV633236		PV632854		PV633583		PV632126	
61_Rpal	61	pallescens	*R.pallescens*	Colombia	Antioquia	Necocli	8.4357	-76.7767			PQ605973	PV617791		PV632723	PV633241		PV632902		PV633365		PV632127	
62_Rpal	62	pallescens	*R.pallescens*	Colombia	Antioquia	Necocli	8.4357	-76.7767				PV618000		PV632722	PV633242		PV632855		PV633346		PV632128	
132_Rpal	132	pallescens	*R.pallescens*	Colombia	Cesar	Valledupar	10.46314	-73.25322	PQ585921		PQ606010	PV618006		PV632635			PV633074		PV633582		PV632435	
133_Rpal	133	pallescens	*R.pallescens*	Colombia	Cesar	Valledupar	10.46314	-73.25322	PQ585914			PV618163	PV618164	PV632636					PV633526		PV632451	PV632452
135_Rpal	135	pallescens	*R.pallescens*	Colombia	Cesar	Valledupar	10.46314	-73.25322	PQ585925			PV617986	PV617987	PV632637								
136_Rpal	136	pallescens	*R.pallescens*	Colombia	Cesar	Valledupar	10.46314	-73.25322	PQ585915		PQ605977			PV632737			PV632959		PV633462			
137_Rpal	137	pallescens	*R.pallescens*	Colombia	Cesar	Valledupar	10.46314	-73.25322	PQ585916		PQ605980	PV618165	PV618166	PV632638			PV632906		PV633589		PV632162	
138_Rpal	138	pallescens	*R.pallescens*	Colombia	Cesar	Valledupar	10.46314	-73.25322				PV618167	PV618168	PV632639							PV632163	
139_Rpal	139	pallescens	*R.pallescens*	Colombia	Cesar	Valledupar	10.46314	-73.25322			PQ606008	PV618079	PV618080	PV632640			PV632905		PV633417		PV632437	
140_Rpal	140	pallescens	*R.pallescens*	Colombia	Cesar	Valledupar	10.46314	-73.25322	PQ585917		PQ605999	PV617978		PV632641					PV633669		PV632161	

141_Rpal	141	pallescens	*R.pallescens*	Colombia	Cesar	Valledupar	10.46314	-73.25322	PQ585918			PV617944		PV632642			PV633079		PV633463		PV632420	
144_Rpal	144	pallescens	*R.pallescens*	Colombia	Antioquia	Necocli	8.4357	-76.7767	PQ586005													
146_Rpal	146	pallescens	*R.pallescens*	Colombia	Antioquia	Necocli	8.4357	-76.7767				PV618089	PV618090	PV632643			PV633081		PV633528		PV632095	
149_Rpal	149	pallescens	*R.pallescens*	Colombia	Antioquia	Necocli	8.4357	-76.7767				PV618125	PV618126	PV632644			PV633068				PV632449	PV632450
150_Rpal	150	pallescens	*R.pallescens*	Colombia	Antioquia	Necocli	8.4357	-76.7767				PV618169	PV618170	PV632645					PV633467		PV632164	
151_Rpal	151	pallescens	*R.pallescens*	Colombia	Antioquia	Necocli	8.4357	-76.7767				PV618131	PV618132	PV632646					PV633459		PV632416	
152_Rpal	152	pallescens	*R.pallescens*	Colombia	Antioquia	Necocli	8.4357	-76.7767	PQ585980			PV618173	PV618174	PV632523					PV633523		PV632394	
153_Rpal	153	pallescens	*R.pallescens*	Colombia	Antioquia	Necocli	8.4357	-76.7767				PV618175	PV618176								PV632417	
169_Rpal	169	pallescens	*R.pallescens*	Colombia	Antioquia	Necocli	8.4357	-76.7767	PQ585993			PV618177	PV618178	PV632647					PV633424		PV632479	PV632480
174_Rpal	174	pallescens	*R.pallescens*	Colombia	Antioquia	Necocli	8.4357	-76.7767				PV617992		PV632648			PV632907		PV633370			
179_Rpal	179	pallescens	*R.pallescens*	Colombia	Meta	Restrepo	4.26211	-73.5649				PV618179	PV618180	PV632650					PV633575			
185_Rpal	185	pallescens	*R.pallescens*	Colombia	Bolivar	Mompox	9.233	-74.417			PQ606005	PV617995							PV633425		PV632438	
186_Rpal	186	pallescens	*R.pallescens*	Colombia	Bolivar	Mompox	9.233	-74.417			PQ605993	PV618187	PV618188	PV632651							PV632096	
187_Rpal	187	pallescens	*R.pallescens*	Colombia	Bolivar	Mompox	9.233	-74.417				PV617993		PV632652							PV632097	PV632098
188_Rpal	188	pallescens	*R.pallescens*	Colombia	Bolivar	Mompox	9.233	-74.417				PV618161	PV618162	PV632653							PV632447	PV632448
235_Rpal	235	pallescens	*R.pallescens*	Panam	Chorrera	Las Pavas	9.09974	-79.889678	PQ585984			PV618015	PV618016	PV632798			PV632908		PV633430		PV632453	PV632454
237_Rpal	237	pallescens	*R.pallescens*	Panam	Chorrera	Las Pavas	9.09974	-79.889678	PQ586014			PV618017	PV618018	PV632525	PV633168		PV632909		PV633580		PV632182	
239_Rpal	239	pallescens	*R.pallescens*	Panam	Chorrera	Las Pavas	9.09974	-79.889678				PV618019	PV618020	PV632526			PV632910		PV633566		PV632183	
241_Rpal	241	pallescens	*R.pallescens*	Panam	Chorrera	Las Pavas	9.09974	-79.889678			PQ605824	PV618021	PV618022	PV632750			PV632949		PV633530		PV632184	
243_Rpal	243	pallescens	*R.pallescens*	Panam	Darien	Meteti	8.5	-77.97						PV632789								
245_Rpal	245	pallescens	*R.pallescens*	Panam	Darien	Meteti	8.5	-77.97			PQ605825	PV617796		PV632634					PV633531		PV632185	
247_Rpal	247	pallescens	*R.pallescens*	Panam	Darien	Meteti	8.5	-77.97				PV618023	PV618024	PV632796			PV632911		PV633389		PV632186	
249_Rpal	249	pallescens	*R.pallescens*	Panam	Darien	Meteti	8.5	-77.97				PV617797			PV633313						PV632455	PV632456
251_Rpal	251	pallescens	*R.pallescens*	Panam	Chorrera	Pma.Oeste	8.88028	-79.7833	PQ585959		PQ605826	PV618025	PV618026	PV632745					PV633393		PV632187	
253_Rpal	253	pallescens	*R.pallescens*	Panam	Chorrera	Pma.Oeste	8.88028	-79.7833			PQ605827	PV617798		PV632795					PV633494		PV632188	
255_Rpal	255	pallescens	*R.pallescens*	Panam	Chorrera	Pma.Oeste	8.88028	-79.7833	PQ585905			PV617799			PV633268		PV632912		PV633568		PV632457	PV632458
257_Rpal	257	pallescens	*R.pallescens*	Panam	Chorrera	Pma.Oeste	8.88028	-79.7833	PQ585962			PV618027	PV618028	PV632619			PV632913		PV633581		PV632189	
259_Rpal	259	pallescens	*R.pallescens*	Panam	Chorrera	Pma.Oeste	8.88028	-79.7833			PQ605828	PV617800		PV632620			PV632914		PV633532		PV632190	
260_Rpal	260	pallescens	*R.pallescens*	Panam	Panam	Chilibre	9.15093	-79.62098				PV617801							PV633533		PV632191	
262_Rpal	262	pallescens	*R.pallescens*	Panam	Panam	Chilibre	9.15093	-79.62098				PV617802		PV632621					PV633534		PV632192	
263_Rpal	263	pallescens	*R.pallescens*	Panam	Panam	Chilibre	9.15093	-79.62098	PQ585992		PQ605829	PV617803		PV632622					PV633535		PV632194	
265_Rpal	265	pallescens	*R.pallescens*	Panam	Panam	Chilibre	9.15093	-79.62098						PV632623							PV632225	
266_Rpal	266	pallescens	*R.pallescens*	Panam	Panam	Chilibre	9.15093	-79.62098	PQ586048		PQ605830			PV632736					PV633644		PV632226	
269_Rpal	269	pallescens	*R.pallescens*	Panam	Panam	Chilibre	9.15093	-79.62098				PV617804									PV632200	
423_Rpal	423	pallescens	*R.pallescens*	Colombia	Bolivar	San Fernando	9.283	-74.533			PQ605866	PV618083	PV618084	PV632658					PV633545			
424_Rpal	424	pallescens	*R.pallescens*	Colombia	Bolivar	San Fernando	9.283	-74.533				PV617876		PV632793			PV632963		PV633507			
426_Rpal	426	pallescens	*R.pallescens*	Colombia	Bolivar	San Fernando	9.283	-74.533			PQ605952	PV617871		PV632758	PV633126		PV633014					
428_Rpal	428	pallescens	*R.pallescens*	Colombia	Bolivar	San Fernando	9.283	-74.533				PV618129	PV618130	PV632659			PV632950		PV633451			
429_Rpal	429	pallescens	*R.pallescens*	Colombia	Bolivar	San Fernando	9.283	-74.533	PQ586023		PQ605953	PV617877		PV632794					PV633544			
726_Rpal	726	pallescens	*R.pallescens*	Colombia	Santander	Bucaramanga	7.12539	-73.1198	PQ586039			PV617861		PV632792					PV633484			
751_Rpal	751	pallescens	*R.pallescens*	Colombia	C¢rdoba	Monteria	8.75	-75.883				PV618119	PV618120	PV632790					PV633674		PV632477	PV632478
178_Rpic	178	pictipes	*R.pictipes*	Colombia	Meta	Restrepo	4.26211	-73.5649						PV632649					PV633689	PV633690		
182_Rpic	182	pictipes	*R.pictipes*	Colombia	Meta	Restrepo	4.26211	-73.5649				PV617984		PV632764					PV633709	PV633710		
183_Rpic	183	pictipes	*R.pictipes*	Colombia	Meta	Restrepo	4.26211	-73.5649				PV618183	PV618184						PV633578			
388_Rpic	388	pictipes	*R.pictipes*	Brasil	Para	Maraba	-5.368888889	-49.11777778				PV618041	PV618042						PV633670		PV632465	PV632466
390_Rpic	390	pictipes	*R.pictipes*	Brasil	Para	Maraba	-5.368888889	-49.11777778	PQ585985			PV618043	PV618044						PV633691	PV633692		
391_Rpic	391	pictipes	*R.pictipes*	Brasil	Para	Maraba	-5.368888889	-49.11777778													PV632274	
394_Rpic	394	pictipes	*R.pictipes*	Brasil	Para	Maraba	-5.368888889	-49.11777778			PQ606003	PV617826							PV633464		PV632275	
396_Rpic	396	pictipes	*R.pictipes*	Brasil	Para	Maraba	-5.368888889	-49.11777778											PV633465		PV632467	PV632468
1_Rpro	1	prolixus	*R.prolixus*	Colombia	Casanare	Mani	4.818055556	-72.28111111				PV617773		PV632618			PV632955		PV633584		PV632101	
2_Rpro	2	prolixus	*R.prolixus*	Colombia	Casanare	Mani	4.818055556	-72.28111111				PV617774			PV633128				PV633369		PV632107	
3_Rpro	3	prolixus	*R.prolixus*	Colombia	Casanare	Mani	4.818055556	-72.28111111				PV617775			PV633129		PV632961		PV633553		PV632108	
4_Rpro	4	prolixus	*R.prolixus*	Colombia	Casanare	Mani	4.818055556	-72.28111111				PV617776		PV632617			PV632857		PV633585		PV632382	
5_Rpro	5	prolixus	*R.prolixus*	Colombia	Casanare	Mani	4.818055556	-72.28111111						PV632616	PV633217				PV633524		PV632135	
6_Rpro	6	prolixus	*R.prolixus*	Colombia	Casanare	Mani	4.818055556	-72.28111111				PV617777									PV632109	
7_Rpro	7	prolixus	*R.prolixus*	Colombia	Casanare	Mani	4.818055556	-72.28111111						PV632615	PV633239		PV632957		PV633415		PV632102	
8_Rpro	8	prolixus	*R.prolixus*	Colombia	Casanare	Mani	4.818055556	-72.28111111				PV617996			PV633216		PV632892				PV632136	
9_Rpro	9	prolixus	*R.prolixus*	Colombia	Casanare	Mani	4.818055556	-72.28111111			PQ605968	PV617778			PV633158		PV632858				PV632103	
10_Rpro	10	prolixus	*R.prolixus*	Colombia	Casanare	Mani	4.818055556	-72.28111111				PV617779		PV632614	PV633240		PV633032		PV633338		PV632137	
11_Rpro	11	prolixus	*R.prolixus*	Colombia	Casanare	Mani	4.818055556	-72.28111111				PV617780		PV632613	PV633130				PV633339		PV632104	
12_Rpro	12	prolixus	*R.prolixus*	Colombia	Casanare	Mani	4.818055556	-72.28111111			PQ606004			PV632612	PV633137		PV633002				PV632105	
13_Rpro	13	prolixus	*R.prolixus*	Colombia	Casanare	Mani	4.818055556	-72.28111111			PQ605963	PV618008		PV632611			PV632956		PV633552		PV632106	
16_Rpro	16	prolixus	*R.prolixus*	Colombia	Casanare	Villanueva	4.608736	-72.928792			PQ605944			PV632610	PV633292							
17_Rpro	17	prolixus	*R.prolixus*	Colombia	Casanare	Villanueva	4.608736	-72.928792						PV632609	PV633284							
18_Rpro	18	prolixus	*R.prolixus*	Colombia	Casanare	Villanueva	4.608736	-72.928792			PQ605967				PV633131							
19_Rpro	19	prolixus	*R.prolixus*	Colombia	Casanare	Villanueva	4.608736	-72.928792			PQ605943											
21_Rpro	21	prolixus	*R.prolixus*	Colombia	Cesar	Valledupar	10.46314	-73.25322						PV632608								
22_Rpro	22	prolixus	*R.prolixus*	Colombia	Casanare	Villanueva	4.608736	-72.928792			PQ605942	PV617781			PV633181		PV632859					
23_Rpro	23	prolixus	*R.prolixus*	Colombia	Casanare	Villanueva	4.608736	-72.928792			PQ605941				PV633132							
24_Rpro	24	prolixus	*R.prolixus*	Colombia	Casanare	Villanueva	4.608736	-72.928792			PQ605940			PV632607					PV633668		PV632381	
26_Rpro	26	prolixus	*R.prolixus*	Colombia	Cesar	Valledupar	10.46314	-73.25322			PQ605924	PV617974		PV632606	PV633133		PV633080		PV633586		PV632138	
29_Rpro	29	prolixus	*R.prolixus*	Colombia	Cesar	Valledupar	10.46314	-73.25322						PV632605								
30_Rpro	30	prolixus	*R.prolixus*	Colombia	Cesar	Valledupar	10.46314	-73.25322				PV617782		PV632604	PV633134		PV632845					
33_Rpro	33	prolixus	*R.prolixus*	Colombia	Cesar	Valledupar	10.46314	-73.25322			PQ605939								PV633659			
47_Rpro	47	prolixus	*R.prolixus*	Colombia	Casanare	Nunchia	5.63333	-72.2			PQ605926	PV618143	PV618144	PV632528	PV633291				PV633587		PV632139	
48_Rpro	48	prolixus	*R.prolixus*	Colombia	Casanare	Nunchia	5.63333	-72.2						PV632529					PV633642		PV632140	
49_Rpro	49	prolixus	*R.prolixus*	Colombia	Casanare	Yopal -Las veredas	5.33775	-72.39586			PQ605937	PV617783		PV632533	PV633276		PV632860		PV633554		PV632110	
50_Rpro	50	prolixus	*R.prolixus*	Colombia	Casanare	Yopal -Las veredas	5.33775	-72.39586			PQ605936	PV617896		PV632532			PV632861		PV633558		PV632111	
51_Rpro	51	prolixus	*R.prolixus*	Colombia	Casanare	Yopal -Las veredas	5.33775	-72.39586				PV617894		PV632531	PV633229		PV632890		PV633340		PV632112	
52_Rpro	52	prolixus	*R.prolixus*	Colombia	Casanare	Yopal -Las veredas	5.33775	-72.39586			PQ605971	PV617892		PV632530	PV633277		PV632891		PV633341		PV632113	
53_Rpro	53	prolixus	*R.prolixus*	Colombia	Casanare	Yopal -Las veredas	5.33775	-72.39586				PV618003					PV633036		PV633613		PV632141	
54_Rpro	54	prolixus	*R.prolixus*	Colombia	Casanare	Yopal -Las veredas	5.33775	-72.39586						PV632520			PV633035				PV632114	
55_Rpro	55	prolixus	*R.prolixus*	Colombia	Casanare	Yopal -Las veredas	5.33775	-72.39586				PV618199	PV618200	PV632519			PV633090		PV633675		PV632115	
56_Rpro	56	prolixus	*R.prolixus*	Colombia	Casanare	Yopal -Las veredas	5.33775	-72.39586				PV617784		PV632498	PV633138		PV633078		PV633416		PV632116	

57_Rpro	57	prolixus	*R.prolixus*	Colombia	Casanare	Yopal -Las veredas	5.33775	-72.39586									PV632862				PV632117	
73_Rpro	73	prolixus	*R.prolixus*	Colombia	Casanare	Mani	4.818055556	-72.28111111				PV618001		PV632591			PV632973		PV633559		PV632142	
74_Rpro	74	prolixus	*R.prolixus*	Colombia	Casanare	Mani	4.818055556	-72.28111111				PV618191	PV618192	PV632592			PV632974		PV633614		PV632143	
75_Rpro	75	prolixus	*R.prolixus*	Colombia	Casanare	Mani	4.818055556	-72.28111111				PV617997		PV632593	PV633270		PV633089		PV633615		PV632144	
76_Rpro	76	prolixus	*R.prolixus*	Colombia	Casanare	Mani	4.818055556	-72.28111111						PV632594	PV633306						PV632145	
77_Rpro	77	prolixus	*R.prolixus*	Colombia	Casanare	Mani	4.818055556	-72.28111111				PV617977		PV632595	PV633271							
78_Rpro	78	prolixus	*R.prolixus*	Colombia	Casanare	Mani	4.818055556	-72.28111111				PV617972		PV632596	PV633273				PV633616		PV632146	
79_Rpro	79	prolixus	*R.prolixus*	Colombia	Casanare	Mani	4.818055556	-72.28111111				PV618185	PV618186	PV632597	PV633308		PV633087		PV633617		PV632147	
80_Rpro	80	prolixus	*R.prolixus*	Colombia	Casanare	Mani	4.818055556	-72.28111111				PV617976		PV632598	PV633303				PV633593		PV632148	
81_Rpro	81	prolixus	*R.prolixus*	Colombia	Casanare	Mani	4.818055556	-72.28111111				PV618153	PV618154	PV632599	PV633299		PV633050		PV633588		PV632149	
82_Rpro	82	prolixus	*R.prolixus*	Colombia	Casanare	Mani	4.818055556	-72.28111111						PV632600	PV633304		PV633051		PV633646			
130_Rpro	130	prolixus	*R.prolixus*	Colombia	Meta	Restrepo	4.26211	-73.5649	PQ585970			PV617952			PV633285		PV633043		PV633460			
134_Rpro	134	prolixus	*R.prolixus*	Colombia	Cesar	Valledupar	10.46314	-73.25322	PQ585904			PV617954		PV632574	PV633247		PV633044		PV633461		PV632171	
154_Rpro	154	prolixus	*R.prolixus*	Colombia	Cesar	Valledupar	10.46314	-73.25322				PV617988		PV632568	PV633267		PV633067		PV633468		PV632167	
155_Rpro	155	prolixus	*R.prolixus*	Colombia	Cesar	Valledupar	10.46314	-73.25322				PV617945		PV632746	PV633317		PV633071		PV633469		PV632444	
156_Rpro	156	prolixus	*R.prolixus*	Colombia	Cesar	Valledupar	10.46314	-73.25322				PV617989		PV632564			PV632846		PV633633			
157_Rpro	157	prolixus	*R.prolixus*	Colombia	Cesar	Valledupar	10.46314	-73.25322	PQ586071	PQ586072		PV617990		PV632565	PV633286		PV633007		PV633368		PV632383	
158_Rpro	158	prolixus	*R.prolixus*	Colombia	Cesar	Valledupar	10.46314	-73.25322				PV617955		PV632566	PV633314		PV633060		PV633470		PV632165	
160_Rpro	160	prolixus	*R.prolixus*	Colombia	Cesar	Valledupar	10.46314	-73.25322				PV617973		PV632567					PV633418		PV632168	
161_Rpro	161	prolixus	*R.prolixus*	Colombia	Cesar	Valledupar	10.46314	-73.25322			PQ605979	PV617991		PV632788	PV633251		PV632847		PV633419		PV632230	
162_Rpro	162	prolixus	*R.prolixus*	Colombia	Cesar	Valledupar	10.46314	-73.25322				PV617953		PV632524			PV633045		PV633420		PV632439	
163_Rpro	163	prolixus	*R.prolixus*	Colombia	Cesar	Valledupar	10.46314	-73.25322	PQ586045		PQ606013	PV617979		PV632569	PV633269		PV633011		PV633527		PV632169	
164_Rpro	164	prolixus	*R.prolixus*	Colombia	Cesar	Valledupar	10.46314	-73.25322						PV632570	PV633287		PV633012		PV633421		PV632433	
165_Rpro	165	prolixus	*R.prolixus*	Colombia	Cesar	Valledupar	10.46314	-73.25322				PV617980		PV632765	PV633288		PV633075		PV633422			
166_Rpro	166	prolixus	*R.prolixus*	Colombia	Cesar	Valledupar	10.46314	-73.25322				PV617957		PV632571	PV633248		PV632982		PV633590		PV632170	
167_Rpro	167	prolixus	*R.prolixus*	Colombia	Cesar	Valledupar	10.46314	-73.25322	PQ585847			PV617951		PV632572	PV633298		PV633013		PV633591		PV632392	
168_Rpro	168	prolixus	*R.prolixus*	Colombia	Cesar	Valledupar	10.46314	-73.25322				PV617983		PV632573	PV633289		PV632983		PV633423		PV632393	
195_Rpro	195	prolixus	*R.prolixus*	Colombia	Magdalena	SNSM	10.86666667	-73.72			PQ605994											
196_Rpro	196	prolixus	*R.prolixus*	Colombia	Magdalena	SNSM	10.86666667	-73.72				PV617969		PV632575	PV633290		PV633034		PV633426		PV632099	
197_Rpro	197	prolixus	*R.prolixus*	Colombia	Magdalena	SNSM	10.86666667	-73.72			PQ605934			PV632576	PV633227				PV633427		PV632100	
198_Rpro	198	prolixus	*R.prolixus*	Colombia	Magdalena	SNSM	10.86666667	-73.72				PV617981		PV632577	PV633263				PV633471		PV632405	
199_Rpro	199	prolixus	*R.prolixus*	Colombia	Magdalena	SNSM	10.86666667	-73.72				PV617994		PV632578	PV633264		PV633046		PV633437		PV632195	
200_Rpro	200	prolixus	*R.prolixus*	Colombia	Magdalena	SNSM	10.86666667	-73.72				PV617959		PV632579	PV633294		PV632984		PV633428		PV632445	
202_Rpro	202	prolixus	*R.prolixus*	Colombia	Magdalena	SNSM	10.86666667	-73.72			PQ605995	PV617985		PV632580	PV633310		PV632985		PV633579		PV632442	
203_Rpro	203	prolixus	*R.prolixus*	Colombia	Magdalena	SNSM	10.86666667	-73.72	PQ585991			PV617982		PV632581	PV633260		PV633015		PV633563		PV632197	
210_Rpro	210	prolixus	*R.prolixus*	Colombia	Magdalena	SNSM	10.86666667	-73.72						PV632582	PV633261		PV633062		PV633371		PV632198	
211_Rpro	211	prolixus	*R.prolixus*	Colombia	Magdalena	SNSM	10.86666667	-73.72			PQ605978	PV617925		PV632768	PV633265		PV633084		PV633414		PV632199	
212_Rpro	212	prolixus	*R.prolixus*	Colombia	Magdalena	SNSM	10.86666667	-73.72	PQ586047			PV618135	PV618136	PV632583					PV633458			
213_Rpro	213	prolixus	*R.prolixus*	Colombia	Magdalena	SNSM	10.86666667	-73.72			PQ606006	PV617926		PV632584					PV633478		PV632424	
214_Rpro	214	prolixus	*R.prolixus*	Colombia	Magdalena	SNSM	10.86666667	-73.72						PV632585					PV633479		PV632196	
215_Rpro	215	prolixus	*R.prolixus*	Colombia	Magdalena	SNSM	10.86666667	-73.72						PV632586							PV632436	
216_Rpro	216	prolixus	*R.prolixus*	Colombia	Magdalena	SNSM	10.86666667	-73.72			PQ605935			PV632769	PV633297		PV633069		PV633412		PV632414	
218_Rpro	218	prolixus	*R.prolixus*	Colombia	Magdalena	SNSM	10.86666667	-73.72						PV632587	PV633318		PV633085		PV633372		PV632193	
219_Rpro	219	prolixus	*R.prolixus*	Colombia	Magdalena	SNSM	10.86666667	-73.72			PQ605970			PV632588	PV633266		PV633083		PV633413			
220_Rpro	220	prolixus	*R.prolixus*	Colombia	Magdalena	SNSM	10.86666667	-73.72						PV632589	PV633204		PV632848		PV633373		PV632415	
221_Rpro	221	prolixus	*R.prolixus*	Colombia	Magdalena	SNSM	10.86666667	-73.72			PQ605821			PV632590	PV633311		PV633049				PV632421	
222_Rpro	222	prolixus	*R.prolixus*	Colombia	Magdalena	SNSM	10.86666667	-73.72						PV632629			PV632987				PV632173	
223_Rpro	223	prolixus	*R.prolixus*	Colombia	Magdalena	SNSM	10.86666667	-73.72						PV632630	PV633307		PV633047					
430_Rpro	430	prolixus	*R.prolixus*	Venezuela			6.42375	-66.5897				PV617834		PV632660	PV633259		PV633031		PV633661		PV632262	
431_Rpro	431	prolixus	*R.prolixus*	Venezuela			6.42375	-66.5897	PQ586028		PQ605868											
432_Rpro	432	prolixus	*R.prolixus*	Venezuela			6.42375	-66.5897			PQ605869	PV617835		PV632661	PV633184		PV633004		PV633431		PV632419	
434_Rpro	434	prolixus	*R.prolixus*	Venezuela			6.42375	-66.5897				PV617836		PV632662	PV633199		PV632844		PV633508		PV632263	
436_Rpro	436	prolixus	*R.prolixus*	Venezuela			6.42375	-66.5897	PQ585973		PQ605870	PV618045	PV618046		PV633212		PV633003		PV633509			
438_Rpro	438	prolixus	*R.prolixus*	Venezuela			6.42375	-66.5897	PQ586017		PQ605871			PV632663	PV633177		PV633033				PV632264	
519_Rpro	519	prolixus	*R.prolixus*	Venezuela	Carabobo		10,1741	-67.98	PQ585853						PV633170		PV632885					
520_Rpro	520	prolixus	*R.prolixus*	Venezuela	Carabobo		10,1741	-67.98				PV617923		PV632701					PV633439			
521_Rpro	521	prolixus	*R.prolixus*	Venezuela	Carabobo		10,1741	-67.98	PQ585854					PV632702	PV633237		PV632886					
522_Rpro	522	prolixus	*R.prolixus*	Venezuela	Carabobo		10,1741	-67.98			PQ605886										PV632306	
524_Rpro	524	prolixus	*R.prolixus*	Venezuela	Carabobo		10,1741	-67.98				PV617965		PV632703					PV633658			
525_Rpro	525	prolixus	*R.prolixus*	Venezuela	Carabobo		10,1741	-67.98	PQ585887					PV632782	PV633238		PV632969		PV633504		PV632307	
528_Rpro	528	prolixus	*R.prolixus*	Venezuela	Carabobo		10,1741	-67.98	PQ586016					PV632704	PV633214		PV632993		PV633440		PV632308	
563_Rpro	563	prolixus	*R.prolixus*	Venezuela	Caracas	Malariologia	10.48801	-66.87919			PQ605892				PV633252						PV632319	
564_Rpro	564	prolixus	*R.prolixus*	Venezuela	Caracas	Malariologia	10.48801	-66.87919	PQ585949					PV632678			PV632889				PV632320	
565_Rpro	565	prolixus	*R.prolixus*	Venezuela	Caracas	Malariologia	10.48801	-66.87919			PQ605894											
567_Rpro	567	prolixus	*R.prolixus*	Venezuela	Caracas	Malariologia	10.48801	-66.87919			PQ605895				PV633186		PV633019				PV632321	
568_Rpro	568	prolixus	*R.prolixus*	Venezuela	Caracas	Malariologia	10.48801	-66.87919			PQ605954	PV617846		PV632781	PV633188		PV632888		PV633472		PV632322	
570_Rpro	570	prolixus	*R.prolixus*	Venezuela	Caracas	Malariologia	10.48801	-66.87919	PQ585988			PV617847		PV632679	PV633162				PV633473		PV632323	
572_Rpro	572	prolixus	*R.prolixus*	Venezuela	Cojedes		9.35654	-68.69221			PQ605896	PV617848		PV632680	PV633253		PV632995		PV633501		PV632324	
574_Rpro	574	prolixus	*R.prolixus*	Venezuela	Cojedes		9.35654	-68.69221			PQ605897	PV618115	PV618116	PV632681	PV633254				PV633474		PV632325	
576_Rpro	576	prolixus	*R.prolixus*	Venezuela	Cojedes		9.35654	-68.69221	PQ585950		PQ605898	PV618055	PV618056	PV632682	PV633219		PV633065		PV633475		PV632326	
578_Rpro	578	prolixus	*R.prolixus*	Venezuela	Cojedes		9.35654	-68.69221	PQ585989		PQ605899	PV617849		PV632683	PV633230		PV632996				PV632327	
580_Rpro	580	prolixus	*R.prolixus*	Venezuela	Cojedes		9.35654	-68.69221	PQ585862			PV618073	PV618074	PV632684	PV633255		PV633021	PV633022	PV633432		PV632423	
582_Rpro	582	prolixus	*R.prolixus*	Venezuela	Aragua	Biomed	9.4575	-64.8261	PQ585859		PQ605900	PV618057	PV618058	PV632685	PV633256		PV633066		PV633476		PV632328	
584_Rpro	584	prolixus	*R.prolixus*	Venezuela	Aragua	Biomed	9.4575	-64.8261	PQ586040		PQ605901				PV633220		PV633001		PV633477		PV632329	
586_Rpro	586	prolixus	*R.prolixus*	Venezuela	Aragua	Biomed	9.4575	-64.8261	PQ585872		PQ605902	PV617873			PV633257		PV633023		PV633433		PV632387	
588_Rpro	588	prolixus	*R.prolixus*	Venezuela	Aragua	Biomed	9.4575	-64.8261	PQ585849			PV618059	PV618060	PV632783	PV633209		PV632999		PV633643		PV632330	
590_Rpro	590	prolixus	*R.prolixus*	Venezuela	Aragua	Biomed	9.4575	-64.8261			PQ605903	PV617850		PV632712	PV633258		PV633000		PV633434			
719_Rpro	719	prolixus	*R.prolixus*	Colombia	Arauca	Arauca	7,08471	-70.75	PQ585937		PQ605990	PV618109	PV618110	PV632716	PV633221				PV633601		PV632367	
721_Rpro	721	prolixus	*R.prolixus*	Colombia	Arauca	Arauca	7,08471	-70.75	PQ586020		PQ605964	PV617903		PV632696					PV633572		PV632368	
722_Rpro	722	prolixus	*R.prolixus*	Colombia	Arauca	Tame	6.45	-71.7667	PQ585890		PQ605917	PV618065	PV618066	PV632697	PV633262				PV633401		PV632369	
723_Rpro	723	prolixus	*R.prolixus*	Colombia	Arauca	Tame	6.45	-71.7667	PQ585913		PQ606001	PV618111	PV618112	PV632698	PV633222		PV632964		PV633576		PV632426	
774_Rpro	774	prolixus	*R.prolixus*	Colombia	Arauca	Arauca	7,08471	-70.75	PQ586008		PQ605992	PV618091	PV618092	PV632521	PV633149		PV633024		PV633599		PV632378	
775_Rpro	775	prolixus	*R.prolixus*	Colombia	Arauca	Arauca	7,08471	-70.75	PQ586077	PQ586078	PQ605966	PV618093	PV618094	PV632522	PV633152		PV633025		PV633608		PV632379	
68_Rpro	68	prolixus	*R.prolixus*	Colombia	Antioquia	Necocli	8.4357	-76.7767				PV617792		PV632497	PV633243		PV632863		PV633353		PV632130	

69_Rpro	69	prolixus	*R.prolixus*	Colombia	Antioquia	Necocli	8.4357	-76.7767			PQ605928	PV617793		PV632495	PV633272		PV632856		PV633671		PV632131	
70_Rpro	70	prolixus	*R.prolixus*	Colombia	Antioquia	Necocli	8.4357	-76.7767			PQ605929	PV617891		PV632494	PV633244		PV632852		PV633348		PV632132	
71_Rpro	71	prolixus	*R.prolixus*	Colombia	Antioquia	Necocli	8.4357	-76.7767			PQ605930	PV617794		PV632493	PV633223		PV632850		PV633349		PV632133	
72_Rpro	72	prolixus	*R.prolixus*	Colombia	Antioquia	Necocli	8.4357	-76.7767			PQ605927	PV617868		PV632492	PV633245		PV632875		PV633354		PV632134	
343_Rpro	343	prolixus	*R.prolixus*	Brasil	Rio de Janeiro	Rio de Janeiro	-22.9064	-43.18223	PQ585927													
345_Rpro	345	prolixus	*R.prolixus*	Brasil	Rio de Janeiro	Rio de Janeiro	-22.9064	-43.18223	PQ585928													
346_Rpro	346	prolixus	*R.prolixus*	Brasil	Rio de Janeiro	Rio de Janeiro	-22.9064	-43.18223							PV633208				PV633521			
347_Rpro	347	prolixus	*R.prolixus*	Brasil	Rio de Janeiro	Rio de Janeiro	-22.9064	-43.18223	PQ585889													
349_Rpro	349	prolixus	*R.prolixus*	Brasil	Rio de Janeiro	Rio de Janeiro	-22.9064	-43.18223						PV632752								
351_Rpro	351	prolixus	*R.prolixus*	Brasil	Rio de Janeiro	Rio de Janeiro	-22.9064	-43.18223	PQ585981													
126_Rrob	126	prolixus	*R.robustus*	Peru	Lima	ND	-29.68	-51.46				PV617975		PV632560			PV633042				PV632250	
99_Rrob	99	prolixus	*R.robustus*	Peru	Lima	ND	-12.06	-77.0375			PQ605974	PV618201	PV618202	PV632539	PV633305		PV633037		PV633595		PV632158	
100_Rrob	100	prolixus	*R.robustus*	Peru	Lima	ND	-12.06	-77.0375			PQ605997	PV618155	PV618156	PV632540	PV633309		PV632976		PV633560		PV632159	
101_Rrob	101	prolixus	*R.robustus*	Peru	Lima	ND	-12.06	-77.0375	PQ585877		PQ606012	PV618075	PV618076	PV632541	PV633300		PV633010		PV633602		PV632402	
102_Rrob	102	prolixus	*R.robustus*	Peru	Lima	ND	-12.06	-77.0375	PQ585903		PQ606015	PV618157	PV618158		PV633296		PV632977		PV633635		PV632400	
103_Rrob	103	prolixus	*R.robustus*	Peru	Lima	ND	-12.06	-77.0375	PQ586004		PQ606017	PV618077	PV618078	PV632542	PV633331	PV633332	PV633038		PV633623		PV632395	
104_Rrob	104	prolixus	*R.robustus*	Peru	Lima	ND	-12.06	-77.0375				PV618197	PV618198	PV632543			PV633039		PV633603		PV632434	
106_Rrob	106	prolixus	*R.robustus*	Peru	Lima	ND	-12.06	-77.0375			PQ605975	PV618139	PV618140	PV632545	PV633275				PV633561		PV632431	
107_Rrob	107	prolixus	*R.robustus*	Peru	Lima	ND	-12.06	-77.0375	PQ585986		PQ605976	PV617999		PV632546	PV633327	PV633328			PV633573		PV632166	
108_Rrob	108	prolixus	*R.robustus*	Peru	Lima	ND	-12.06	-77.0375	PQ586002			PV618123	PV618124	PV632547	PV633329	PV633330	PV633091		PV633625		PV632427	
280_Rrob	280	prolixus	*R.robustus*	Brasil	Ceara	Varzea Alegre	-5.35	-40.3833	PQ585891		PQ605836	PV617806		PV632800					PV633406		PV632205	
282_Rrob	282	prolixus	*R.robustus*	Brasil	Ceara	Varzea Alegre	-5.35	-40.3833	PQ585941		PQ605837	PV617807					PV632865		PV633491		PV632206	
284_Rrob	284	prolixus	*R.robustus*	Brasil	Ceara	Varzea Alegre	-5.35	-40.3833	PQ585994			PV617808		PV632803							PV632207	
287_Rrob	287	prolixus	*R.robustus*	Brasil	Ceara	Varzea Alegre	-5.35	-40.3833	PQ585958		PQ605838	PV617809		PV632499			PV633028		PV633664		PV632208	
289_Rrob	289	prolixus	*R.robustus*	Brasil	Ceara	Varzea Alegre	-5.35	-40.3833	PQ586037			PV617810							PV633511		PV632217	
290_Rrob	290	prolixus	*R.robustus*	Brasil	Ceara	Varzea Alegre	-5.35	-40.3833						PV632500								
291_Rrob	291	prolixus	*R.robustus*	Brasil	Ceara	Varzea Alegre	-5.35	-40.3833	PQ585858													
292_Rrob	292	prolixus	*R.robustus*	Brasil	Ceara	Varzea Alegre	-5.35	-40.3833	PQ585935		PQ605948	PV617812		PV632501					PV633656		PV632218	
307_Rrob	307	prolixus	*R.robustus*	Brasil	Oriximin	Caipuru	-1.672	-55.884				PV617813		PV632502	PV633213		PV633099	PV633100	PV633350		PV632459	PV632460
308_Rrob	308	prolixus	*R.robustus*	Brasil	Oriximin	Caipuru	-1.672	-55.884			PQ605845	PV617946		PV632715	PV633203		PV632869		PV633514		PV632461	PV632462
311_Rrob	311	prolixus	*R.robustus*	Brasil	Oriximin	Caipuru	-1.672	-55.884						PV632503								
312_Rrob	312	prolixus	*R.robustus*	Brasil	Oriximin	Caipuru	-1.672	-55.884	PQ585873						PV633218							
314_Rrob	314	prolixus	*R.robustus*	Brasil	Oriximin	Caipuru	-1.672	-55.884	PQ585870						PV633249							
316_Rrob	316	prolixus	*R.robustus*	Brasil	Oriximin	Caipuru	-1.672	-55.884	PQ586049	PQ586050					PV633250							
317_Rrob	317	prolixus	*R.robustus*	Brasil	Oriximin	Caipuru	-1.672	-55.884													PV632213	
318_Rrob	318	prolixus	*R.robustus*	Brasil	Oriximin	Caipuru	-1.672	-55.884	PQ585951													
440_Rrob	440	prolixus	*R.robustus*	Peru	Lima		-12.06	-77.0375	PQ585997		PQ605872	PV618047	PV618048	PV632664	PV633200		PV632893		PV633381		PV632265	
445_Rrob	445	prolixus	*R.robustus*	Peru	Lima		-12.06	-77.0375				PV617961					PV633009		PV633663		PV632266	
446_Rrob	446	prolixus	*R.robustus*	Peru	Lima		-12.06	-77.0375	PQ585856			PV618049	PV618050	PV632665	PV633193		PV633105	PV633106	PV633382		PV632267	
448_Rrob	448	prolixus	*R.robustus*	Peru	Lima		-12.06	-77.0375	PQ585857			PV617872		PV632771	PV633211		PV633008				PV632268	
530_Rrob	530	prolixus	*R.robustus*	Venezuela	Trujillo	Trujillo	9.36659	-70.4349							PV633145		PV633017					
531_Rrob	531	prolixus	*R.robustus*	Venezuela	Trujillo	Trujillo	9.36659	-70.4349							PV633153						PV632309	
532_Rrob	532	prolixus	*R.robustus*	Venezuela	Trujillo	Trujillo	9.36659	-70.4349						PV632705	PV633154		PV632992				PV632310	
533_Rrob	533	prolixus	*R.robustus*	Venezuela	Trujillo	Trujillo	9.36659	-70.4349			PQ605887			PV632801			PV632968		PV633441			
535_Rrob	535	prolixus	*R.robustus*	Venezuela	Merida		8.5952400	-71.1434000				PV617933		PV632706	PV633231		PV632887		PV633384			
536_Rrob	536	prolixus	*R.robustus*	Venezuela	Merida		8.5952400	-71.1434000	PQ585931								PV633063					
537_Rrob	537	prolixus	*R.robustus*	Venezuela	Merida		8.5952400	-71.1434000			PQ605888	PV617960		PV632774					PV633655		PV632311	
538_Rrob	538	prolixus	*R.robustus*	Venezuela	Merida		8.5952400	-71.1434000							PV633198		PV633016				PV632312	
539_Rrob	539	prolixus	*R.robustus*	Venezuela	Merida		8.5952400	-71.1434000			PQ605889	PV617966		PV632707								
540_Rrob	540	prolixus	*R.robustus*	Venezuela	Merida		8.5952400	-71.1434000							PV633295		PV633018				PV632313	
541_Rrob	541	prolixus	*R.robustus*	Venezuela	Merida		8.5952400	-71.1434000			PQ605890	PV617927		PV632747					PV633673			
543_Rrob	543	prolixus	*R.robustus*	Venezuela	Merida		0.45920001	-77.52	PQ586025		PQ605891	PV617934		PV632775	PV633232				PV633442		PV632412	
555_Rrob	555	prolixus	*R.robustus*	Ecuador	Sucumbios	Pacokocha	0.45920001	-77.52											PV633731	PV633732		
556_Rrob	556	prolixus	*R.robustus*	Ecuador	Sucumbios	Pacokocha	-1.0522814	-77.6889367			PQ605893				PV633174		PV632994		PV633737	PV633738		
561_Rrob	561	prolixus	*R.robustus*	Ecuador	Napo	Jatun Sacha	8.5952400	-71.1434000									PV633064		PV633735	PV633736	PV632318	
332_Rsta	332	pictipes	*R.stali*	Bolivia	Caranavi	Alto Beni	-15.805	-67.2	PQ586033					PV632710					PV633456		PV632233	
333_Rsta	333	pictipes	*R.stali*	Bolivia	Caranavi	Alto Beni	-15.805	-67.2				PV617917			PV633207							
334_Rsta	334	pictipes	*R.stali*	Bolivia	Caranavi	Alto Beni	-15.805	-67.2											PV633632		PV632234	
335_Rsta	335	pictipes	*R.stali*	Bolivia	Caranavi	Alto Beni	-15.805	-67.2	PQ585966			PV618101	PV618102	PV632509					PV633707	PV633708	PV632235	
337_Rsta	337	pictipes	*R.stali*	Bolivia	Caranavi	Alto Beni	-15.805	-67.2	PQ585967			PV617889		PV632711					PV633699	PV633700	PV632236	
338_Rsta	338	pictipes	*R.stali*	Bolivia	Caranavi	Alto Beni	-15.805	-67.2							PV633226							
339_Rsta	339	pictipes	*R.stali*	Bolivia	Caranavi	Alto Beni	-15.805	-67.2	PQ585855			PV617814		PV632631					PV633551		PV632237	
340_Rsta	340	pictipes	*R.stali*	Bolivia	Caranavi	Alto Beni	-15.805	-67.2							PV633180							
341_Rsta	341	pictipes	*R.stali*	Bolivia	Caranavi	Alto Beni	-15.805	-67.2	PQ585871			PV617815		PV632632			PV632904		PV633519		PV632238	
490_Rsta	490	pictipes	*R.stali*	Brasil	Mato Grosso do Sul	Corumba	-29.68	-51.46				PV618195	PV618196	PV632667					PV633402			
492_Rsta	492	pictipes	*R.stali*	Brasil	Mato Grosso do Sul	Corumba	-29.68	-51.46			PQ605880			PV632668	PV633192		PV632903		PV633447		PV632292	
494_Rsta	494	pictipes	*R.stali*	Brasil	Mato Grosso do Sul	Corumba	-29.68	-51.46			PQ605874	PV617874		PV632669					PV633650		PV632293	
495_Rsta	495	pictipes	*R.stali*	Brasil	Mato Grosso do Sul	Corumba	-19.0098	-57.6547				PV617841									PV632294	
496_Rsta	496	pictipes	*R.stali*	Brasil	Mato Grosso do Sul	Corumba	-19.0098	-57.6547						PV632670								
497_Rsta	497	pictipes	*R.stali*	Brasil	Mato Grosso do Sul	Corumba	-19.0098	-57.6547				PV617916		PV632671					PV633448		PV632295	
603_Rsta	603	pictipes	*R.stali*	Bolivia			-16.290154	-63.5886						PV632784							PV632332	
604_Rsta	604	pictipes	*R.stali*	Bolivia			-16.290154	-63.5886			PQ605904	PV618095	PV618096								PV632418	
605_Rsta	605	pictipes	*R.stali*	Bolivia			-16.290154	-63.5886			PQ605905			PV632713							PV632333	
606_Rsta	606	pictipes	*R.stali*	Bolivia			-16.290154	-63.5886			PQ605906										PV632334	
607_Rsta	607	pictipes	*R.stali*	Bolivia			-16.290154	-63.5886											PV633733	PV633734	PV632335	
608_Rsta	608	pictipes	*R.stali*	Bolivia			-16.290154	-63.5886			PQ605907								PV633729	PV633730		
609_Rsta	609	pictipes	*R.stali*	Bolivia			-16.290154	-63.5886			PQ605908	PV618061	PV618062						PV633703	PV633704	PV632336	
610_Rsta	610	pictipes	*R.stali*	Bolivia			-16.290154	-63.5886											PV633538			
Out_Tri	Out	Triatomini	*T. dimidiata*	Colombia	Boyacá		7.033	-72.067														
Out_Tri2	Out	Triatomini	*T. dimidiata*	Colombia	Boyacá		7.033	-72.067														
Out_Pans1	Out	Triatomini	*P. geniculatus*	Colombia	Meta		4,26211	-73,5649														
Out_Pans2	Out	Triatomini	*P. geniculatus*	Colombia	Meta		4,26211	-73,5649														
Out_Pans4	Out	Triatomini	*P. geniculatus*	Colombia	Meta		4,26211	-73,5649														
Out_Pans5	Out	Triatomini	*P. geniculatus*	Colombia	Meta		4,26211	-73,5649														

Supplementary Table 2. Population genetics summary statistics for each species per locus

Species	n	Segregating sites (SS)	Nucleotide diversity (π)	Haplotypes
Part	13	10	0.00544	6
Pcor	21	18	0.0075	5
Pter	6	17	0.01626	6
Recu	15	11	0.00772	4
Rpal	48	38	0.03469	7
Rcol	7	3	0.00288	2
Rsta	12	14	0.01482	2
Rpic	2	1	0.002	2
Rbre	11	0	0	0
				pr
Rneg	24	103	0.04892	6
Rnas	10	40	0.01597	2
Rmil	6	0	0	0
				pro
Rmar	3	0	0	0
Rmon	18	41	0.01907	5
Rpro	101	36	0.00444	13
Rrob	30	47	0.03547	6
**prolixus_1**	40	115	0.03917	9
**prolixus_2**	152	57	0.03032	18
Psammo	40	120	0.08939	16
pallescens	70	92	0.06618	13
pictipes	40	70	0.03741	5
prolixus	192	154	0.08603	36

Species	n	Segregating sites (SS)	Nucleotide diversity (π)	Haplotipos
Part	32	2	0.00039	3
Pcor	6	0	0	0
Pter	6	5	0.00436	5
Recu	6	0	0	0
Rpal	24	0	0	0
Rcol	5	0	0	0
Rsta	5	8	0.00679	2
Rnei	7	3	0.00321	3
				pr
Rneg	14	12	0.0045	4
Rnas	4	1	0.00108	2
Rmil	10	2	0.00241	2
				pro
Rmar	4	1	0.00105	2
Rmon	15	1	0.001	2

Rpro	57	5	0.00046	5
Rrob	23	0	0	0
**prolixus_1**	28	16	0.00509	8
**prolixus_2**	99	6	0.0008	6
Psammo	44	9	0.00399	8
pallescens	35	9	0.00194	3
pictipes	5	8	0.00536	3
prolixus	127	21	0.00428	11

**Species**	n	**Segregating sites (SS)**	**Nucleotide diversity (π)**	**Haplotipos**
Part	17	1	0.00023	2
Pcor	15	9	0.00404	8
Pter	19	6	0.00376	10
				[illegible]
Recu	11	2	0.0007	2
Rpal	25	11	0.00356	9
Rcol	6	3	0.00192	3
Rsta	7	16	0.00914	5
Rpic	3	2	0.00256	3
Rbre	3	0	0	0
Rnei	2	0	0	0
				[illegible]
Rneg	14	8	0.00373	7
Rnas	9	0	0	0
Rmil	7	0	0	0
				[illegible]
Rmar	1	0	0	0
Rmon	18	5	0.00235	5
Rpro	61	9	0.00188	11
Rrob	21	3	0.00126	3
**prolixus_1**	30	8	0.00316	8
**prolixus_2**	105	17	0.00341	19
Psammo	51	18	0.00787	18
pallescens	42	25	0.01012	13
pictipes	13	22	0.01302	9
prolixus	135	29	0.0063	25

**Species**	N	**Segregating sites (SS)**	**Nucleotide diversity (π)**	**Haplotipos**
Part	30	0	0	0
Pcor	28	3	0.00146	4
Pter	18	0	0	0

				[illegible]
Recu	13	0	0	0
Rpal	53	7	0.00207	9
Rcol	11	0	0	0
Rsta	16	5	0.00188	5
Rpic	7	5	0.00398	6
Rbre	9	0	0	0
Rnei	6	0	0	0
				pr
Rneg	24	2	0.00077	3
Rnas	9	4	0.00155	3
Rmil	12	0	0	0
				pro
Rmar	3	0	0	0
Rmon	15	3	0.00089	4
Rpro	96	1	0.00007	2
Rrob	40	6	0.00156	6
**prolixus_1**	45	6	0.00124	5
**prolixus_2**	151	7	0.00121	7
Psammo	76	24	0.0186	6
pallescens	77	7	0.00207	9
pictipes	32	5	0.00398	6
prolixus		1	0.00007	2

**Species**		**Segregating sites (SS)**	**Nucleotide diversity (π)**	**Haplotipos**
Part	35	2	0.0003	3
Pcor	20	11	0.00271	5
Pter	25	3	0.00167	5
				[illegible]
Recu	16	4	0.00253	5
Rpal	42	22	0.00242	4
Rcol	10	0	0	0
Rsta	19	8	0.00459	7
Rpic	10	11	0.00793	9
Rbre	10	0	0	0
Rnei	8	12	0,00542	3
				pr
Rneg	25	30	0.00548	7
Rnas	11	17	0.00957	3
Rmil	13	1	0.00027	2
				pro
Rmar	4	0	0	0
Rmon	24	19	0.00766	17
Rpro	94	23	0.00132	6
Rrob	35	11	0.0029	10

**prolixus_1**	49	42	0.00826	12
**prolixus_2**	157	43	0.00381	29
Psammo	80	16	0.00804	12
pallescens	68	40	0.0142	10
pictipes	39	11	0.00793	9
prolixus	206	54	0.00817	35

**Species**		**Segregating sites (SS)**	**Nucleotide diversity (π)**	**Haplotipos**
Part	38	4	0.00141	6
Pcor	31	5	0.00253	8
Pter	21	3	0.00156	5
				[illegible]
Recu	16	5	0.00205	5
Rpal	75	12	0.00319	17
Rcol	11	1	0.00073	2
Rsta	15	8	0.00571	8
Rpic	8	12	0.00885	8
Rbre	11	3	0.00201	3
Rnei	4	2	0.00168	3
				pr
Rneg	11	4	0.00256	5
Rnas	7	25	0.01196	2
Rmil	11	6	0.00305	4
				pro
Rmar	2	0	0	0
Rmon	27	4	0.00172	6
Rpro	95	10	0.00394	24
Rrob	45	10	0.00364	14
**prolixus_1**	29	33	0.01049	11
**prolixus_2**	169	18	0.00399	39
Psammo	90	24	0.01444	18
pallescens	102	42	0.0157	24
pictipes	34	33	0.0188	19
prolixus	198	60	0.01382	52

**Species**		**Segregating sites (SS)**	**Nucleotide diversity (π)**	**Haplotipos**
Part	33	3	0.00094	4
Pcor	28	4	0.00302	6
Pter	14	1	0.00027	2
				[illegible]
Recu	15	31	0.09193	4
Rpal	24	3	0.00104	4
Rcol	8	0	0	0

Rsta	2	4	0.00753	2
Rnei	6	0	0	0
				pr
Rneg	19	9	0.00625	6
Rnas	8	4	0.00344	4
Rmil	8	1	0.00041	2
				pr
Rmar	6	6	0.00506	5
Rmon	19	4	0.00288	7
Rpro	88	7	0.00067	9
Rrob	35	7	0.00136	7
**prolixus_1**	35	14	0.00754	11
**prolixus_2**	148	16	0.00211	21
Psammo	75	18	0.01883	8
pallescens	47	305	0.04073	9
pictipes	2	4	0.00753	2
prolixus	189	51	0.0709	32

Species		Segregating sites (SS)	Nucleotide diversity (π)	Haplotipos
Part	10	2	0.0008	3
Pcor	2	1	0.00199	2
				[illegible]
Recu	5	1	0.0012	2
Rpal	9	5	0.00411	3
Rcol	10	17	0.01587	2
Rsta	4	3	0.003	2
				pr
Rneg	8	7	0.00581	3
Rmil	4	1	0.00099	2
Rnas	10	8	0.00583	4
				pr
Rmar	3	1	0.00133	2
Rmon	17	2	0.00067	3
Rpro	96	17	0.00071	2
Rrob	29	3	0.00108	4
**prolixus_1**	22	9	0.00669	5
**prolixus_2**	167	19	0.00126	7
Psammo	12	11	0.0083	6
pallescens	24	23	0.0148	7
pictipes	4	3	0.003	2
prolixus	189	26	0.00331	12

CYTB

Haplotype diversity (Hd)	Tajima's D		Fu and Li's D		Fu and Li's D	
Psammolestes						
0.641	-0.62546	NS	-0.208	NS	-0.3624	NS
0.49	-0.94379	NS	0.671	NS	0.22442	NS
1	0.51771	NS	0.36447	NS	0.43204	NS
pallescens group						
0.705	0.55106	NS	-0.14923	NS	0.04744	NS
0.797	3.51233	***, P < 0.001	1.24425	NS	2.44508	**, P < 0.02
0.476	0.75467	NS	1.2975	NS	1.26871	NS
pictipes group						
0.53	2.56604	**, P < 0.01	1.49698	**, P < 0.02	2.01801	**, P < 0.02
1	0	0	0	0	0	0
0	0	0	0	0	0	0
olixus group clade I						
0.768	-0.72743	P > 0.10	-1.45377	NS	-1.43795	NS
0.2	-2.10795	***, P < 0.001	-2.4987	**, P < 0.02	-2.71104	**, P < 0.02
0	0	0	0	0	0	0
olixus group clade II						
0	0	0	0	0	0	0
0.797	-0.81689	NS	0.74779	NS	0.33883	NS
0.815	-2.1335	*, P < 0.05	-6.18812	**, P < 0.02	-5.4863	**, P < 0.02
0.754	1.83857	NS	1.66912	**, P < 0.02	2.03592	**, P < 0.02
0.853	-1.28178	NS	-2.33282	NS	-2.33148	NS
0.892	1.25225	NS	1.6819	*P < 0.05	1.79974	*, P < 0.05
0.817	1.77495	0.10 > P > 0.05	1.44641	P < 0.05	1.86292	*, P < 0.05
0.888	2.0962	*, P < 0.05	1.70869	**, P < 0.02	2.23348	**, P < 0.02
0.713	-0.06647	NS	1.72773	**, P < 0.02	1.36059	P > 0.10
0.00002	1.0418	NS	1.35513	NS	1.42901	NS

28S

Haplotype diversity (Hd)	Tajima's D		Fu and Li's D		Fu and Li's T	
Psammolestes						
0.179	-1.26710	NS	-0.75852	NS	-1.04361	NS
0	0	0	0	0	0	0
0.933	-0.31466	NS	-0.21471	NS	-0.25135	NS
pallescens group						
0	0	0	0	0	0	0
0	0	0	0	0	0	0
0	0	0	0	0	0	0
pictipes group						
0.4	-1.17432	NS	-1.17432	NS	-1.22979	NS
prolixus group						
0.667	1.10686	NS	1.2975	NS	1.3533	NS
olixus group clade I						
0.626	-1.80212	P < 0.05	-2.49598	P < 0.05	-2.64383	*, P < 0.05
0.5	-0.61237	NS	-0.61237	NS	-0.47871	NS
0.556	1.84427	NS	1.02623	NS	1.3643	NS
olixus group clade II						
0.5	-0.61237	NS	-0.61237	NS	-0.47871	NS
0.476	1.12241	NS	0.70104	NS	0.91825	NS

0.137	**-1.90386**	**P < 0.05**	**2.84804**	**P < 0.05**	**-2.98878**	***, P < 0.05**
0	0	0	0	0	0	0
0.76	-1.20351	P > 0.10	-2.55137	P < 0.05	-2.48503	NS
0.298	-1.55741	NS	**-2.67045**	**P < 0.05**	**-2.71616**	**P < 0.05**
0.54	-0.25384	NS	-1.20649	NS	-1.0586	NS
0.294	-1.77423	NS	**-2.91016**	**P < 0.05**	**-2.99408**	***, P < 0.05**
0.524	-1.66336	NS	**-1.64083**	**P < 0.05**	**-1.84116**	***, P < 0.05**
0.555	-1.52354	NS	**-3.01216**	**P < 0.05**	**-2.91948**	***, P < 0.05**

**CISP**						
**Haplotype diversity (Hd)**	**Tajima's D**		**Fu and Li's D**		**Fu and Li's T**	
Psammolestes						
0.118	-1.16387	NS	-1.47711	NS	-1.59107	NS
0.848	-0.91126	NS	-0.48551	NS	-0.68888	NS
0.737	0.44602	NS	1.25359	NS	1.18582	NS
pallescens group						
0.182	1.42961	NS	-1.65766	NS	-1.79737	NS
0.797	-1.23557	NS	-1.87923	NS	-1.96599	NS
0.6	-1.23311	NS	-1.26013	NS	-1.31835	NS
pictipes group						
0.857	1.50288	NS	-1.55437	NS	-1.69821	NS
1	0	NS	0	NS	0	NS
0	0	0	0	0	0	0
prolixus group						
0	0	0	0	0	0	0
olixus group clade I						
0.802	-0.85527	NS	-1.15591	NS	-1.22965	NS
0	0	0	0	0	0	0
0	0	0	0	0	0	0
olixus group clade II						
0	0	0	0	0	0	0
0.693	-0.50397	NS	-1.13794	NS	-1.10868	NS
0.702	-1.51863	NS	**-3.09515**	**P < 0.05**	**-3.03186**	**P < 0.05**
0.495	-0.55669	NS	-1.28294	NS	-1.24655	NS
0.807	-0.5617	NS	-1.21103	NS	-1.18376	NS
0.772	-1.56629	NS	-2.31274	NS	-2.4283	*, P < 0.05
0.00149	0.05971	NS	-0.37101	NS	-0.264	NS
0.847	-0.33337	NS	-1.77022	NS	-1.51627	NS
0.923	-0.1945	NS	-0.26898	NS	-0.28473	NS
0.845	-1.33675	NS	-1.7383	NS	-1.89188	NS

**LSM**						
**Haplotype diversity (Hd)**	**Tajima's D**		**Fu and Li's D**		**Fu and Li's T**	
Psammolestes						
0	0	0	0	0	0	0
0.664	0.19434	NS	-0.24045	NS	-0.13462	NS
0	0	0	0	0	0	0

pallescens group						
0	0	0	0	0	0	0
0.766	-0.57546	NS	-1.12427	NS	-1.1139	NS
0	0	0	0	0	0	0
pictipes group						
0.0202	-1.19959	NS	-1.80783	NS	-1.88431	NS
0.952	-0.09908	NS	-0.37037	NS	-0.33669	NS
0	0	0	0	0	0	0
prolixus group						
0	0	0	0	0	0	0
olixus group clade I						
0.42	-0.35434	NS	0.83728	NS	0.58558	NS
0.417	-1.60974	NS	-1.79883	NS	-1.94803	NS
0	0	0	0	0	0	0
olixus group clade II						
0	0	0	0	0	0	0
0.371	-1.31654	NS	-1.08108	NS	-1.30356	NS
0.041	-0.9113	NS	0.4949	NS	0.08547	NS
0.494	-0.95136	NS	-0.45579	NS	-0.71189	NS
0.535	-1.35209	NS	-2.06569	NS	-2.15788	NS
0.348	-0.98318	NS	1.16213	NS	0.50136	NS
0.749	**3.58826**	*****, P < 0.001**	1.44235	NS	**2.66986**	****, P < 0.02**
0.766	-0.57546	NS	-1.12427	NS	-1.1139	NS
0.952	-0.09908	NS	-0.09908	NS	-0.33669	NS
0.041	-0.9113	NS	0.4949	NS	0.08547	NS

PJH

Haplotype diversity (Hd)	Tajima's D		Fu and Li's D		Fu and Li's T	
Psammolestes						
0.113	-1.28069	NS	-0.78702	NS	-1.0741	NS
0.568	-1.79471	0.10 > P > 0.05	**-2.52589**	***, P < 0.05**	**-2.68344**	***, P < 0.05**
0.717	0.47565	NS	0.97378	NS	0.96257	NS
pallescens group						
0.7	0.56976	NS	0.25371	NS	0.38717	NS
0.418	**-2.42609**	****, P < 0.01**	**-5.11737**	****, P < 0.02**	**-4.97843**	****, P < 0.02**
0	0	0	0	0	0	0
pictipes group						
0.00184	0.45962	NS	0.23166	NS	0.34198	NS
0.978	0.69159	NS	0.44989	NS	0.57344	NS
0	0	0	0	0	0	0
prolixus group						
0.464	**-1.76999**	***, P < 0.05**	**-1.94038**	****, P < 0.02**	**-2.10856**	***, P < 0.05**
olixus group clade I						
0.58	**-2.28061**	****, P < 0.01**	**-3.8356**	****, P < 0.02**	**-3.9286**	****, P < 0.02**
0.345	-0.29777	NS	1.29104	0.10 > P > 0.05	1.00393	P > 0.10
0.154	-1.14915	NS	-1.36547	NS	-1.48111	NS
olixus group clade II						
0	0	0	0	0	0	0
0.938	-0.53806	NS	-1.10537	P > 0.10	-1.08977	NS
0.315	**-2.52034**	*****, P < 0.001**	**-6.64822**	****, P < 0.02**	**-6.07627**	****, P < 0.02**
0.568	-1.1984		-0.13116	NS	-0.55	NS

0.809	-1.79304	NS	**-2.52191**	***, P < 0.05**	**-2.69232**	***, P < 0.05**
0.584	**-2.22708**	****, P < 0.01**	**-6.00181**	****, P < 0.02**	**-5.29308**	****, P < 0.02**
0.77	1.17856	NS	0.62826	NS	0.98298	NS
0.745	-0.21749	NS	**-2.37706**	***, P < 0.05**	-1.86228	NS
0.978	0.69159	NS	0.44989	NS	0.57344	NS
0.702	-1.61759	0.10 > P > 0.05	**-4.15952**	****, P < 0.02**	**-3.64874**	****, P < 0.02**

TRNA

Haplotype diversity (Hd)	Tajima's D		Fu and Li's D		Fu and Li's T	
Psammolestes						
0.617	-0.28629	NS	-0.02349	NS	-0.1189	NS
0.763	0.01064	NS	1.21171	NS	0.9924	NS
0.676	0.31337	NS	-0.14199	NS	-0.01903	NS
pallescens group						
0.767	-0.61637	NS	-1.05227	NS	-1.07152	NS
0.845	-0.62537	NS	-0.30337	NS	-0.49254	NS
0.436	0.67135	NS	0.77552	NS	0.83951	NS
pictipes group						
0.867	1.42067	NS	**1.36102**	*** P < 0.05**	1.57827	0.10 > P > 0.05
1	0.71493	NS	0.95107	NS	0.99404	NS
0.564	0.58729	NS	0.12672	NS	0.27078	NS
prolixus group						
0.833	-0.7099	NS	-0.7099	NS	-0.60427	NS
olixus group clade I						
0.764	0.43329	NS	0.40609	NS	0.46491	NS
0.286	**-1.70055**	***, P < 0.05**	**-1.80508**	***** P < 0.02**	**-1.96653**	***** P < 0.02**
0.691	-0.4451	NS	0.73779	NS	0.49748	NS
olixus group clade II						
0	0	0	0	0	0	0
0.752	-0.03089	NS	-0.9159	NS	-0.76575	NS
0.757	0.51892	NS	0.6776	NS	0.73857	NS
0.803	-0.40569	NS	0.88057	NS	0.54226	NS
0.01049	-0.97286	NS	-1.8177	NS	-1.82037	NS
0.827	-0.7832	NS	0.18307	NS	-0.23045	NS

0.871	**2.26052**	*** P < 0.05**	1.10356	NS	**1.84356**	*** P < 0.05**
0.905	0.40995	NS	0.84948	NS	0.80651	NS
0.932	1.39134	NS	1.13658	NS	1.4439	NS
0.876	-0.67098	NS	1.52589	0.10 > P > 0.05	0.64985	NS

UPCA

Haplotype diversity (Hd)	Tajima's D		Fu and Li's D		Fu and Li's T	
Psammolestes						
0.438	-0.84734	NS	-1.52312	NS	-1.53835	NS
0.701	1.46433	NS	0.06596	NS	0.5458	NS
0.143	-1.15524	NS	-1.39749	NS	-1.51388	NS
pallescens group						
0.676	**-2.48813**	******, P < 0.001**	**-3.49954**	****, P < 0.02**	**-3.70905**	****, P < 0.02**
0.373	-1.04056	NS	-0.18894	NS	-0.49351	NS
0	0	0	0	0	0	0
pictipes group						

1	0	0	0	0	0	0
0	0	0	0	0	0	0
olixus group clade I						
0.801	1.61368	NS	**1.38055**	***, P < 0.05**	**1.67242**	***, P < 0.05**
0.821	1.4952	NS	1.31251	NS	1.48924	NS
0.25	-1.05482	NS	-1.12639	NS	-1.20353	NS
olixus group clade II						
0.933	0.95607	NS	1.05892	NS	1.10903	NS
0.865	1.53917	NS	1.11537	NS	1.41723	NS
0.253	**-1.91955**	***, P < 0.05**	-1.8959	NS	-2.25226	NS
0.587	-1.46966	NS	**-3.07031**	***, P < 0.05**	**-3.01433**	***, P < 0.05**
0.886	1.0899	NS	1.09174	NS	1.28339	NS
0.532	-1.71299	NS	-1.56221	NS	-1.93925	NS
0.745	**3.38431**	*****, P < 0.001**	1.22369	NS	**2.38483**	****, P < 0.02**
0.775	**-2.72869**	*****, P < 0.001**	**-6.77914**	****, P < 0.02**	**-6.29825**	****, P < 0.02**
1	0	0	0	0	0	0
0.709	-1.34142	NS	0.77709	NS	-0.18188	NS
**UPMETAL**						
**Haplotype diversity (Hd)**	**Tajima's D**		**Fu and Li's D**		**Fu and Li's T**	
Psammolestes						
0.378	-1.40085	NS	-1.58662	NS	-1.71902	NS
1	0	0	0	0	0	0
pallescens group						
0.6	1.22474	NS	1.22474	NS	1.15728	NS
0.556	0.49704	NS	1.32868	NS	1.25725	NS
0.467	1.48883	NS	**1.53723**	****, P < 0.02**	**1.71865**	****, P < 0.02**
pictipes group						
0.5	-0.75445	NS	-0.75445	NS	-0.67466	NS
prolixus group						
olixus group clade I						
0.0067	1.21019	NS	1.33143	NS	1.48407	NS
0.5	-0.61237	NS	-0.61237	NS	-0.47871	NS
0.533	0.16088	NS	0.06382	NS	0.09858	NS
olixus group clade II						
0.09877			0	0	0	0
0.324	-1.06916	NS	-0.52903	NS	-0.7676	NS
0.021	**-2.53661**	*****, P < 0.001**	**-6.74165**	****, P < 0.02**	**-6.19779**	****, P < 0.02**
0.362	-0.69066	NS	-0.25181	NS	-0.43698	NS
0.753	1.2147	NS	0.31193	NS	0.66704	NS
0.307	**-2.25224**	****, P < 0.01**	**-6.22318**	****, P < 0.02**	**-5.62952**	**NS**
0.00976	0.9801	NS	0.21818	NS	0.49284	NS
0.841	0.39102	NS	1.65201	NS	1.47936	NS
0.5	-0.75445	NS	-0.75445	NS	-0.67466	NS
0.421	**-1.78062**	***, P < 0.05**	**-3.55814**	****, P < 0.02**	**-3.41316**	****, P < 0.02**

Supplementary Table 3. Tree topology test results

Tree	logL	deltaL	bp-RELL	p-KH	p-SH	p-WKH	p-WSH	c-ELW	p-AU
**1- IQTREE**	-21110.79339	402.66	0 -	0.0002 -	0.0009 -	0.0002 -	0.0008 -	7e-60 -	7.27e-82 -
**2- ASTRAL**	-21179.28605	471.16	0 -	0 -	0 -	0 -	0 -	1.45e-91 -	6.25e-52 -
**3- FASTREE**	-20744.1054	35.976	0.0433 +	0.208 +	0.737 +	0.208 +	0.644 +	0.0437 +	0.151 +
**4-PHYML**	**-20708.12964**	**0**	**0.335 +**	**0.578 +**	**1 +**	**0.578 +**	**0.972 +**	**0.336 +**	**0.645 +**
**5-MRBAYEs**	-20929.54483	221.42	0 -	0 -	0.0179 -	0 -	0 -	6.19e-49 -	2.67e-36 -

deltaL : logL difference from the maximal logl in the set.

bp-RELL : bootstrap proportion using RELL method (Kishino et al. 1990).

p-KH : p-value of one sided Kishino-Hasegawa test (1989).

p-SH : p-value of Shimodaira-Hasegawa test (2000).

p-WKH : p-value of weighted KH test.

p-WSH : p-value of weighted SH test.

c-ELW : Expected Likelihood Weight (Strimmer & Rambaut 2002).

p-AU : p-value of approximately unbiased (AU) test (Shimodaira, 2002).

*En rojo se señala los resultados de la topología de mayor credibilidad

Plus signs denote the 95% confidence sets.

Minus signs denote significant exclusion.

All tests performed 10000 resamplings using the RELL method.

Supplementary table 4. Results from the Structure Harvester Evanno method (K=20)

K	Reps	Mean LnP(K)	Stdev LnP(K)	Ln'(K)	|Ln''(K)|	Delta K
1	5	-12498.74	2.231143	—	—	—
2	5	-10175.84	40.054563	2322.9	1135.56	28.350328
3	5	-8988.5	283.19646	1187.34	39	0.137714
4	5	-7840.16	52.968274	1148.34	620.96	11.723244
5	5	-7312.78	151.83304	527.38	81.2	0.534798
6	5	-6704.2	56.265176	608.58	315.62	5.609509
7	5	-6411.24	63.911994	292.96	13.78	0.215609
8	5	-6104.5	110.296872	306.74	71.18	0.645349
9	5	-5868.94	228.712391	235.56	84.66	0.370159
10	5	-5548.72	96.376486	320.22	213	2.210083
11	5	-5441.5	70.742031	107.22	171.8	2.428542
12	5	-5162.48	72.728138	279.02	185.7	2.553345
13	5	-5069.16	105.464653	93.32	54.92	0.520743
14	5	-4920.92	39.617256	148.24	11.44	0.288763
15	5	-4784.12	58.948172	136.8	14.12	0.239532
16	5	-4661.44	69.077985	122.68	17.92	0.259417
17	5	-4556.68	31.278139	104.76	58.18	1.860085
18	5	-4510.1	58.148001	46.58	55.56	0.955493
19	5	-4407.96	55.662627	102.14	188.82	3.392222
20	5	-4494.64	311.14109	-86.68	—	—

Supplementary Table 5. GenBank accession numbers of eight loci analyzed in this study

ID	Gene	GenBank
TRNA	tRNA (Guanina (37) -N (1) metiltransferasa	ID submission: 2894370
PJH	Putative juvenile hormone inducible protein	ID submission: 2894372
CISP	Probable cytosolic iron sulfur protein assembly protein Ciao 1	PQ605815 - PQ606053
LSM	Lipoyl synthase mitocondrial	ID submission: 2894375
UPCA	Uncharacterized protein (Cell adhesion)	ID submission: 2894376
UPMETAL	Uncharacterized protein- metal ion binding	ID submission: 2894374
CYTB	Cytochrome b	ID submission: 2894379
28S	28S rRNA	PQ585846- PQ586084

Supplementary Table 6. Sequences ID amplified per locus.									
**28S**	**CISP**	**Cytb**	**LSM**	**PJH_V5**	**tRNA**	**UPCA_V5**	**UPMETAL**	**ALL**	**NUC**
-	100_Rrob	100_Rrob	100_Rrob	100_Rrob	100_Rrob	100_Rrob	100_Rrob	100_Rrob	100_Rrob
101_Rrob	101_Rrob	101_Rrob	101_Rrob	101_Rrob	101_Rrob	101_Rrob	101_Rrob	101_Rrob	101_Rrob
102_Rrob	102_Rrob	-	102_Rrob	102_Rrob	102_Rrob	102_Rrob	102_Rrob	102_Rrob	102_Rrob
103_Rrob	103_Rrob	103_Rrob	103_Rrob	103_Rrob	103_Rrob	103_Rrob	103_Rrob	103_Rrob	103_Rrob
-	104_Rrob	104_Rrob	104_Rrob	104_Rrob	104_Rrob	104_Rrob	-	104_Rrob	104_Rrob
105_Rrob	105_Rrob	105_Rrob	105_Rrob	105_Rrob	105_Rrob	105_Rrob	105_Rrob	105_Rrob	105_Rrob
-	106_Rrob	106_Rrob	106_Rrob	106_Rrob	106_Rrob	-	106_Rrob	106_Rrob	106_Rrob
107_Rrob	107_Rrob	107_Rrob	107_Rrob	107_Rrob	107_Rrob	-	107_Rrob	107_Rrob	107_Rrob
108_Rrob	-	108_Rrob	108_Rrob	108_Rrob	108_Rrob	108_Rrob	108_Rrob	108_Rrob	108_Rrob
-	109_Rnas	109_Rnas	109_Rnas	109_Rnas	-	109_Rnas	-	109_Rnas	109_Rnas
10_Rpro	-	10_Rpro	10_Rpro	10_Rpro	10_Rpro	10_Rpro	10_Rpro	10_Rpro	10_Rpro
110_Rnas	110_Rnas	110_Rnas	110_Rnas	110_Rnas	110_Rnas	110_Rnas	-	110_Rnas	110_Rnas
-	111_Rnas	111_Rnas	111_Rnas	111_Rnas	111_Rnas	-	-	111_Rnas	111_Rnas
112_Rnas	112_Rnas	112_Rnas	112_Rnas	112_Rnas	-	-	-	112_Rnas	112_Rnas
113_Rnas	-	113_Rnas	113_Rnas	113_Rnas	-	113_Rnas	-	113_Rnas	113_Rnas
114_Rnas	114_Rnas	114_Rnas	-	114_Rnas	-	114_Rnas	-	114_Rnas	114_Rnas
-	115_Rnas	115_Rnas	115_Rnas	115_Rnas	-	115_Rnas	-	115_Rnas	115_Rnas
-	116_Rnas	116_Rnas	116_Rnas	116_Rnas	116_Rnas	-	-	116_Rnas	116_Rnas
117_Rnas	117_Rnas	117_Rnas	117_Rnas	117_Rnas	117_Rnas	-	-	117_Rnas	117_Rnas
-	118_Rnas	118_Rnas	118_Rnas	118_Rnas	118_Rnas	118_Rnas	-	118_Rnas	118_Rnas
119_Rmon	119_Rmon	119_Rmon	119_Rmon	119_Rmon	119_Rmon	-	119_Rmon	119_Rmon	119_Rmon
11_Rpro	-	11_Rpro	11_Rpro	11_Rpro	11_Rpro	-	11_Rpro	11_Rpro	11_Rpro
-	120_Rmon	120_Rmon	120_Rmon	120_Rmon	120_Rmon	120_Rmon	120_Rmon	120_Rmon	120_Rmon
121_Rmon	121_Rmon	121_Rmon	121_Rmon	-	121_Rmon	121_Rmon	121_Rmon	121_Rmon	121_Rmon
-	122_Rmon	122_Rmon	122_Rmon	-	122_Rmon	122_Rmon	122_Rmon	122_Rmon	122_Rmon
123_Rmon	-	-	-	-	-	-	-		
-	124_Rmon	124_Rmon	124_Rmon	124_Rmon	124_Rmon	124_Rmon	124_Rmon	124_Rmon	124_Rmon
125_Rmon	125_Rmon	125_Rmon	125_Rmon	125_Rmon	125_Rmon	125_Rmon	125_Rmon	125_Rmon	125_Rmon
-	-	126_Rmon	126_Rmon	-	126_Rmon	126_Rmon	126_Rmon	126_Rmon	126_Rmon
-	127_Rmon	127_Rmon	127_Rmon	127_Rmon	127_Rmon	-	127_Rmon	127_Rmon	127_Rmon
128_Rmon	128_Rmon	128_Rmon	128_Rmon	128_Rmon	128_Rmon	-	128_Rmon	128_Rmon	128_Rmon
129_Rmon	129_Rmon	129_Rmon	129_Rmon	129_Rmon	129_Rmon	-	129_Rmon	129_Rmon	129_Rmon
-	12_Rpro	12_Rpro	12_Rpro	-	-	12_Rpro	12_Rpro	12_Rpro	12_Rpro
130_Rpro	-	-	-	130_Rpro	130_Rpro	130_Rpro	130_Rpro	130_Rpro	130_Rpro
132_Rpal	132_Rpal	132_Rpal	132_Rpal	132_Rpal	132_Rpal	132_Rpal	-	132_Rpal	132_Rpal
133_Rpal	-	133_Rpal	133_Rpal	133_Rpal	133_Rpal	-	-	133_Rpal	133_Rpal
134_Rpro	-	134_Rpro	134_Rpro	134_Rpro	134_Rpro	134_Rpro	134_Rpro	134_Rpro	134_Rpro
135_Rpal	-	135_Rpal	-	-	135_Rpal	-	-	135_Rpal	135_Rpal
136_Rpal	136_Rpal	136_Rpal	-	136_Rpal	136_Rpal	136_Rpal	-	136_Rpal	136_Rpal
137_Rpal	137_Rpal	137_Rpal	137_Rpal	137_Rpal	137_Rpal	137_Rpal	-	137_Rpal	137_Rpal
-	-	138_Rpal	138_Rpal	-	138_Rpal	-	-	138_Rpal	138_Rpal
-	139_Rpal	139_Rpal	139_Rpal	139_Rpal	139_Rpal	139_Rpal	-	139_Rpal	139_Rpal

28S	CISP	Cytb	LSM	PJH_V5	tRNA	UPCA_V5	UPMETAL	ALL	NUC
13_Rpro	13_Rpro	13_Rpro	13_Rpro	13_Rpro	13_Rpro	13_Rpro	-	13_Rpro	13_Rpro
140_Rpal	140_Rpal	140_Rpal	140_Rpal	140_Rpal	140_Rpal	140_Rpal	-	140_Rpal	140_Rpal
141_Rpal	-	141_Rpal	141_Rpal	141_Rpal	141_Rpal	141_Rpal	-	141_Rpal	141_Rpal
144_Rpal	-	-	-	-	-	-	-		
-	-	146_Rpal	146_Rpal	146_Rpal	146_Rpal	146_Rpal	-	146_Rpal	146_Rpal
-	-	149_Rpal	149_Rpal	-	149_Rpal	149_Rpal	-	149_Rpal	149_Rpal
14_Rpal	-	14_Rpal	14_Rpal	14_Rpal	14_Rpal	-	-	14_Rpal	14_Rpal
-	-	150_Rpal	150_Rpal	150_Rpal	150_Rpal	-	-	150_Rpal	150_Rpal
-	-	151_Rpal	151_Rpal	151_Rpal	151_Rpal	-	-	151_Rpal	151_Rpal
152_Rpal	-	152_Rpal	152_Rpal	152_Rpal	152_Rpal	-	-	152_Rpal	152_Rpal
-	-	-	153_Rpal	-	153_Rpal	-	-	153_Rpal	153_Rpal
-	-	154_Rpro	154_Rpro	154_Rpro	154_Rpro	154_Rpro	154_Rpro	154_Rpro	154_Rpro
-	-	155_Rpro	155_Rpro	155_Rpro	155_Rpro	155_Rpro	155_Rpro	155_Rpro	155_Rpro
-	-	156_Rpro	-	156_Rpro	156_Rpro	156_Rpro	-	156_Rpro	156_Rpro
157_Rpro	-	157_Rpro	157_Rpro	157_Rpro	157_Rpro	157_Rpro	157_Rpro	157_Rpro	157_Rpro
-	-	158_Rpro	158_Rpro	158_Rpro	158_Rpro	158_Rpro	158_Rpro	158_Rpro	158_Rpro
-	-	160_Rpro	160_Rpro	160_Rpro	160_Rpro	-	-	160_Rpro	160_Rpro
-	161_Rpro	161_Rpro	161_Rpro	161_Rpro	161_Rpro	161_Rpro	161_Rpro	161_Rpro	161_Rpro
-	-	162_Rpro	162_Rpro	162_Rpro	162_Rpro	162_Rpro	-	162_Rpro	162_Rpro
-	163_Rpro	163_Rpro	163_Rpro	163_Rpro	163_Rpro	163_Rpro	163_Rpro	163_Rpro	163_Rpro
-	-	164_Rpro	164_Rpro	164_Rpro	-	164_Rpro	164_Rpro	164_Rpro	164_Rpro
-	-	165_Rpro	-	165_Rpro	165_Rpro	165_Rpro	165_Rpro	165_Rpro	165_Rpro
-	-	166_Rpro	166_Rpro	166_Rpro	166_Rpro	166_Rpro	166_Rpro	166_Rpro	166_Rpro
167_Rpro	-	167_Rpro	167_Rpro	167_Rpro	167_Rpro	167_Rpro	167_Rpro	167_Rpro	167_Rpro
-	-	168_Rpro	168_Rpro	168_Rpro	168_Rpro	168_Rpro	168_Rpro	168_Rpro	168_Rpro
169_Rpal	-	169_Rpal	169_Rpal	169_Rpal	169_Rpal	-	-	169_Rpal	169_Rpal
-	16_Rpro	16_Rpro	-	-	-	-	16_Rpro	16_Rpro	16_Rpro
-	-	174_Rpal	174_Rpal	174_Rpal	174_Rpal	174_Rpal	-	174_Rpal	174_Rpal
-	-	178_Rpic	-	178_Rpic	-	-	-	178_Rpic	178_Rpic
-	-	179_Rpal	-	179_Rpal	179_Rpal	-	-	179_Rpal	179_Rpal
17_Rpro	-	17_Rpro	-	-	-	-	17_Rpro	17_Rpro	17_Rpro
-	-	182_Rpic	-	182_Rpic	182_Rpic	-	-	182_Rpic	182_Rpic
-	-	-	-	183_Rpic	183_Rpic	-	-	183_Rpic	183_Rpic
-	185_Rpal	-	185_Rpal	185_Rpal	185_Rpal	-	-	185_Rpal	185_Rpal
-	186_Rpal	186_Rpal	186_Rpal	-	186_Rpal	-	-	186_Rpal	186_Rpal
-	-	187_Rpal	187_Rpal	-	187_Rpal	-	-	187_Rpal	187_Rpal
-	-	188_Rpal	188_Rpal	-	188_Rpal	-	-	188_Rpal	188_Rpal
-	18_Rpro	-	-	-	-	-	18_Rpro	18_Rpro	18_Rpro
-	195_Rpro	-	-	-	-	-	-		
-	-	196_Rpro	196_Rpro	196_Rpro	196_Rpro	196_Rpro	196_Rpro	196_Rpro	196_Rpro
-	197_Rpro	197_Rpro	197_Rpro	197_Rpro	-	-	197_Rpro	197_Rpro	197_Rpro
-	-	198_Rpro	198_Rpro	198_Rpro	198_Rpro	-	198_Rpro	198_Rpro	198_Rpro
-	-	199_Rpro	199_Rpro	199_Rpro	199_Rpro	199_Rpro	199_Rpro	199_Rpro	199_Rpro

28S	CISP	Cytb	LSM	PJH_V5	tRNA	UPCA_V5	UPMETAL	ALL	NUC
-	19_Rpro	-	-	-	-	-	-		
-	-	1_Rpro	1_Rpro	1_Rpro	1_Rpro	1_Rpro	-	1_Rpro	1_Rpro
-	-	200_Rpro	200_Rpro	200_Rpro	200_Rpro	200_Rpro	200_Rpro	200_Rpro	200_Rpro
-	202_Rpro	202_Rpro	202_Rpro	202_Rpro	202_Rpro	202_Rpro	202_Rpro	202_Rpro	202_Rpro
203_Rpro	-	203_Rpro	203_Rpro	203_Rpro	203_Rpro	203_Rpro	203_Rpro	203_Rpro	203_Rpro
-	-	210_Rpro	210_Rpro	210_Rpro	-	210_Rpro	210_Rpro	210_Rpro	210_Rpro
-	211_Rpro	211_Rpro	211_Rpro	211_Rpro	211_Rpro	211_Rpro	211_Rpro	211_Rpro	211_Rpro
-	-	212_Rpro	-	212_Rpro	212_Rpro	-	-	212_Rpro	212_Rpro
-	213_Rpro	213_Rpro	213_Rpro	213_Rpro	213_Rpro	-	-	213_Rpro	213_Rpro
-	-	214_Rpro	214_Rpro	-	-	-	-	214_Rpro	214_Rpro
-	-	215_Rpro	215_Rpro	-	-	-	-	215_Rpro	215_Rpro
-	216_Rpro	216_Rpro	216_Rpro	216_Rpro	-	216_Rpro	216_Rpro	216_Rpro	216_Rpro
-	-	218_Rpro	218_Rpro	218_Rpro	-	218_Rpro	218_Rpro	218_Rpro	218_Rpro
-	219_Rpro	219_Rpro	-	219_Rpro	-	219_Rpro	219_Rpro	219_Rpro	219_Rpro
-	-	21_Rpro	-	-	-	-	-		
-	-	220_Rpro	220_Rpro	220_Rpro	-	220_Rpro	220_Rpro	220_Rpro	220_Rpro
-	221_Rpro	221_Rpro	221_Rpro	-	-	221_Rpro	221_Rpro	221_Rpro	221_Rpro
-	-	222_Rpro	222_Rpro	-	-	222_Rpro	-	222_Rpro	222_Rpro
-	-	223_Rpro	-	-	-	223_Rpro	223_Rpro	223_Rpro	223_Rpro
224_Part	-	-	-	224_Part	-	224_Part	224_Part	224_Part	224_Part
225_Part	-	-	225_Part	225_Part	225_Part	225_Part	225_Part	225_Part	225_Part
226_Part	-	-	226_Part	226_Part	226_Part	-	226_Part	226_Part	226_Part
227_Part	227_Part	-	227_Part	227_Part	227_Part	227_Part	-	227_Part	227_Part
228_Part	-	-	-	228_Part	-	228_Part	-	228_Part	228_Part
229_Part	-	-	229_Part	229_Part	-	229_Part	229_Part	229_Part	229_Part
-	22_Rpro	-	-	-	22_Rpro	22_Rpro	22_Rpro	22_Rpro	22_Rpro
230_Part	-	-	230_Part	230_Part	230_Part	230_Part	230_Part	230_Part	230_Part
231_Part	-	-	-	231_Part	-	231_Part	231_Part	231_Part	231_Part
-	232_Part	-	232_Part	232_Part	232_Part	232_Part	232_Part	232_Part	232_Part
233_Part	-	-	233_Part	233_Part	233_Part	233_Part	233_Part	233_Part	233_Part
234_Part	-	-	234_Part	234_Part	-	234_Part	-	234_Part	234_Part
235_Rpal	-	235_Rpal	235_Rpal	235_Rpal	235_Rpal	235_Rpal	-	235_Rpal	235_Rpal
237_Rpal	-	237_Rpal	237_Rpal	237_Rpal	237_Rpal	237_Rpal	237_Rpal	237_Rpal	237_Rpal
-	-	239_Rpal	239_Rpal	239_Rpal	239_Rpal	239_Rpal	-	239_Rpal	239_Rpal
-	23_Rpro	-	-	-	-	-	23_Rpro	23_Rpro	23_Rpro
-	241_Rpal	241_Rpal	241_Rpal	241_Rpal	241_Rpal	241_Rpal	-	241_Rpal	241_Rpal
-	-	243_Rpal	-	-	-	-	-		
-	245_Rpal	245_Rpal	245_Rpal	245_Rpal	245_Rpal	-	-	245_Rpal	245_Rpal
-	247_Rpal	247_Rpal	247_Rpal	247_Rpal	247_Rpal	247_Rpal	-	247_Rpal	247_Rpal
-	249_Rpal	-	249_Rpal	-	249_Rpal	-	249_Rpal	249_Rpal	249_Rpal
24_Rpro	24_Rpro	24_Rpro	24_Rpro	-	-	-	-	24_Rpro	24_Rpro
251_Rpal	251_Rpal	251_Rpal	251_Rpal	251_Rpal	251_Rpal	-	-	251_Rpal	251_Rpal
-	253_Rpal	253_Rpal	253_Rpal	253_Rpal	253_Rpal	-	-	253_Rpal	253_Rpal

28S	CISP	Cytb	LSM	PJH_V5	tRNA	UPCA_V5	UPMETAL	ALL	NUC
255_Rpal	255_Rpal	-	255_Rpal	255_Rpal	255_Rpal	255_Rpal	255_Rpal	255_Rpal	255_Rpal
257_Rpal	-	257_Rpal	257_Rpal	257_Rpal	257_Rpal	257_Rpal	-	257_Rpal	257_Rpal
-	259_Rpal	259_Rpal	259_Rpal	259_Rpal	259_Rpal	259_Rpal	-	259_Rpal	259_Rpal
-	-	-	260_Rpal	260_Rpal	260_Rpal	-	-	260_Rpal	260_Rpal
-	-	262_Rpal	262_Rpal	-	262_Rpal	-	-	262_Rpal	262_Rpal
263_Rpal	263_Rpal	263_Rpal	263_Rpal	263_Rpal	263_Rpal	-	-	263_Rpal	263_Rpal
-	-	265_Rpal	265_Rpal	-	-	-	-	265_Rpal	265_Rpal
-	266_Rpal	266_Rpal	266_Rpal	266_Rpal	-	-	-	266_Rpal	266_Rpal
-	-	-	269_Rpal	-	269_Rpal	-	-	269_Rpal	269_Rpal
-	26_Rpro	26_Rpro	26_Rpro	26_Rpro	26_Rpro	26_Rpro	26_Rpro	26_Rpro	26_Rpro
270_Recu	270_Recu	270_Recu	270_Recu	270_Recu	-	-	270_Recu	270_Recu	270_Recu
-	-	-	-	-	271_Recu	-	-		
272_Recu	272_Recu	272_Recu	272_Recu	272_Recu	-	272_Recu	-	272_Recu	272_Recu
-	-	-	-	-	273_Recu	-	-		
274_Recu	274_Recu	274_Recu	274_Recu	274_Recu	274_Recu	-	274_Recu	274_Recu	274_Recu
276_Recu	276_Recu	276_Recu	-	276_Recu	276_Recu	276_Recu	276_Recu	276_Recu	276_Recu
278_Recu	278_Recu	278_Recu	278_Recu	278_Recu	278_Recu	278_Recu	278_Recu	278_Recu	278_Recu
280_Rnas	280_Rnas	280_Rnas	280_Rnas	280_Rnas	280_Rnas	-	280_Rnas	280_Rnas	280_Rnas
282_Rnas	282_Rnas	-	282_Rnas	282_Rnas	282_Rnas	282_Rnas	282_Rnas	282_Rnas	282_Rnas
284_Rnas	-	284_Rnas	284_Rnas	-	284_Rnas	-	284_Rnas	284_Rnas	284_Rnas
287_Rnas	287_Rnas	287_Rnas	287_Rnas	287_Rnas	287_Rnas	287_Rnas	287_Rnas	287_Rnas	287_Rnas
289_Rnas	-	-	289_Rnas	289_Rnas	289_Rnas	-	289_Rnas	289_Rnas	289_Rnas
-	-	290_Rnas	-	-	-	-	-		
291_Rnas	-	-	-	-	291_Rnas	-	291_Rnas	291_Rnas	291_Rnas
292_Rnas	292_Rnas	292_Rnas	292_Rnas	292_Rnas	292_Rnas	-	292_Rnas	292_Rnas	292_Rnas
-	294_Rmil	-	294_Rmil	294_Rmil	-	-	-	294_Rmil	294_Rmil
295_Rmil	295_Rmil	295_Rmil	295_Rmil	295_Rmil	-	-	295_Rmil	295_Rmil	295_Rmil
-	-	-	-	-	296_Rmil	-	-		
-	-	-	297_Rmil	297_Rmil	-	-	-	297_Rmil	297_Rmil
298_Rmil	298_Rmil	-	298_Rmil	298_Rmil	298_Rmil	298_Rmil	298_Rmil	298_Rmil	298_Rmil
299_Rmil	299_Rmil	299_Rmil	299_Rmil	299_Rmil	299_Rmil	-	299_Rmil	299_Rmil	299_Rmil
-	-	29_Rpro	-	-	-	-	-		
-	-	-	2_Rpro	2_Rpro	2_Rpro	-	2_Rpro	2_Rpro	2_Rpro
301_Rmil	301_Rmil	301_Rmil	301_Rmil	301_Rmil	-	301_Rmil	-	301_Rmil	301_Rmil
303_Rmil	303_Rmil	303_Rmil	303_Rmil	303_Rmil	-	303_Rmil	-	303_Rmil	303_Rmil
-	-	-	-	-	304_Rmil	-	-		
305_Rmil	-	305_Rmil	305_Rmil	305_Rmil	-	-	305_Rmil	305_Rmil	305_Rmil
-	-	-	-	-	306_Rmil	-	-		
-	-	307_Rrob	307_Rrob	307_Rrob	307_Rrob	307_Rrob	307_Rrob	307_Rrob	307_Rrob
-	308_Rrob	308_Rrob	308_Rrob	308_Rrob	308_Rrob	308_Rrob	308_Rrob	308_Rrob	308_Rrob
-	-	30_Rpro	-	-	30_Rpro	30_Rpro	30_Rpro	30_Rpro	30_Rpro
310_Rrob	-	-	310_Rrob	310_Rrob	310_Rrob	310_Rrob	310_Rrob	310_Rrob	310_Rrob
-	-	311_Rrob	-	-	-	-	-		

28S	CISP	Cytb	LSM	PJH_V5	tRNA	UPCA_V5	UPMETAL	ALL	NUC
312_Rrob	-	312_Rrob	312_Rrob	312_Rrob	312_Rrob	312_Rrob	312_Rrob	312_Rrob	312_Rrob
314_Rrob	-	314_Rrob	314_Rrob	314_Rrob	-	314_Rrob	314_Rrob	314_Rrob	314_Rrob
316_Rrob	316_Rrob	316_Rrob	-	316_Rrob	316_Rrob	316_Rrob	316_Rrob	316_Rrob	316_Rrob
-	-	-	317_Rrob	-	-	-	-		
318_Rrob	-	318_Rrob	318_Rrob	318_Rrob	318_Rrob	-	-	318_Rrob	318_Rrob
-	31_Rpro	-	-	31_Rpro	-	-	31_Rpro	31_Rpro	31_Rpro
-	-	-	321_Rbre	321_Rbre	321_Rbre	-	-	321_Rbre	321_Rbre
-	-	323_Rbre	-	323_Rbre	323_Rbre	-	-	323_Rbre	323_Rbre
-	-	325_Rbre	325_Rbre	325_Rbre	325_Rbre	-	-	325_Rbre	325_Rbre
-	327_Rbre	327_Rbre	-	327_Rbre	327_Rbre	-	-	327_Rbre	327_Rbre
-	-	329_Rbre	329_Rbre	329_Rbre	329_Rbre	-	-	329_Rbre	329_Rbre
-	32_Rpro	-	-	-	-	-	32_Rpro	32_Rpro	32_Rpro
-	-	331_Rbre	331_Rbre	331_Rbre	331_Rbre	-	-	331_Rbre	331_Rbre
332_Rsta	-	332_Rsta	332_Rsta	332_Rsta	-	-	-	332_Rsta	332_Rsta
-	-	-	-	-	333_Rsta	-	333_Rsta	333_Rsta	333_Rsta
-	-	-	334_Rsta	334_Rsta	-	-	-	334_Rsta	334_Rsta
335_Rsta	-	335_Rsta	335_Rsta	335_Rsta	335_Rsta	-	-	335_Rsta	335_Rsta
337_Rsta	-	337_Rsta	337_Rsta	337_Rsta	337_Rsta	-	-	337_Rsta	337_Rsta
-	-	-	-	-	-	-	338_Rsta		
339_Rsta	-	339_Rsta	339_Rsta	339_Rsta	339_Rsta	-	-	339_Rsta	339_Rsta
-	33_Rpro	-	-	33_Rpro	-	-	-	33_Rpro	33_Rpro
-	-	-	-	-	-	-	340_Rsta		
341_Rsta	-	341_Rsta	341_Rsta	341_Rsta	341_Rsta	341_Rsta	-	341_Rsta	341_Rsta
343_Rpro	343_Rpro	-	343_Rpro	343_Rpro	343_Rpro	343_Rpro	-	343_Rpro	343_Rpro
345_Rpro	345_Rpro	345_Rpro	345_Rpro	345_Rpro	345_Rpro	345_Rpro	-	345_Rpro	345_Rpro
-	-	-	-	346_Rpro	-	-	346_Rpro	346_Rpro	346_Rpro
347_Rpro	347_Rpro	347_Rpro	347_Rpro	-	347_Rpro	347_Rpro	-	347_Rpro	347_Rpro
-	-	-	-	-	-	-	348_Rpro		
-	-	349_Rpro	349_Rpro	-	349_Rpro	349_Rpro	-	349_Rpro	349_Rpro
351_Rpro	351_Rpro	351_Rpro	351_Rpro	351_Rpro	351_Rpro	351_Rpro	-	351_Rpro	351_Rpro
353_Rneg	-	-	353_Rneg	353_Rneg	-	353_Rneg	-	353_Rneg	353_Rneg
-	-	354_Rneg	-	-	354_Rneg	-	354_Rneg	354_Rneg	354_Rneg
355_Rneg	-	355_Rneg	355_Rneg	355_Rneg	-	-	-	355_Rneg	355_Rneg
357_Rneg	357_Rneg	357_Rneg	357_Rneg	357_Rneg	-	357_Rneg	-	357_Rneg	357_Rneg
359_Rneg	359_Rneg	359_Rneg	359_Rneg	359_Rneg	-	359_Rneg	359_Rneg	359_Rneg	359_Rneg
361_Rneg	-	361_Rneg	361_Rneg	361_Rneg	-	361_Rneg	-	361_Rneg	361_Rneg
-	-	-	-	-	362_Rneg	-	-		
363_Rnas	363_Rnas	363_Rnas	-	363_Rnas	-	363_Rnas	363_Rnas	363_Rnas	363_Rnas
-	-	-	365_Rnas	-	-	365_Rnas	-	365_Rnas	365_Rnas
-	-	-	-	-	-	-	366_Rnas		
-	367_Rnas	-	367_Rnas	367_Rnas	-	367_Rnas	-	367_Rnas	367_Rnas
-	-	368_Rnas	-	-	368_Rnas	-	-	368_Rnas	368_Rnas
369_Rnas	-	-	369_Rnas	369_Rnas	-	-	-	369_Rnas	369_Rnas

28S	CISP	Cytb	LSM	PJH_V5	tRNA	UPCA_V5	UPMETAL	ALL	NUC
-	-	370_Rnas	-	-	370_Rnas	-	-	370_Rnas	370_Rnas
371_Rnas	-	-	371_Rnas	371_Rnas	-	371_Rnas	-	371_Rnas	371_Rnas
-	-	-	-	-	372_Rnas	-	372_Rnas	372_Rnas	372_Rnas
-	-	373_Rbre	-	-	-	-	-		
-	-	374_Rbre	374_Rbre	374_Rbre	-	-	-	374_Rbre	374_Rbre
375_Rbre	-	375_Rbre	-	375_Rbre	-	-	-	375_Rbre	375_Rbre
-	376_Rbre	376_Rbre	376_Rbre	-	376_Rbre	-	-	376_Rbre	376_Rbre
-	-	378_Rbre	378_Rbre	378_Rbre	378_Rbre	-	-	378_Rbre	378_Rbre
-	380_Rbre	380_Rbre	380_Rbre	380_Rbre	380_Rbre	-	-	380_Rbre	380_Rbre
-	-	381_Rbre	381_Rbre	381_Rbre	-	-	-	381_Rbre	381_Rbre
-	-	-	-	-	382_Rbre	-	-		
383_Rcol	383_Rcol	-	383_Rcol	-	383_Rcol	-	-	383_Rcol	383_Rcol
-	385_Rcol	-	385_Rcol	385_Rcol	385_Rcol	-	-	385_Rcol	385_Rcol
387_Rcol	387_Rcol	-	387_Rcol	-	387_Rcol	-	-	387_Rcol	387_Rcol
-	-	-	388_Rpic	388_Rpic	388_Rpic	-	-	388_Rpic	388_Rpic
390_Rpic	-	-	-	390_Rpic	390_Rpic	-	-	390_Rpic	390_Rpic
-	-	-	391_Rpic	-	-	-	-		
-	394_Rpic	-	394_Rpic	394_Rpic	394_Rpic	-	-	394_Rpic	394_Rpic
-	396_Rpic	-	396_Rpic	396_Rpic	-	-	-	396_Rpic	396_Rpic
-	-	-	399_Rcol	399_Rcol	-	-	399_Rcol	399_Rcol	399_Rcol
-	-	-	3_Rpro	3_Rpro	3_Rpro	3_Rpro	3_Rpro	3_Rpro	3_Rpro
-	-	-	-	-	-	400_Rcol	-		
-	-	401_Rcol	-	401_Rcol	401_Rcol	-	-	401_Rcol	401_Rcol
-	-	403_Rneg	-	-	-	-	-		
-	404_Rneg	-	-	404_Rneg	404_Rneg	-	404_Rneg	404_Rneg	404_Rneg
-	-	405_Rneg	405_Rneg	405_Rneg	-	-	-	405_Rneg	405_Rneg
-	406_Rneg	-	-	-	406_Rneg	406_Rneg	-	406_Rneg	406_Rneg
-	407_Rneg	407_Rneg	407_Rneg	407_Rneg	-	-	-	407_Rneg	407_Rneg
409_Rneg	409_Rneg	409_Rneg	409_Rneg	409_Rneg	409_Rneg	409_Rneg	409_Rneg	409_Rneg	409_Rneg
-	411_Rneg	411_Rneg	411_Rneg	411_Rneg	411_Rneg	411_Rneg	411_Rneg	411_Rneg	411_Rneg
-	-	413_Recu	-	-	413_Recu	413_Recu	-	413_Recu	413_Recu
-	414_Recu	414_Recu	414_Recu	-	-	-	-	414_Recu	414_Recu
-	415_Recu	-	415_Recu	-	-	415_Recu	-	415_Recu	415_Recu
416_Recu	-	-	-	416_Recu	-	-	-	416_Recu	416_Recu
-	417_Recu	417_Recu	417_Recu	417_Recu	417_Recu	417_Recu	-	417_Recu	417_Recu
-	419_Recu	419_Recu	-	-	419_Recu	-	-	419_Recu	419_Recu
-	-	-	41_Rcol	41_Rcol	41_Rcol	41_Rcol	41_Rcol	41_Rcol	41_Rcol
-	-	-	-	420_Recu	-	-	420_Recu	420_Recu	420_Recu
-	-	421_Recu	-	-	421_Recu	-	-	421_Recu	421_Recu
-	422_Recu	422_Recu	422_Recu	422_Recu	-	422_Recu	-	422_Recu	422_Recu
-	423_Rpal	423_Rpal	-	423_Rpal	423_Rpal	-	-	423_Rpal	423_Rpal
-	-	424_Rpal	-	424_Rpal	424_Rpal	424_Rpal	-	424_Rpal	424_Rpal
-	426_Rpal	426_Rpal	-	-	426_Rpal	426_Rpal	426_Rpal	426_Rpal	426_Rpal

28S	CISP	Cytb	LSM	PJH_V5	tRNA	UPCA_V5	UPMETAL	ALL	NUC
-	-	428_Rpal	-	428_Rpal	428_Rpal	428_Rpal	-	428_Rpal	428_Rpal
429_Rpal	429_Rpal	429_Rpal	-	429_Rpal	429_Rpal	-	-	429_Rpal	429_Rpal
42_Rcol	-	42_Rcol	42_Rcol	42_Rcol	42_Rcol	42_Rcol	42_Rcol	42_Rcol	42_Rcol
-	-	430_Rpro	430_Rpro	430_Rpro	430_Rpro	430_Rpro	430_Rpro	430_Rpro	430_Rpro
431_Rpro	431_Rpro	-	-	-	-	-	-	431_Rpro	431_Rpro
-	432_Rpro	432_Rpro	432_Rpro	432_Rpro	432_Rpro	432_Rpro	432_Rpro	432_Rpro	432_Rpro
433_Rpro	-	-	-	-	-	-	-		
-	-	434_Rpro	434_Rpro	434_Rpro	434_Rpro	434_Rpro	434_Rpro	434_Rpro	434_Rpro
436_Rpro	436_Rpro	-	-	436_Rpro	436_Rpro	436_Rpro	436_Rpro	436_Rpro	436_Rpro
438_Rpro	438_Rpro	438_Rpro	438_Rpro	-	-	438_Rpro	438_Rpro	438_Rpro	438_Rpro
-	-	-	43_Rcol	43_Rcol	43_Rcol	-	43_Rcol	43_Rcol	43_Rcol
440_Rrob	440_Rrob	440_Rrob	440_Rrob	440_Rrob	440_Rrob	440_Rrob	440_Rrob	440_Rrob	440_Rrob
-	-	-	445_Rrob	445_Rrob	445_Rrob	445_Rrob	-	445_Rrob	445_Rrob
446_Rrob	-	446_Rrob	446_Rrob	446_Rrob	446_Rrob	446_Rrob	446_Rrob	446_Rrob	446_Rrob
448_Rrob	-	448_Rrob	448_Rrob	-	448_Rrob	448_Rrob	448_Rrob	448_Rrob	448_Rrob
453_Rmar	453_Rmar	-	-	453_Rmar	453_Rmar	453_Rmar	453_Rmar	453_Rmar	453_Rmar
-	-	-	454_Rmar	454_Rmar	-	454_Rmar	454_Rmar	454_Rmar	454_Rmar
-	-	455_Rmar	-	-	455_Rmar	-	-	455_Rmar	455_Rmar
456_Rmar	-	456_Rmar	456_Rmar	456_Rmar	-	456_Rmar	456_Rmar	456_Rmar	456_Rmar
458_Rmar	-	458_Rmar	458_Rmar	458_Rmar	-	458_Rmar	-	458_Rmar	458_Rmar
460_Rmil	-	-	460_Rmil	460_Rmil	-	460_Rmil	-	460_Rmil	460_Rmil
462_Rmil	-	-	-	-	-	462_Rmil	-	462_Rmil	462_Rmil
464_Rmil	-	-	464_Rmil	464_Rmil	-	464_Rmil	-	464_Rmil	464_Rmil
-	-	465_Rmil	-	-	-	-	-		
-	-	-	466_Rmil	466_Rmil	466_Rmil	466_Rmil	-	466_Rmil	466_Rmil
467_Rmil	467_Rmil	-	467_Rmil	467_Rmil	467_Rmil	-	-	467_Rmil	467_Rmil
469_Rmon	-	469_Rmon	-	469_Rmon	469_Rmon	469_Rmon	469_Rmon	469_Rmon	469_Rmon
471_Rmon	471_Rmon	471_Rmon	471_Rmon	471_Rmon	471_Rmon	471_Rmon	471_Rmon	471_Rmon	471_Rmon
-	474_Rmon	474_Rmon	-	474_Rmon	-	-	-	474_Rmon	474_Rmon
-	-	475_Rmon	475_Rmon	475_Rmon	-	475_Rmon	475_Rmon	475_Rmon	475_Rmon
-	476_Rmon	-	-	-	-	-	-	476_Rmon	476_Rmon
-	478_Rmon	478_Rmon	478_Rmon	478_Rmon	478_Rmon	478_Rmon	478_Rmon	478_Rmon	478_Rmon
479_Rnei	479_Rnei	-	479_Rnei	479_Rnei	-	479_Rnei	479_Rnei	479_Rnei	479_Rnei
47_Rpro	47_Rpro	47_Rpro	47_Rpro	47_Rpro	47_Rpro	-	47_Rpro	47_Rpro	47_Rpro
481_Rnei	-	-	481_Rnei	481_Rnei	481_Rnei	481_Rnei	-	481_Rnei	481_Rnei
483_Rnei	-	-	483_Rnei	483_Rnei	-	483_Rnei	-	483_Rnei	483_Rnei
485_Rnei	-	-	-	-	-	-	-		
-	486_Rnei	-	486_Rnei	486_Rnei	-	486_Rnei	-	486_Rnei	486_Rnei
-	-	-	487_Rnei	487_Rnei	-	487_Rnei	-	487_Rnei	487_Rnei
-	-	-	-	-	488_Rnei	-	-		
48_Rpro	-	48_Rpro	48_Rpro	48_Rpro	-	-	-	48_Rpro	48_Rpro
-	-	490_Rsta	-	490_Rsta	490_Rsta	-	-	490_Rsta	490_Rsta
-	492_Rsta	492_Rsta	492_Rsta	492_Rsta	-	492_Rsta	492_Rsta	492_Rsta	492_Rsta

28S	CISP	Cytb	LSM	PJH_V5	tRNA	UPCA_V5	UPMETAL	ALL	NUC
-	494_Rsta	494_Rsta	494_Rsta	494_Rsta	494_Rsta	-	-	494_Rsta	494_Rsta
-	-	-	495_Rsta	-	495_Rsta	-	-	495_Rsta	495_Rsta
-	-	496_Rsta	-	-	-	-	-		
-	-	497_Rsta	497_Rsta	497_Rsta	497_Rsta	-	-	497_Rsta	497_Rsta
-	-	-	499_Pter	499_Pter	499_Pter	499_Pter	-	499_Pter	499_Pter
49_Rpro	49_Rpro	49_Rpro	49_Rpro	49_Rpro	49_Rpro	49_Rpro	49_Rpro	49_Rpro	49_Rpro
-	-	4_Rpro	4_Rpro	4_Rpro	4_Rpro	4_Rpro	-	4_Rpro	4_Rpro
500_Pter	500_Pter	-	-	-	-	-	500_Pter	500_Pter	500_Pter
-	502_Pter	-	502_Pter	-	502_Pter	502_Pter	502_Pter	502_Pter	502_Pter
-	504_Pter	-	504_Pter	504_Pter	-	504_Pter	504_Pter	504_Pter	504_Pter
-	-	-	-	-	-	505_Pter	-		
-	506_Pter	-	506_Pter	506_Pter	-	-	-	506_Pter	506_Pter
-	-	-	-	507_Pter	507_Pter	507_Pter	-	507_Pter	507_Pter
-	-	-	508_Pter	-	-	-	508_Pter	508_Pter	508_Pter
50_Rpro	50_Rpro	50_Rpro	50_Rpro	50_Rpro	50_Rpro	50_Rpro	-	50_Rpro	50_Rpro
510_Pcor	-	510_Pcor	510_Pcor	510_Pcor	510_Pcor	510_Pcor	-	510_Pcor	510_Pcor
-	-	511_Pcor	511_Pcor	511_Pcor	511_Pcor	511_Pcor	511_Pcor	511_Pcor	511_Pcor
-	512_Pcor	512_Pcor	512_Pcor	512_Pcor	512_Pcor	512_Pcor	512_Pcor	512_Pcor	512_Pcor
514_Part	-	514_Part	514_Part	514_Part	514_Part	514_Part	-	514_Part	514_Part
515_Part	515_Part	515_Part	515_Part	515_Part	515_Part	515_Part	-	515_Part	515_Part
-	-	516_Part	516_Part	516_Part	516_Part	516_Part	-	516_Part	516_Part
-	-	517_Part	517_Part	-	-	517_Part	-	517_Part	517_Part
-	-	518_Part	-	-	-	-	-		
519_Rpro	-	-	-	-	-	519_Rpro	519_Rpro	519_Rpro	519_Rpro
51_Rpro	-	51_Rpro	51_Rpro	51_Rpro	51_Rpro	51_Rpro	51_Rpro	51_Rpro	51_Rpro
-	-	520_Rpro	-	520_Rpro	520_Rpro	-	-	520_Rpro	520_Rpro
521_Rpro	-	521_Rpro	-	-	-	521_Rpro	521_Rpro	521_Rpro	521_Rpro
-	522_Rpro	-	522_Rpro	-	-	-	-	522_Rpro	522_Rpro
-	-	-	-	-	-	-	523_Rpro		
-	-	524_Rpro	-	524_Rpro	524_Rpro	-	-	524_Rpro	524_Rpro
525_Rpro	-	525_Rpro	525_Rpro	525_Rpro	-	525_Rpro	525_Rpro	525_Rpro	525_Rpro
528_Rpro	528_Rpro	528_Rpro	528_Rpro	528_Rpro	-	528_Rpro	528_Rpro	528_Rpro	528_Rpro
52_Rpro	52_Rpro	52_Rpro	52_Rpro	52_Rpro	52_Rpro	52_Rpro	52_Rpro	52_Rpro	52_Rpro
-	-	-	-	-	-	530_Rrob	530_Rrob	530_Rrob	530_Rrob
-	-	-	531_Rrob	-	-	-	531_Rrob	531_Rrob	531_Rrob
-	-	532_Rrob	532_Rrob	-	-	532_Rrob	532_Rrob	532_Rrob	532_Rrob
-	533_Rrob	533_Rrob	-	533_Rrob	-	533_Rrob	-	533_Rrob	533_Rrob
534_Rrob	-	-	-	-	-	-	-		
-	-	535_Rrob	-	535_Rrob	535_Rrob	535_Rrob	535_Rrob	535_Rrob	535_Rrob
536_Rrob	-	-	-	-	-	536_Rrob	-	536_Rrob	536_Rrob
-	537_Rrob	537_Rrob	537_Rrob	537_Rrob	537_Rrob	-	-	537_Rrob	537_Rrob
-	-	-	538_Rrob	-	-	538_Rrob	538_Rrob	538_Rrob	538_Rrob
-	539_Rrob	539_Rrob	-	-	539_Rrob	-	-	539_Rrob	539_Rrob

28S	CISP	Cytb	LSM	PJH_V5	tRNA	UPCA_V5	UPMETAL	ALL	NUC
-	-	-	53_Rpro	53_Rpro	53_Rpro	53_Rpro	-	53_Rpro	53_Rpro
-	-	-	540_Rrob	-	-	540_Rrob	540_Rrob	540_Rrob	540_Rrob
-	541_Rrob	541_Rrob	-	-	541_Rrob	-	-	541_Rrob	541_Rrob
543_Rrob	543_Rrob	543_Rrob	543_Rrob	543_Rrob	543_Rrob	-	543_Rrob	543_Rrob	543_Rrob
-	-	-	-	-	-	544_Recu	-		
-	-	545_Recu	-	-	545_Recu	-	-	545_Recu	545_Recu
-	-	546_Recu	546_Recu	546_Recu	-	546_Recu	-	546_Recu	546_Recu
-	-	548_Recu	548_Recu	-	-	548_Recu	-	548_Recu	548_Recu
-	-	-	-	-	549_Recu	-	-		
-	-	54_Rpro	54_Rpro	-	-	54_Rpro	54_Rpro	54_Rpro	54_Rpro
-	-	-	550_Recu	-	-	-	-		
-	-	-	-	555_Rrob	-	-	-		
-	556_Rrob	-	-	556_Rrob	-	556_Rrob	556_Rrob	556_Rrob	556_Rrob
-	-	55_Rpro	55_Rpro	-	55_Rpro	55_Rpro	-	55_Rpro	55_Rpro
-	-	-	561_Rrob	-	-	561_Rrob	-	561_Rrob	561_Rrob
-	-	-	-	-	-	562_Rpro	-		
-	563_Rpro	-	563_Rpro	-	-	-	563_Rpro	563_Rpro	563_Rpro
564_Rpro	-	564_Rpro	564_Rpro	-	-	564_Rpro	-	564_Rpro	564_Rpro
-	565_Rpro	-	-	-	-	-	-		
566_Rpro	-	-	-	-	-	-	-		
-	567_Rpro	-	567_Rpro	-	-	567_Rpro	567_Rpro	567_Rpro	567_Rpro
-	568_Rpro	568_Rpro	568_Rpro	568_Rpro	568_Rpro	568_Rpro	568_Rpro	568_Rpro	568_Rpro
56_Rpro	-	56_Rpro	56_Rpro	56_Rpro	56_Rpro	56_Rpro	56_Rpro	56_Rpro	56_Rpro
570_Rpro	-	570_Rpro	570_Rpro	570_Rpro	570_Rpro	-	570_Rpro	570_Rpro	570_Rpro
-	572_Rpro	572_Rpro	572_Rpro	572_Rpro	572_Rpro	572_Rpro	572_Rpro	572_Rpro	572_Rpro
-	574_Rpro	574_Rpro	574_Rpro	574_Rpro	574_Rpro	-	574_Rpro	574_Rpro	574_Rpro
576_Rpro	576_Rpro	576_Rpro	576_Rpro	576_Rpro	576_Rpro	576_Rpro	576_Rpro	576_Rpro	576_Rpro
578_Rpro	578_Rpro	578_Rpro	578_Rpro	-	578_Rpro	578_Rpro	578_Rpro	578_Rpro	578_Rpro
-	-	-	57_Rpro	-	-	57_Rpro	-	57_Rpro	57_Rpro
580_Rpro	-	580_Rpro	580_Rpro	580_Rpro	580_Rpro	580_Rpro	580_Rpro	580_Rpro	580_Rpro
582_Rpro	582_Rpro	582_Rpro	582_Rpro	582_Rpro	582_Rpro	582_Rpro	582_Rpro	582_Rpro	582_Rpro
584_Rpro	584_Rpro	-	584_Rpro	584_Rpro	-	584_Rpro	584_Rpro	584_Rpro	584_Rpro
586_Rpro	586_Rpro	-	586_Rpro	586_Rpro	586_Rpro	586_Rpro	586_Rpro	586_Rpro	586_Rpro
588_Rpro	-	588_Rpro	588_Rpro	588_Rpro	588_Rpro	588_Rpro	588_Rpro	588_Rpro	588_Rpro
-	-	58_Rpal	58_Rpal	58_Rpal	58_Rpal	58_Rpal	58_Rpal	58_Rpal	58_Rpal
-	590_Rpro	590_Rpro	-	590_Rpro	590_Rpro	590_Rpro	590_Rpro	590_Rpro	590_Rpro
-	-	-	-	-	592_Recu	-	-		
-	-	-	595_Recu	-	-	595_Recu	-	595_Recu	595_Recu
-	-	-	-	-	-	597_Recu	-		
599_Recu	-	-	599_Recu	-	-	599_Recu	-	599_Recu	599_Recu
59_Rpal	-	59_Rpal	59_Rpal	59_Rpal	59_Rpal	59_Rpal	59_Rpal	59_Rpal	59_Rpal
5_Rpro	-	5_Rpro	5_Rpro	5_Rpro	-	-	5_Rpro	5_Rpro	5_Rpro
-	-	-	-	600_Recu	-	-	-		

28S	CISP	Cytb	LSM	PJH_V5	tRNA	UPCA_V5	UPMETAL	ALL	NUC
-	-	601_Recu	-	-	-	-	-		
-	602_Recu	-	-	602_Recu	602_Recu	-	-	602_Recu	602_Recu
-	-	603_Rsta	603_Rsta	-	-	-	-	603_Rsta	603_Rsta
-	604_Rsta	-	604_Rsta	-	604_Rsta	-	-	604_Rsta	604_Rsta
-	605_Rsta	605_Rsta	605_Rsta	-	-	-	-	605_Rsta	605_Rsta
-	606_Rsta	-	-	-	-	-	-		
-	-	-	607_Rsta	607_Rsta	-	-	-	607_Rsta	607_Rsta
-	608_Rsta	-	-	608_Rsta	-	-	-	608_Rsta	608_Rsta
-	609_Rsta	-	609_Rsta	609_Rsta	609_Rsta	-	-	609_Rsta	609_Rsta
60_Rpal	60_Rpal	60_Rpal	60_Rpal	60_Rpal	60_Rpal	60_Rpal	60_Rpal	60_Rpal	60_Rpal
-	-	-	-	610_Rsta	-	-	-		
-	613_Pter	-	613_Pter	613_Pter	613_Pter	-	-	613_Pter	613_Pter
-	614_Pter	614_Pter	614_Pter	614_Pter	614_Pter	614_Pter	-	614_Pter	614_Pter
-	615_Pter	-	615_Pter	615_Pter	615_Pter	-	-	615_Pter	615_Pter
-	616_Pter	-	616_Pter	616_Pter	616_Pter	616_Pter	-	616_Pter	616_Pter
-	-	-	-	617_Pter	617_Pter	617_Pter	-	617_Pter	617_Pter
61_Rpal	61_Rpal	61_Rpal	61_Rpal	61_Rpal	61_Rpal	61_Rpal	61_Rpal	61_Rpal	61_Rpal
-	623_Pter	623_Pter	623_Pter	623_Pter	623_Pter	623_Pter	-	623_Pter	623_Pter
-	625_Pter	-	-	625_Pter	625_Pter	625_Pter	-	625_Pter	625_Pter
62_Rpal	-	62_Rpal	62_Rpal	62_Rpal	62_Rpal	62_Rpal	62_Rpal	62_Rpal	62_Rpal
-	630_Pter	-	630_Pter	630_Pter	630_Pter	-	-	630_Pter	630_Pter
631_Pter	-	-	631_Pter	631_Pter	631_Pter	-	-	631_Pter	631_Pter
-	632_Pter	-	632_Pter	632_Pter	632_Pter	-	-	632_Pter	632_Pter
633_Pter	633_Pter	633_Pter	633_Pter	633_Pter	633_Pter	-	-	633_Pter	633_Pter
-	-	634_Pter	634_Pter	634_Pter	634_Pter	-	-	634_Pter	634_Pter
-	635_Pter	-	635_Pter	635_Pter	635_Pter	635_Pter	-	635_Pter	635_Pter
-	636_Pter	636_Pter	636_Pter	636_Pter	636_Pter	636_Pter	-	636_Pter	636_Pter
-	-	-	-	637_Pter	637_Pter	637_Pter	-	637_Pter	637_Pter
-	63_Rcol	63_Rcol	63_Rcol	63_Rcol	63_Rcol	63_Rcol	63_Rcol	63_Rcol	63_Rcol
-	-	-	647_Pcor	647_Pcor	647_Pcor	647_Pcor	-	647_Pcor	647_Pcor
648_Pcor	648_Pcor	648_Pcor	648_Pcor	648_Pcor	648_Pcor	648_Pcor	-	648_Pcor	648_Pcor
-	-	649_Pcor	649_Pcor	649_Pcor	649_Pcor	649_Pcor	-	649_Pcor	649_Pcor
-	64_Rcol	64_Rcol	64_Rcol	64_Rcol	64_Rcol	64_Rcol	64_Rcol	64_Rcol	64_Rcol
-	-	650_Pcor	-	650_Pcor	650_Pcor	650_Pcor	-	650_Pcor	650_Pcor
-	-	652_Pcor	652_Pcor	652_Pcor	652_Pcor	652_Pcor	-	652_Pcor	652_Pcor
-	654_Pcor	654_Pcor	-	654_Pcor	654_Pcor	654_Pcor	-	654_Pcor	654_Pcor
-	655_Pcor	655_Pcor	655_Pcor	655_Pcor	655_Pcor	-	-	655_Pcor	655_Pcor
658_Pcor	-	-	658_Pcor	658_Pcor	658_Pcor	658_Pcor	-	658_Pcor	658_Pcor
-	-	65_Rcol	-	-	-	65_Rcol	65_Rcol	65_Rcol	65_Rcol
-	660_Pcor	660_Pcor	660_Pcor	660_Pcor	660_Pcor	660_Pcor	-	660_Pcor	660_Pcor
-	-	662_Pcor	662_Pcor	662_Pcor	662_Pcor	662_Pcor	-	662_Pcor	662_Pcor
-	663_Pcor	663_Pcor	663_Pcor	-	-	663_Pcor	-	663_Pcor	663_Pcor
-	664_Pcor	-	664_Pcor	-	664_Pcor	664_Pcor	-	664_Pcor	664_Pcor

28S	CISP	Cytb	LSM	PJH_V5	tRNA	UPCA_V5	UPMETAL	ALL	NUC
665_Pcor	-	-	-	-	-	665_Pcor	-	665_Pcor	665_Pcor
666_Pcor	-	666_Pcor	666_Pcor	-	666_Pcor	666_Pcor	-	666_Pcor	666_Pcor
667_Pcor	667_Pcor	667_Pcor	667_Pcor	667_Pcor	667_Pcor	-	-	667_Pcor	667_Pcor
669_Pcor	669_Pcor	669_Pcor	669_Pcor	669_Pcor	669_Pcor	669_Pcor	-	669_Pcor	669_Pcor
66_Rcol	-	66_Rcol	66_Rcol	66_Rcol	66_Rcol	66_Rcol	66_Rcol	66_Rcol	66_Rcol
670_Pcor	-	670_Pcor	670_Pcor	670_Pcor	670_Pcor	670_Pcor	-	670_Pcor	670_Pcor
671_Pcor	671_Pcor	671_Pcor	-	-	671_Pcor	-	-	671_Pcor	671_Pcor
-	672_Pcor	672_Pcor	672_Pcor	672_Pcor	672_Pcor	672_Pcor	-	672_Pcor	672_Pcor
-	679_Pcor	679_Pcor	679_Pcor	-	679_Pcor	679_Pcor	-	679_Pcor	679_Pcor
67_Rcol	67_Rcol	67_Rcol	67_Rcol	67_Rcol	67_Rcol	67_Rcol	67_Rcol	67_Rcol	67_Rcol
680_Pcor	680_Pcor	680_Pcor	680_Pcor	-	680_Pcor	680_Pcor	-	680_Pcor	680_Pcor
-	-	-	-	-	681_Pcor	-	-		
682_Pcor	682_Pcor	-	682_Pcor	-	682_Pcor	682_Pcor	-	682_Pcor	682_Pcor
-	-	-	-	-	683_Pcor	683_Pcor	-	683_Pcor	683_Pcor
688_Pcor	-	-	688_Pcor	688_Pcor	688_Pcor	688_Pcor	-	688_Pcor	688_Pcor
689_Part	-	-	689_Part	689_Part	689_Part	-	-	689_Part	689_Part
-	-	68_Rpro	68_Rpro	68_Rpro	68_Rpro	68_Rpro	68_Rpro	68_Rpro	68_Rpro
690_Part	-	-	690_Part	690_Part	690_Part	690_Part	-		
-	-	690_part	-	-	-	-	-	-	-
691_Part	-	691_Part	691_Part	691_Part	691_Part	691_Part	-	691_Part	691_Part
-	-	-	-	-	692_Part	692_Part	-	692_Part	692_Part
693_Part	-	-	693_Part	693_Part	693_Part	693_Part	-	693_Part	693_Part
694_Pcor	694_Pcor	694_Pcor	694_Pcor	694_Pcor	-	694_Pcor	-	694_Pcor	694_Pcor
69_Rpro	69_Rpro	69_Rpro	69_Rpro	69_Rpro	69_Rpro	69_Rpro	69_Rpro	69_Rpro	69_Rpro
-	-	-	6_Rpro	6_Rpro	6_Rpro	-	-	6_Rpro	6_Rpro
-	703_Pter	703_Pter	703_Pter	703_Pter	703_Pter	703_Pter	-	703_Pter	703_Pter
705_Part	705_Part	705_Part	-	705_Part	705_Part	705_Part	-	705_Part	705_Part
706_Part	-	-	706_Part	-	706_Part	-	-	706_Part	706_Part
-	707_Part	-	-	707_Part	707_Part	707_Part	-	707_Part	707_Part
-	709_Part	-	709_Part	709_Part	709_Part	709_Part	-	709_Part	709_Part
-	70_Rpro	70_Rpro	70_Rpro	70_Rpro	70_Rpro	70_Rpro	70_Rpro	70_Rpro	70_Rpro
-	-	711_Part	-	-	-	-	-		
712_Part	-	712_Part	712_Part	712_Part	712_Part	712_Part	-		
-	-	712_Part.2	-	-	-	-	-	712_Part.2	-
-	-	715_Part	-	-	-	-	-		
719_Rpro	719_Rpro	719_Rpro	719_Rpro	719_Rpro	719_Rpro	-	719_Rpro	719_Rpro	719_Rpro
71_Rpro	71_Rpro	71_Rpro	71_Rpro	71_Rpro	71_Rpro	71_Rpro	71_Rpro	71_Rpro	71_Rpro
-	-	-	-	-	-	720_Rpro	-		
721_Rpro	721_Rpro	721_Rpro	721_Rpro	721_Rpro	721_Rpro	-	-	721_Rpro	721_Rpro
722_Rpro	722_Rpro	722_Rpro	722_Rpro	722_Rpro	722_Rpro	-	722_Rpro	722_Rpro	722_Rpro
723_Rpro	723_Rpro	723_Rpro	723_Rpro	723_Rpro	723_Rpro	723_Rpro	723_Rpro	723_Rpro	723_Rpro
726_Rpal	-	726_Rpal	-	726_Rpal	726_Rpal	-	-	726_Rpal	726_Rpal
-	727_Part	-	727_Part	727_Part	727_Part	-	-	727_Part	727_Part

28S	CISP	Cytb	LSM	PJH_V5	tRNA	UPCA_V5	UPMETAL	ALL	NUC
-	728_Part	-	728_Part	728_Part	728_Part	-	-	728_Part	728_Part
729_Part	-	-	-	729_Part	729_Part	-	-	729_Part	729_Part
72_Rpro	72_Rpro	72_Rpro	72_Rpro	72_Rpro	72_Rpro	72_Rpro	72_Rpro	72_Rpro	72_Rpro
731_Part	-	-	731_Part	731_Part	731_Part	-	731_Part	731_Part	731_Part
732_Part	732_Part	-	732_Part	732_Part	732_Part	-	-	732_Part	732_Part
-	733_Part	-	733_Part	733_Part	733_Part	733_Part	733_Part	733_Part	733_Part
734_Part	-	-	-	-	-	-	-		
737_Part	737_Part	-	737_Part	737_Part	737_Part	737_Part	-	737_Part	737_Part
738_Part	738_Part	-	738_Part	738_Part	738_Part	738_Part	-	738_Part	738_Part
-	-	73_Rpro	73_Rpro	73_Rpro	73_Rpro	73_Rpro	-	73_Rpro	73_Rpro
742_Part	-	-	-	742_Part	-	742_Part	-	742_Part	742_Part
74_Rpro	-	74_Rpro	74_Rpro	74_Rpro	74_Rpro	74_Rpro	-	74_Rpro	74_Rpro
-	-	751_Rpal	751_Rpal	-	751_Rpal	-	-	751_Rpal	751_Rpal
759_Part	759_Part	-	759_Part	759_Part	759_Part	759_Part	-	759_Part	759_Part
75_Rpro	-	75_Rpro	75_Rpro	75_Rpro	75_Rpro	75_Rpro	75_Rpro	75_Rpro	75_Rpro
760_Part	760_Part	-	760_Part	760_Part	760_Part	760_Part	-	760_Part	760_Part
-	761_Part	-	761_Part	761_Part	761_Part	761_Part	-	761_Part	761_Part
765_Part	765_Part	-	765_Part	765_Part	765_Part	765_Part	-	765_Part	765_Part
-	-	76_Rpro	76_Rpro	-	-	-	76_Rpro	76_Rpro	76_Rpro
774_Rpro	774_Rpro	774_Rpro	774_Rpro	774_Rpro	774_Rpro	774_Rpro	774_Rpro	774_Rpro	774_Rpro
775_Rpro	775_Rpro	775_Rpro	775_Rpro	775_Rpro	775_Rpro	775_Rpro	775_Rpro	775_Rpro	775_Rpro
777_Rpro	-	-	777_Rpro	777_Rpro	777_Rpro	-	-	777_Rpro	777_Rpro
77_Rpro	-	77_Rpro	-	-	77_Rpro	-	77_Rpro	77_Rpro	77_Rpro
781_Rpro	-	781_Rpro	781_Rpro	781_Rpro	781_Rpro	781_Rpro	-	781_Rpro	781_Rpro
78_Rpro	-	78_Rpro	78_Rpro	78_Rpro	78_Rpro	-	78_Rpro	78_Rpro	78_Rpro
79_Rpro	-	79_Rpro	79_Rpro	79_Rpro	79_Rpro	79_Rpro	79_Rpro	79_Rpro	79_Rpro
-	-	7_Rpro	7_Rpro	7_Rpro	-	7_Rpro	7_Rpro	7_Rpro	7_Rpro
80_Rpro	-	80_Rpro	80_Rpro	80_Rpro	80_Rpro	-	80_Rpro	80_Rpro	80_Rpro
81_Rpro	-	81_Rpro	81_Rpro	81_Rpro	81_Rpro	81_Rpro	81_Rpro	81_Rpro	81_Rpro
82_Rpro	-	82_Rpro	-	82_Rpro	-	82_Rpro	82_Rpro	82_Rpro	82_Rpro
85_Rneg	-	85_Rneg	85_Rneg	85_Rneg	-	85_Rneg	-	85_Rneg	85_Rneg
86_Rneg	-	86_Rneg	86_Rneg	86_Rneg	-	86_Rneg	-	86_Rneg	86_Rneg
-	-	87_Rneg	87_Rneg	87_Rneg	-	87_Rneg	-	87_Rneg	87_Rneg
-	-	88_Rneg	88_Rneg	88_Rneg	-	88_Rneg	-	88_Rneg	88_Rneg
89_Rneg	-	89_Rneg	89_Rneg	89_Rneg	-	-	-	89_Rneg	89_Rneg
-	-	-	8_Rpro	-	8_Rpro	8_Rpro	8_Rpro	8_Rpro	8_Rpro
90_Rneg	-	90_Rneg	90_Rneg	90_Rneg	-	90_Rneg	-	90_Rneg	90_Rneg
93_Rneg	-	93_Rneg	93_Rneg	93_Rneg	-	93_Rneg	93_Rneg	93_Rneg	93_Rneg
-	94_Rneg	94_Rneg	94_Rneg	94_Rneg	-	-	94_Rneg	94_Rneg	94_Rneg
95_Rneg	95_Rneg	95_Rneg	95_Rneg	-	-	95_Rneg	95_Rneg	95_Rneg	95_Rneg
-	96_Rneg	96_Rneg	96_Rneg	96_Rneg	-	-	-	96_Rneg	96_Rneg
-	99_Rrob	99_Rrob	99_Rrob	99_Rrob	99_Rrob	99_Rrob	99_Rrob	99_Rrob	99_Rrob
-	9_Rpro	-	9_Rpro	-	9_Rpro	9_Rpro	9_Rpro	9_Rpro	9_Rpro

28S	CISP	Cytb	LSM	PJH_V5	tRNA	UPCA_V5	UPMETAL	ALL	NUC
Out_Pans1	Out_Pans1	Out_Pans1	-	Out_Pans1	-	-	Out_Pans1	Out_Pans1	Out_Pans1
Out_Pans2	Out_Pans2	Out_Pans2	-	Out_Pans2	-	-	Out_Pans2	Out_Pans2	Out_Pans2
-	Out_Pans3	Out_Pans3	-	Out_Pans3	-	-	Out_Pans3	Out_Pans3	Out_Pans3
-	Out_Pans4	Out_Pans4	-	Out_Pans4	-	-	Out_Pans4	Out_Pans4	Out_Pans4
-	-	Out_Pans5	-	Out_Pans5	-	-	-	Out_Pans5	Out_Pans5
Out_Tri	Out_Tri	Out_Tri	Out_Tri	Out_Tri	-	Out_Tri	-	Out_Tri	Out_Tri
-	Out_Tri2	-	-	-	-	-	-	Out_Tri2	Out_Tri2
